# Classifying strategy use in multiattribute subjective choice: Application to conjoint experiments in political science

**DOI:** 10.1073/pnas.2613113123

**Published:** 2026-09-28

**Authors:** Nidhi V. Banavar, Kirk Bansak

**Affiliations:** ^a^ Department of Political Science, University of California Berkeley, Berkeley, CA 94720

**Keywords:** Bayesian cognitive modeling, conjoint experiments, multiattribute choice, political behavior, strategy use

## Abstract

Conjoint experiments are extensively used across multiple disciplines to make inferences about preferences over multiattribute alternatives. Much previous research has analyzed the importance of attributes at the aggregate level. Here, we focus on understanding individual-level behavior and, specifically, on strategy use. We present a Bayesian cognitive model of strategy use that performs attribute weight inference (how important an attribute is), attribute level inference (which value of the attribute is favored), and latent-mixture inference to classify strategy use among well-established strategies in cognitive science. We then validate and apply our model to two conjoint experiments (*N* = 3,762) focusing on three political domains (attitudes toward immigrants, political candidates, and climate policy). We find that the simplest heuristic strategy is the highest probability in all conditions and that approximately 67–76% of subjects are classified as using different strategies depending on the political domain. Our model additionally highlights how aggregate-level inference on the weight of an attribute can be meaningfully decomposed by individual differences in strategy use and preferences over attributes. In addition, our experiments include a set of experimental design manipulations that increase different dimensions of complexity and show their impact on strategy classification.

## Significance

Understanding how individuals navigate multiattribute decisions is essential for interpreting public preferences. While researchers frequently use conjoint experiments to measure these preferences, traditional approaches in the applied social sciences often rely on aggregate-level analyses that assume rational behavior and do not fully consider interindividual variability. We present a Bayesian cognitive model that classifies the diverse strategies individuals employ during these tasks. Applying this to political choice domains, we find that most people are classified as relying on simplified heuristics rather than exhaustive reasoning and adapt their strategies to specific contexts, varying strategies depending on experimental design and domain. These findings provide a framework for decomposing aggregate preferences into underlying cognitive components, enhancing the accuracy and interpretability of social science research.

Complex multiattribute choices are a ubiquitous part of everyday life. They can range from being highly impactful to putatively mundane, and pertain to diverse roles that individuals play in society—such as the role of a citizen [e.g., political candidate choice (1) and policy preferences (2, 3)], consumer [e.g., preferences for commercial products (4)], or student [e.g., roommate choice (5)]. Given the high frequency and potential importance of such decisions, researchers have developed several methods to understand those decisions and elicit individuals’ underlying preferences. One of the foremost tools for doing so in many disciplines is the choice-based conjoint experiment (6), in which subjects are asked to indicate their preference between two or more alternative options (e.g., two possible political candidates) that randomly vary along several attributes (e.g., gender).

Typically, the primary inferential targets of a conjoint researcher relate to subjects’ preferences over attributes and their levels (i.e., the different values an attribute may take), though a diverse set of approaches are employed across different disciplines in order to make those inferences. In marketing, for example, the current dominant approach is grounded in economic theory, involving the inference of partial utilities of each attribute (7) or more broadly invoking the random utility framework in the form of hierarchical multinomial regressions (8). Such models can be used to infer preferences ranging from the individual to the aggregate and have been applied in fields as diverse as healthcare, social psychology, and transportation (9–11).

The dominant approach in political science, another discipline where the conjoint is the subject of widespread application and methodological development, employs a more model-agnostic focus tied to causal inference. This approach typically emphasizes aggregate links to preferences. In particular, the average marginal component effect (AMCE) (12), which has become the most widely used estimand in political science, corresponds to the causal effect of an attribute level on choice and also formally represents a specific probabilistic aggregation of preferences (13). Recent research in political science has also proposed the analysis of preferences via alternative perspectives and estimands (e.g., refs. 13–16).

A distinct but intimately related question of interest involves strategy usage. That is, how do people evaluate, combine, compare, and cognitively engage with the multifaceted information presented to them in order to make the very choices indicative of preference? This is a critical consideration given that we do not have direct access to individuals’ latent preferences, assuming they exist. We can therefore only infer them through observed behavior, regardless of the mode of analysis being applied. Further, it is a well-known fact that the observed choice behavior and subsequent inferences can be, and often are, shaped by the elicitation method.

It is with this consideration that we focus specifically on strategy usage in conjoint experiments in this paper. We introduce a Bayesian cognitive model for classifying what strategies individuals use as they participate in conjoint experiments, and we apply and evaluate the model with a set of applications to political choice. We classify respondents’ strategies using a collection of well-established strategies in cognitive science. For example, two of the strategies that we consider include “Take The Best,” where an individual chooses the profile based on the one informative attribute they value the most, and “Weighted Additive,” where an individual rationally evaluates all information available to them (17). Another reason to explicitly model strategy use in the conjoint is that a number of dominant paradigms in conjoint analysis implicitly assume that individuals follow this rational strategy (18–20). Even when such an assumption is not formally made, there is still a tendency in applied research to interpret the results of conjoint experiments in ways that are suggestive of rationality. However, extensive previous research has demonstrated that it is common for people to employ heuristics when making decisions, as both survey respondents and decision-making agents in the real world (17). Furthermore, theory and empirics establish the existence of heterogeneity across individuals, including in strategy use, as a fact (e.g., refs. 17,22–23). Better understanding strategy usage in conjoint experiments therefore provides valuable information not just for survey and experimental methodologists but also for applied conjoint researchers interested in mechanistic questions around why they observe the behavior they do. For instance, by digging into subjects’ strategy usage at the individual level, we can elucidate the individual-level behaviors from which our downstream inferences about preferences can arise.

## Contributions.

This paper makes a number of contributions along both methodological and applied lines. Methodologically, we develop a psychologically interpretable Bayesian latent-mixture model that classifies strategy use during subjective multiattribute choice. Its construction as a Bayesian cognitive model gives the parameters explicitly psychological interpretations, allowing for intuitive inference not only on the strategy used by an individual. At the same time, we show empirically that our model also exhibits strong descriptive capability, demonstrating that its interpretability does not come at the cost of descriptive adequacy. Our model also represents an advance in the strategy modeling literature in cognitive science, where the focus is almost exclusively in objective domains (as elaborated further below). In addition, we extend and apply latent mixture strategy inference to the subjective political domain.

Straddling the applied and methodological perspectives, another contribution is that we provide a comprehensive evaluation of both within-individual and between-individual heterogeneity in strategy usage in conjoint experiments. To achieve this and to maximize our ability to detect individual-level heterogeneity, we take two important steps. The first is that we originate our modeling, estimation, and analysis at the level of the individual per se—that is, with respect to a single individual at a time, in contrast to modeling hierarchically or as a function of covariates. The second is that we apply and evaluate our model across a range of conjoint designs, with each respondent engaging in multiple decision domains. Specifically, we apply our model to conjoint experiments in three common issue areas in political science—decisions about which immigrants they would rather admit to the United States, which Senate primary candidate they would rather vote for, and which climate policy package they would prefer the United States to adopt—which allows us to make inferences about within-individual strategy use, i.e., considering whether the same person is classified as using the same strategy for every political domain. We additionally attempt to explore why people may use different strategies and what accounts for variation in strategy classifications in a number of ways, including demographic correlates and self-reported perceptions of the conjoint task. In addition, as previous research has highlighted how individuals may adaptively deploy strategies in response to complex decision environments (18, 21), we explore this possibility by manipulating multiple dimensions of complexity in the conjoint design itself: 1) allowing/restricting ties in attribute levels (i.e., stimulus complexity) and 2) including/excluding rating trials in addition to the choice-based prompts (i.e., goal complexity).

Finally, from an applied perspective, we provide evidence on strategy usage in political choice conjoints. This includes the distribution of strategy classifications across individuals, differential strategy classifications by the same individual, and a variety of determinants (and nondeterminants) of strategy. Most notably, we find a) a pervasiveness of heuristic strategies that remains relatively robust even in the face of multiple conjoint design elements that should directly counteract such heuristics, b) a common tendency for individuals to be classified as using different strategies in different scenarios, and c) a general inability to effectively prognosticate an individual’s strategy. Collectively, our evidence supports a modal tendency toward heuristics but also a propensity for individuals to vary the choice behavior they employ, albeit in largely unpredictable ways. In addition, we also find that the complexity of the design impacts strategy classification, providing a clear demonstration of how variation in strategy can be disconnected from respondents’ underlying preferences with respect to the alternatives being evaluated. Our results have important implications for understanding how to interpret and generalize results from conjoint experiments, such as how to consider contextualizing information or estimates that are aggregated at the population level, and for understanding survey-taking behavior more broadly.

## Related Work.

This study relates to but extends beyond a number of areas of research. First, strategy usage and strategy modeling in multiattribute choice are longstanding subjects of investigation in cognitive science. However, whereas our focus is on subjective decision-making, much of the existing work in this area has been applied to the objective domain where there are objectively correct or optimal responses to the decision problem (e.g., determining which city has the larger population) (24, 25). In contrast, the formal study of strategy use in subjective decision problems (e.g., deciding which city one would rather live in based on one’s preferences) has received much less attention within this framework. This is due at least partially to the greater difficulty of modeling and performing inference on subjective (vs. objective) decision-making, which will become clear later when we present our model and discuss its methodological contributions.

Second, there has been a limited amount of previous work exploring strategy specifically in the context of conjoint surveys. This includes ref. 19, which uses self-reports to study the distribution of strategy use in a conjoint experiment, finding that a majority of subjects use heuristics and not the rational strategy. In other work, Dieckmann et al. (20) compares the predictive accuracy of rational vs. heuristic preference models across different conjoint designs. More recently, Hjortskov and Andersen (26) uses standard political science conjoint design and analysis methods to reveal lexicographic strategy use via the presence of second-order attributes (i.e., attributes that become important when other, more primary, attributes are not informative). In addition, older research in the marketing literature, expressly designed for subjective multiattribute choice though not a conjoint instrument, introduces a latent class model to demonstrate that people’s strategy use changes as a function of environmental complexity, using an aggregate econometrics framework (18). We advance this literature by developing a model that is psychologically interpretable, incorporates and evaluates the goodness-of-fit for a richer variety of strategies all at once, and classifies strategy directly based on only choice behavior and at the individual level. The psychological interpretability of a Bayesian cognitive model lies in the fact that each parameter in the model has a clear psychological interpretation. For example, the posterior distribution over which strategy an individual is likely following corresponds to the base rate. The probability of success in our likelihood is interpreted as the fidelity with which an individual executes a given strategy. A model constructed in this fashion, therefore, has the power to provide inferences at multiple levels of psychological explanation in addition to the output of interest itself (i.e., the strategy classification).

Third, there is a rich literature in political science focused on the psychology of political choice and the political conjoint that has considered issues adjacent to strategy use, such as online updating, heuristic usage, satisficing, attention, and eye tracking (27–34). In contrast to these lines of work, we present a formalized cognitive model. Fourth, there is also a rich methodological literature focused on investigating heterogeneity across subjects in conjoint experiments in terms of their preferences with respect to (or the effects of) different attributes—in some cases using methods that estimate heterogeneity as a function of covariates and in some cases using methods that pursue individual-level inferences (e.g., refs. 7, 8, 15, and 35). We are similarly interested in heterogeneity across subjects, but as already indicated, our study departs from this line of work given our focus on strategy usage. Further, we note that we originate our modeling and analysis squarely at the individual level (i.e., with respect to a single individual at a time, without modeling hierarchically or as a function of covariates). Finally, due to the fact that we build a Bayesian model, we obtain a distribution over all possible strategy classifications for each subject. This allows us also to evaluate the posterior certainty over an individual’s strategy classifications.

## Results

### Strategies, Cognitive Modeling, and Experiments.

To study strategy usage, we build a Bayesian cognitive model for subjective multiattribute choice. This is a latent mixture model that classifies strategy usage at the individual level. Our modeling framework is intrinsically flexible in terms of how many strategies it can include, though we focus on four possible strategies that have been well characterized in the decision and political science strategy literatures. In order of reducing computational requirements, the four strategies are Weighted Additive (WADD, the rational strategy), Tally, Take the Best (TTB), and Contaminant. Table 1 provides summary descriptions of each strategy. As a Bayesian latent-mixture model, the model infers the posterior distribution over strategies for each individual. The model also infers how much weight an individual assigns to the attributes presented, in addition to what levels of the attribute are more favored by the subject. For more information on our strategy selection and cognitive modeling, see *Materials and Methods*.

**Table 1.** Strategies modeled

Strategy	Summary	Full Info	Description
Weighted Additive (WADD)	Weighted sum of attributes	Y	Individuals allocate a weight to each attribute and choose the profile with the highest numerical value, based on the attribute weights and their favored attribute levels. Rational strategy.
Tally	Unweighted sum	Y	Individuals count the total number of attributes with the preferred level that each profile contains and choose the profile containing the greater number. Heuristic strategy.
Take the Best (TTB)	Highest weighted attribute that is discriminable	N	Individuals pick the profile that has their preferred level for their most important attribute (and, if the levels are the same for the profiles, choose based on the next most important and informative attribute). Heuristic strategy.
Contaminant	Random guessing	N/A	Individuals’ behavior is best explained by having a 50–50 chance of picking either profile.

Subjects can be classified as following one of these strategies as they make their decisions. Choice according to any given strategy is deterministic where subjects pick the profile meeting the rule specified in the Description column: the profile with the highest value, where applicable, is chosen. Some strategies evaluate all attributes (Full Information = Y) and some only subsets (Full Information = N). An attribute is informative (discriminable) if it possesses different levels for each profile presented (e.g., Age for Profile 1 is 50 and Profile 2 is 70).

We apply our model to a set of choice-based conjoint experiments that we designed and implemented within three common issues areas in political science: asking individuals to make decisions about which immigrants they would rather admit to the United States (Immigration), which Senate primary candidate they would rather vote for (Candidate), and which climate policy package they would prefer the United States to adopt (Climate). In each conjoint trial, respondents were shown two profiles (e.g., two hypothetical immigrants) in a standard tabular form and asked to choose the profile they preferred (i.e., choice trials, *SI Appendix*, Fig. S1). We ran two experiments (both featuring all three domains and using a design with binary attributes) in which we randomized additional design features: in Experiment 1 (E1), we randomized whether or not an attribute’s level could be the same across both profiles, and in Experiment 2 (E2), we randomized whether or not subjects were asked to individually rate each profile in addition to choosing their preferred profile. In both experiments, all subjects completed three blocks of 30 trials each, where each block corresponded to the three decision domains presented in a randomized order.

### Strategy Classification.

Below we analyze the results on strategy classification in our conjoint experiments. Recall that our model outputs a posterior distribution over all possible strategies for each given subject in each given domain. There are therefore two ways in which we report results on strategy classification. First, we explicitly account for variability in the posterior distribution by sampling strategy classifications directly from the posterior chains and then perform subsequent analyses, such as taking the mean classification proportions from the sample (i.e., over repeated samples). Second, for certain evaluations, we consider only each subject’s posterior modal classification (i.e., the strategy with the highest probability for a subject given the observed data and prior) and then perform subsequent analyses. We begin by focusing on the results of E1 for brevity and show in the *SI Appendix* how E2 replicates our findings. We then consider the degree to which various factors—including experimental design features—relate to and modulate strategy use, which will include in-depth analysis of both E1 and E2.

#### Distribution of strategy use.

**Fig. 1.**
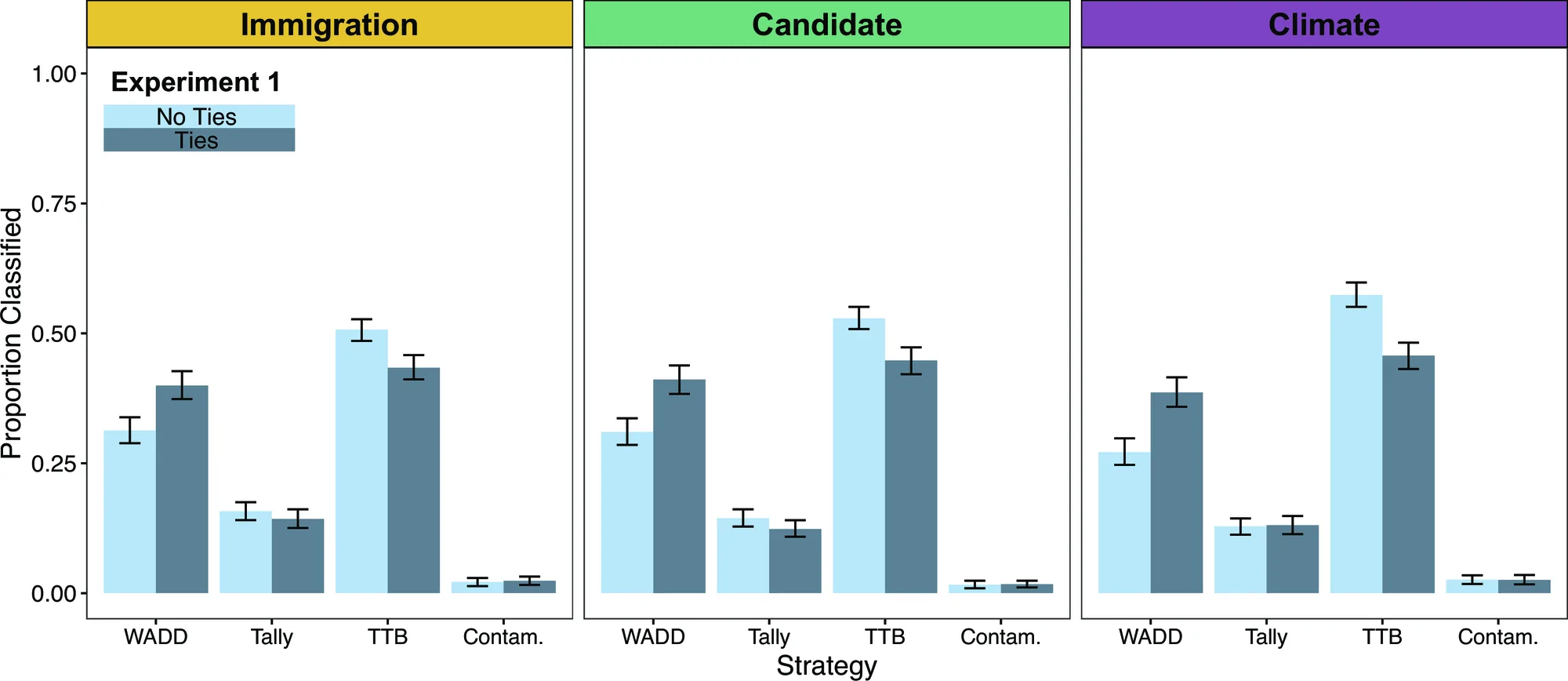
Heterogeneity in strategy classification. The heuristic TTB has the highest classification probability overall. The next most probable strategy is the rational WADD. We also find statistically significant differences in strategy classification as a function of whether there are ties present in the conjoint design. To perform this estimation, we randomly sample classifications from the posterior 1,000 times for each subject and compute the proportions of strategy classification across subjects for each of the 1,000 iterations (i.e., proportion TTB, WADD, for iterations 1, 2, etc.). The point estimates denoted by the height of the bars in the figure are computed as the mean of the proportions classified across the 1,000 iterations. The intervals shown in the figure are 95% credible intervals that are constructed using the percentiles of the iterations.

In considering the distribution of strategy classifications among subjects, we find strong general patterns across domains and experimental conditions, in addition to some notable differences. The results are shown in Fig. 1. The clearest substantive result across the board is the domination of TTB, the most frequently classified strategy: after sampling 1,000 times from each subject’s posterior distribution and then calculating the mean proportion of subjects classified into each strategy, we found that on average 68% of subjects in Condition 1 (no ties) and 57.9% of subjects in Condition 2 (ties) were classified as using heuristic strategies—either TTB or Tally. This was primarily driven by the widespread classification into TTB, accounting for 53.7% of subjects in Condition 1 and 44.6% of subjects in Condition 2, with TTB also being the highest probability strategy across all domains in both conditions. The next most probable strategy in all cases is the normative WADD: 29.8% in Condition 1 and 39.9% in Condition 2. These results are qualitatively similar across all domains, though strategy classification was most concentrated in the climate domain (see *SI Appendix* for a formal analysis).

**Fig. 2.**
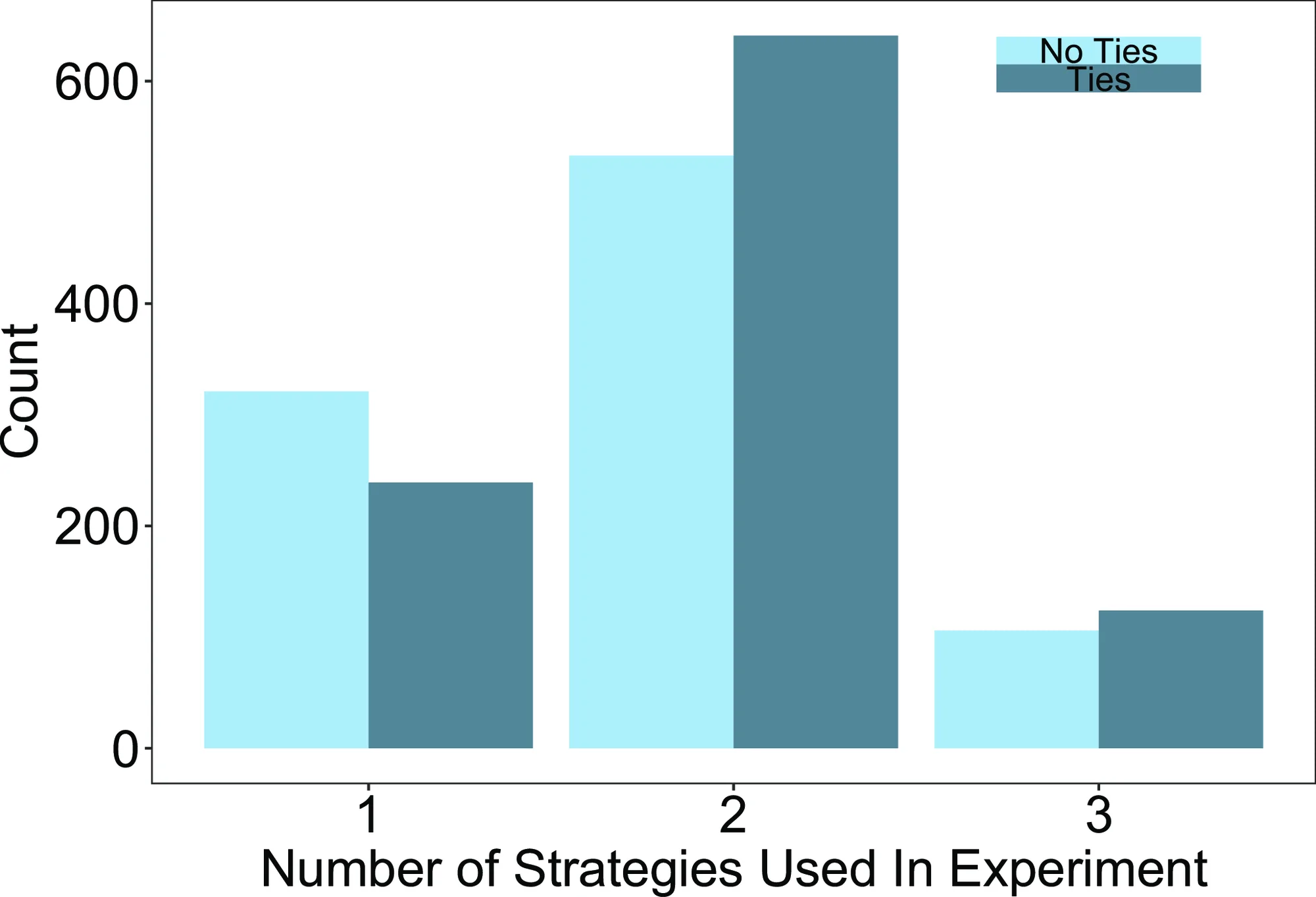
Most subjects are not classified as using the same strategy for each decision domain. Subjects were directed to make choices in accordance with their preferences, with no explicit instructions given about how they should choose. Here, we consider the modally classified strategy for each subject in each domain, treating strategy classification as fixed for each subject in each domain to conduct statistical inference (as opposed to Fig. 1). Subjects were significantly more likely to be classified as using more strategies across domains in the Ties condition compared to the No Ties condition (Bayesian *t* test, Bayes Factor = 126).

A key contribution of this work is to examine individual differences in subject behavior, in this case, in strategy use. While the results above demonstrate heterogeneity across individuals by domain, we now consider the question of heterogeneity within individuals: does the domain problem they are considering shape their strategy classification? We find that the majority of our subjects are not fixed in their inferred strategy, as determined by the modal classification, across all decision domains (66.6% Condition 1 and 76.2% Condition 2), as shown in Fig. 2. Indeed, a little over 10% of subjects in both conditions had a different modal strategy classification for all three domains. Note that the order in which each domain was presented was randomized across subjects, and we additionally found no systematic classification into the TTB or Contaminant strategies in the third block, thus allaying concerns about experiment fatigue systematically facilitating the use of heuristic or guessing strategies. This is an important finding for two reasons. First, strategy classifications do not appear to be a fixed personal characteristic even in the meta-domain of “political decision-making”: the evidence highlights that people pick their strategy to adapt to different specific decision contexts. Second, it also demonstrates the prevalence of TTB is not simply a matter of people being general survey satisficers: if so, we would expect substantially more strategy stickiness for the TTB subjects.

### What Modulates Strategy Use?

We now consider how experimental design features and subject characteristics relate to and modulate strategy use.

#### Experimental complexity.

It is well established that strategy use can vary as a function of experimental design (36). We therefore consider whether our two experimental manipulations lead to statistically different distributions of strategy classifications. Recall that in E1, our primary manipulation was the inclusion or exclusion of attribute level ties, thereby experimentally varying the degree of complexity in the information stimulus. In Fig. 1, we highlighted differences in the probability of a particular strategy as a function of including ties. We find that the presence of ties shifted people to be classified as using more full information strategies, with TTB becoming less likely and WADD becoming more likely (statistically significant at *P* < 0.001 for all three domains). One possible reason for the general shift toward a more complete strategy is that the inclusion of ties creates an environment where heuristics that consider only a subset of information may not offer as much value as they ordinarily might—a heuristic is “fast and frugal,” but an environment which demands the evaluation of up to 4 out of 6 attributes per decision (i.e., when ties are included) might instead reward a more complete strategy. That being said, we note again that TTB remains the dominant strategy classification even in the presence of ties.

**Fig. 3.**
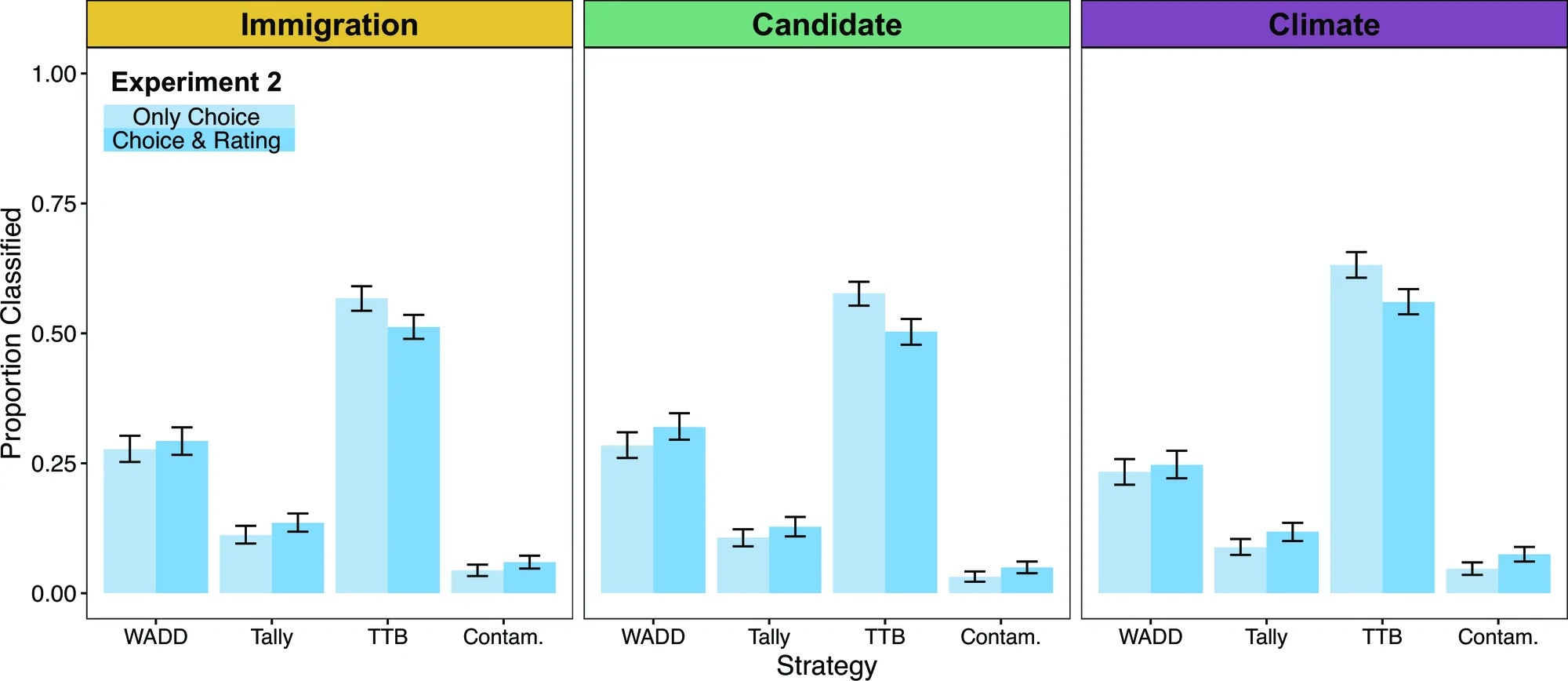
Including profile ratings in addition to choice shapes strategy use. Just as with E1, the simplest strategy, TTB, dominates in all four experimental conditions. The second most commonly classified strategy is the rational WADD. The inclusion of rating trials reduces the frequency of TTB use. We use the same process as in Fig. 1 to construct the plot and CIs.

To explore the effects of experimental design further, we designed and administered a second, similar conjoint experiment (E2). The design manipulation in this case was the inclusion of rating trials in addition to choice trials (Condition 2; Condition 1 was exactly the same as in Experiment 1 Condition 1), thereby experimentally varying the degree of complexity in the goal of the individual. Rating trials are frequently included in applied conjoint experiments and require individuals to assign a numerical value to each profile in addition to making their choice. We speculated that the inclusion of these rating trials might lead subjects to favor more full information strategies. This was, indeed, the case: the probability of TTB decreased across all three domains with statistical significance at *P* < 0.001 in all three domains (Fig. 3), and the majority of subjects continued to be classified as using two or three different strategies across decision domains (*SI Appendix*, Fig. S25). Interestingly, we also found that this shift away from TTB seems to be accompanied by two opposing forces: on the one hand, increased classification into the full-information strategies (WADD and Tally) for some subjects, and on the other, an increase in subjects being classified as Contaminant. These results hint at individual differences in response to the added complexity: while most subjects appropriately adhere to the task, a smaller group apparently loses interest or attention rather than engaging in more cognitively demanding processing. We caution that while these findings are qualitatively similar across domains, their statistical significance is not consistent and robust.

#### Subject characteristics.

We also perform exploratory evaluations to see whether different subject characteristics are associated with different strategy classifications. We examine subject demographics and their self-reported perceptions about the three decision domains they make decisions about. We focus particularly on the usage of TTB as this was the most ubiquitous strategy across all decision domains and experimental conditions. We begin with evaluating its association with subject demographics.

##### Demographics.

We fit a total of 12 linear regressions (4 experimental conditions across 3 decision domains) to assess whether posterior strategy classification is predicted by demographic variables. Our predictors include age, partisanship score, education, ability to vote, race, and gender. We report results from models that include all these variables in a single regression, though we note that we also implemented regressions separately for each predictor with little change in our substantive results. In order to make use of posterior uncertainty in strategy classification, we set the dependent variable to be the proportion of posterior weight assigned to a given strategy for that individual in a given domain. Concretely, assume subject *x* in the immigration domain has 75% of posterior mass on TTB. Their dependent variable would therefore be 0.75 in the linear regression for the immigration domain. We choose this dependent variable to preserve posterior distributional information compared to simply taking the modal strategy classification (which in the case of subject *x* is TTB) and fitting binary classification models.

We report both uncorrected and corrected results, using the conservative Bonferroni–Holm correction (*m* = 12 test correction, 4 experimental conditions × 3 decision domains). Age is the most consistently significant covariate before multiple comparison corrections. Specifically, the older the individual, the more likely they are classified as using TTB. This result was statistically significant in the uncorrected results 7 out of 12 times (precorrection, 0.001≤β≤0.003, P<0.05: E1C1 candidate, E1C2 candidate and climate, E2C1 candidate and climate, E2C2 candidate and climate). However, this result is statistically significant after correction for only two tests (E2C2 climate, β=0.003,P≤0.001, E2C1 climate, β=0.002,P<0.01). In addition, we see that the regression coefficients are consistently small, suggesting that any effect of age on being classified as using simpler strategies that might exist is small. While we find some other statistically significant results, they are generally inconsistent in that they occur infrequently (≤2 times out of 12), suggesting that these results could be artifacts.

##### Subject perceptions of decision domains.

We perform similar regression analyses using subjects’ self-reported ratings on how meaningful the decision domain was to them as independent variables. Specifically, subjects provide a number between 0 (not at all) to 10 (the most possible) on 1) how interested they were in the domain, 2) how informed they were about the domain, 3) how important the domain was, and 4) how complicated the decision domain was (see *SI Appendix*, section D.4 for more detail). We follow the same procedure as with the demographics analysis. Namely, we report results from models that include all these variables in a single regression (and as with the demographics, the results are largely stable when we fit with one predictor at a time). We report uncorrected results in the following paragraph, noting that only one test survived our conservative Bonferroni–Holm correction (m=12 test correction).

The first result of interest involves the perceived complexity of the decision domain. The less complex an individual rated the decision domain, the more likely they are classified as using TTB, with statistical significance 4 out of 12 times (precorrection, −0.015≤β≤−0.012,P<0.05: E1C1 immigration and candidate, E2C2 immigration and candidate). We also find signs that the less informed a person reported they were, the more likely they are to be classified as using TTB, though with statistical significance only 2 out of 12 times, all in the climate domain (precorrection, −0.02≤β≤−0.01,P<0.05: E2C1 climate, E2C2 climate). This analysis produces suggestions of how perception of the decision problem may influence strategy use, though additional study is necessary to better understand these interactions, particularly TTB usage as a function of lack of knowledge in the climate domain—the domain individuals reported was the most complicated and they were least informed about (*SI Appendix*, section D.4).

### Attribute Weight Distribution.

As described earlier, a crucial component of our model involves inferring how much weight an individual assigns to each attribute in a decision domain, as well as what levels of that attribute are favored by the individual. For example, how much does an individual care about the prior political experience of a political candidate, and do they prefer candidates with more or less experience? Here, we examine the distribution of attribute weights across subjects in order to demonstrate how variation in strategy use across individuals can shape aggregate-level inferences about the importance of a given attribute level. In addition, we further break down the distribution of weights as a function of 1) strategy use and 2) which attribute level was favored.^*^ As in the previous section, we focus on the results of E1 for brevity and show in *SI Appendix* how E2 replicates our findings.

**Fig. 4.**
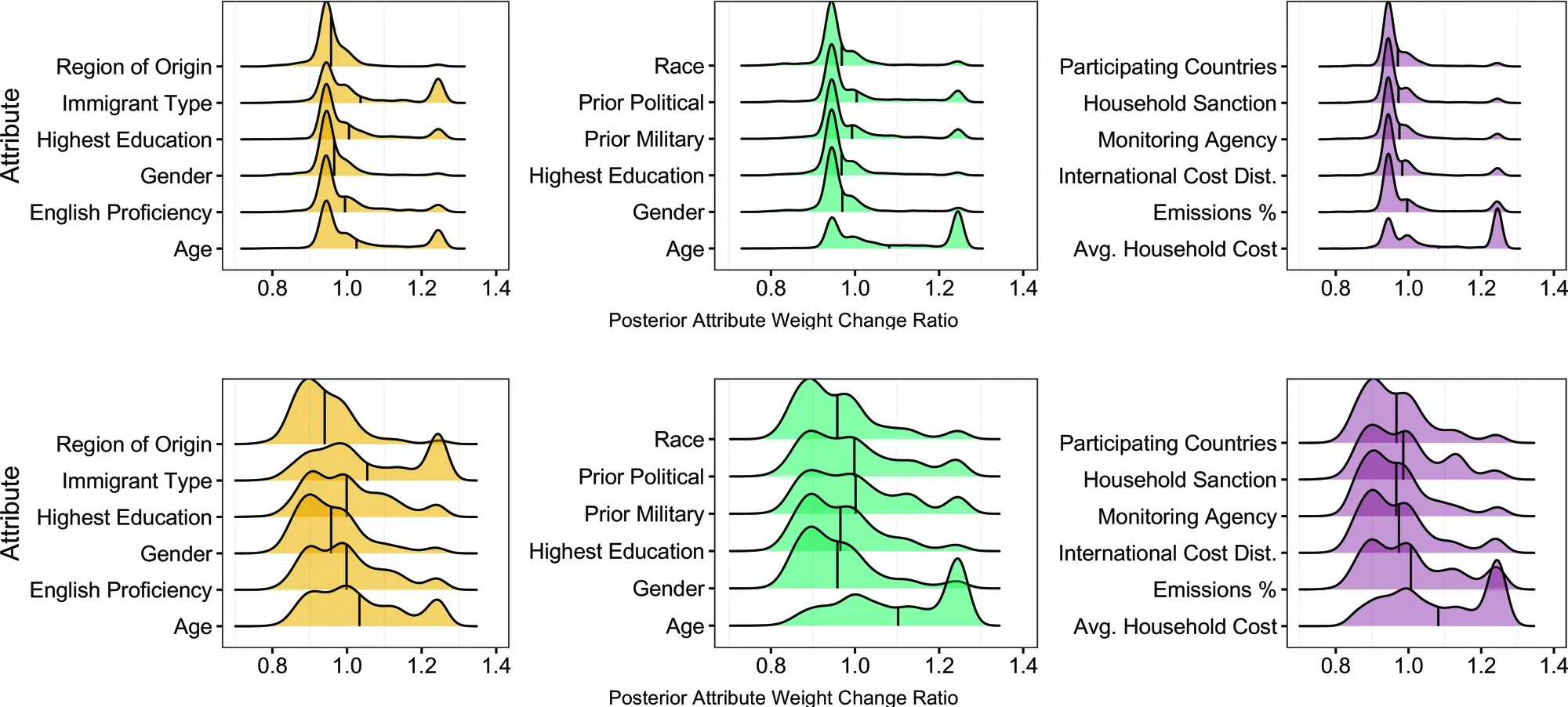
Distribution of posterior attribute weights demonstrates heterogeneity across individuals. We plot posterior attribute change ratios on the x-axis, where a value of 1 means that the prior and posterior mean weight are the same (i.e., no updating), a value greater than 1 means that the posterior mean is greater than the prior mean, and vice versa. The black line represents the mean posterior weight across subjects. Yellow graphs show results for the Immigration domain, green for Candidate, and purple for Climate.

First, note that the weights inferred by our model, while statistically interpretable, are not directly interpretable from a substantive perspective. In order to perform a more intuitive analysis of the distribution of weights across subjects, we construct an individual-level measure that compares an attribute’s posterior mean weight to its (uninformative) prior mean weight by taking the ratio of the two. A value below 1 indicates a posterior down-weighting for an attribute; because the priors are uninformative, this effectively corresponds to a subject giving negligible consideration to the attribute in their choices. In contrast, values above 1 indicate posterior up-weighting, and hence a subject giving an attribute consideration. Fig. 4 plots the between-subject distribution of this posterior/prior mean change ratio for each attribute in each domain and condition. There are two key findings. The first is that for all attributes, there is a substantial amount of density below 1. Indeed, the between-subject mean values are below 1 for 19 attributes (both conditions), indicating that for each of those attributes, the preponderance of subjects effectively places no weight on it in making their choices. Second, Fig. 4 also demonstrates clear multimodality in the attribute weight assignments, which reduces to striking bimodality for several attributes when attribute ties are restricted—particularly the attributes with the highest weights for each domain (*SI Appendix*, section B.3). This second result indicates that a sizable group of subjects place a very high weight on those attributes, driving the average posterior weight up.

Another notable result from Fig. 4 is the more diffuse spread of attribute weights in the Ties condition (Fig. 4*B*) relative to the No Ties condition (Fig. 4*A*), which is unsurprising (and effectively guaranteed by design) as subjects are always presented with at least one tied attribute on any given trial in the Ties condition. This necessitates that subjects engage with more attributes even if they use the “simplest” strategy TTB. Recall that the decision rule for TTB involves choosing the profile with the highest attribute weight and distinct attribute levels, which could mean evaluating up to four attributes on a given trial in the Ties condition, compared to just one attribute in the No Ties condition. Interestingly, when we asked subjects to explicitly indicate how much weight they put on each attribute, the subject-provided ratings do not demonstrate the stark multi- and bimodality that we see with the model-inferred weights (*SI Appendix*, Fig. S15). This highlights some of the limitations subjects have when directly articulating the degree to which they incorporate multiple factors into their decision process.

Overall, these results have important implications for the psychological and statistical inferences we can draw using aggregate estimates to infer the significance of an attribute. Not only does the model infer differential attribute weights across individuals, but the distributions of those weights are also far from exhibiting simple, well-behaved unimodality. Many subjects are inferred to put effectively no weight on a large portion of attributes they are asked to consider. We dive even deeper into these results by directly analyzing them in the context of strategy classification in *SI Appendix*, section D5.

## Discussion

Above, we examined both between-subject and within-subject heterogeneity in strategy classifications in conjoint experiments. We acknowledge that full generalizability has its limits given that we use a simplified experimental design, including binary attribute levels only, but nevertheless there are several findings and implications of important value to researchers who use conjoint surveys.

### Generalizable Findings.

The first finding relates to the pervasiveness of heuristics. While the precise estimated pervasiveness of TTB is undoubtedly context dependent, the fact that it is consistently the most classified strategy in our experiments, regardless of complexity manipulations, is striking. Broad usage of TTB could also provide a compelling explanation for various types of robustness in conjoint results that other studies have found when varying the complexity of several conjoint design features (including number of tasks, number of profiles, number of attributes, and inclusion/exclusion of odd attribute pairings) (32, 33, 37, 38). As such, our study provides foundational evidence to help connect together and explain the cognitive source of that robustness. The second generalizable finding is that strategy classifications are not fixed for respondents. There is no feature about our design that would lead to the theoretical expectation of within-person strategy variation only for our design and not for others. This has important implications for how we understand survey-taking behavior at the individual level—specifically, how we should not necessarily expect the level of attention or sophistication one gives to one part of a survey to be the same across other parts or, importantly, other decision domains. Finally, the third generalizable finding is that the complexity of the design impacts strategy classification. This result adds to the abundant existing evidence demonstrating how strategy usage and consequent inferred preferences can be shaped by experimental structure, suggesting inherent limits in generalizability.

### What Our Classifications Identify.

In interpreting our results and considering our findings above, we must be careful in explicitly delineating the scope of our claims. In particular, given that our model works from choice data, we must grapple with important identifiability issues related to the degree to which choice data alone can reveal the cognitive strategy used by a respondent, as opposed to classifying the strategy whose predictions happen to match their choices. In other words, what is and what is not identified by our classifications, and what are the subsequent implications for our findings above? In particular, there is a potentially sharp observational equivalence problem when trying to distinguish between respondents using TTB and certain respondents using WADD with extremely unequal attribute weights, especially when there are no ties in the attributes, as both can predict the same choice behavior. This is an issue that must be taken seriously, and we are careful not to claim that our classifications constitute direct observations of cognitive process. At the very least, our model identifies, with quantified posterior uncertainty, the strategy whose choice predictions can account for each respondent’s behavior. Yet we also believe our data and further analyses—including our process data on response times (RT) and simulation analyses—allow us to say more than this purely choice-descriptive minimum, as well as provide further support for the findings we describe above.

To begin, the first key question is whether our model can recover strategies appropriately from finite data when strategies have distinct predictions. That is, does the model perform as it should on the basis of what is actually identifiable from finite choice data? To validate the model’s performance, as well as probe into the model’s behavior in situations where there is observational equivalence between different strategies (namely TTB and extreme-weight WADD as well as Tally and uniform-weight WADD), we devised and conducted simulations across a wide variety of scenarios. Overall, we find that the model does behave as it should—it consistently classifies subjects as the strategy that best describes the simulated choice behavior. This is straightforward in the case of TTB and Tally. For subjects simulated from the WADD strategy, we see a sensitivity to the structure of the attribute weights. That is, when weights are compensatory, the choice behavior is usually classified as WADD; when weights are noncompensatory, the choice behavior is usually classified as TTB; and when weights are uniform, the choice behavior is usually classified as Tally. We elaborate further below on why we interpret this behavior as the model doing what it is supposed to do, despite the putative misclassifications. Finally, in our recovery analysis for contaminant data, generated by a coin flip, we find that the majority of Contaminant subjects are actually classified as Tally. This suggests that in our empirical results, we are underestimating contaminants and overestimating the prevalence of Tally. As an important implication of this, among subjects employing a substantive (i.e., noncontaminant) strategy, TTB-like behavior may explain an even greater proportion of choice behavior than our results suggest. For more details on the simulations and their results, see *SI Appendix*,section C.

This leaves open questions about how to treat and interpret our results in situations where different strategies (namely TTB and extreme-weight WADD, as well as Tally and uniform-weight WADD) predict the same behavior. On the one hand, we might acknowledge this observational equivalence as simply a limit of our enterprise and accept that our object of inference need not be a cognitive process per se but rather behavior that is consistent with it. As described above and in greater detail in *SI Appendix*, section C, in situations where extreme-weight WADD yields that same choice behavior as TTB, our model will classify a respondent as TTB (similarly for uniform-weight WADD and Tally). Such (mis)classification dynamics are arguably desirable from the perspective of inferring behavior consistent with different cognitive processes. From both philosophical and practical perspectives, one might ask what the real difference is between using a compensatory rule whose nonprimary attributes never change the outcome and a lexicographic rule that never consults those nonprimary attributes. The findings we present above can at a minimum be viewed through this lens, with behavior consistent with a strategy being the operative quantity of interest. Yet we also have reason to believe on the basis of our RT analysis that our data reach higher, carrying information about process and not only about choice.

As described in detail in *Materials and Methods*, our RT analysis provides strong corroborating evidence that our strategy classifications are indeed capturing process-level signal. Specifically, and to summarize: a) the RT distributions are increasingly compressed to the left (i.e., respondents take less time) for respondents classified as using strategies that entail less complex processes, and b) the only RT distribution for which there is evidence of distributional shift when moving from the No Ties to Ties condition is the one strategy (i.e., TTB) for which such a shift is expected from a process perspective. Both of these patterns are what a genuine process account predicts, and neither follows from a purely choice-descriptive account.

Going beyond the RT validation presented in the Materials and Methods, our RT evidence also allows us to moderate the concern raised above about extreme-weight WADD respondents being (mis)classified as TTB. Two questions that arise are whether such misclassification is pervasive enough to matter at a practical level, and whether it could generate the observed shift in the TTB RT distributions through a change in classification composition across the conditions. With respect to the first question about the pervasiveness of such misclassification, this can be partially and indirectly assessed by evaluating the shape of the TTB RT distribution. As already discussed, while extreme-weight WADD and TTB may produce identical choice behavior, what distinguishes them is the decision-making process—i.e., integrating information about many attributes into the decision (even if one is weighed much more heavily) vs. consulting only one attribute. In the no-ties condition in particular, the former process should take reliably longer than the latter. A group classified as TTB that is substantially contaminated with misclassified WADD respondents would hence be mixing two processes with meaningfully different complexity and time requirements, which should lead to a bimodal RT distribution. As shown in Fig. 6, there is no clear evidence of such mixing in our own data. In sum, misclassification pervasive enough to drive our results is difficult to reconcile with the RT evidence.

In considering the second question about whether the shift in the TTB RT distributions across conditions might also be attributable to differential misclassifications and subsequent composition changes, we can again evaluate the RT distribution evidence. Since assignment to the No Ties vs. Ties conditions is random, the two conditions draw the same mix of respondents in expectation, but it may be possible that the model varies in how it classifies those types across conditions. For instance, it may be that certain extreme-weight WADD respondents are misclassified as TTB under no-ties and then correctly classified as WADD with ties. However, a dynamic like this is also difficult to reconcile with the RT evidence, where only the TTB distribution changes across conditions, as described and formally tested in *Materials and Methods*. Any sort of composition change that leads one strategy classification group’s RT distribution to shift should be expected to shift at least one other group’s RT distribution, since composition changes entail “migration” between at least two groups. In addition, we might also expect that if such a dynamic were at play, the distinctive type of respondents involved in the migration might be concentrated in a certain part of the RT distribution. This would then lead to the thinning out of a more localized part of the TTB RT distribution. What we see, however, is a TTB RT distribution that is essentially uniformly shifted over when moving from the No Ties to Ties condition. Such a smooth shift is more consistent with a change in processing, not a change in the composition of who is being described.

Taking all of these considerations together, we wish to carefully calibrate our claims. Our strongest inferences concern choice behavior and what we can say about the consistency of cognitive strategies with that choice behavior. We find that behavior consistent with a simple, noncompensatory strategy best predicts the choices of the plurality of respondents, that the best-fitting strategy frequently switches across domains for the same respondent, and that conjoint design complexity systematically shifts strategy dynamics across respondents. These conclusions hold, and they lead us to the same methodological and substantive implications that we draw for the interpretation of conjoint experiments, regardless of one’s position on whether choice-based classification can ever fully pin down cognitive mechanisms. We more cautiously advance our claims about the actual underlying cognitive processes behind our data, yet as described above, we do believe we have some amount of evidence that our data do contain important signal about process, not just choice.

### Implications for Aggregate Estimates.

We also demonstrated how the differential evaluation of attribute weights (i.e., in line with different strategies) and attribute levels (i.e., which level of the attribute was more favored by the subject) coalesced to explain the aggregate results. This heterogeneity resulted in multimodal distributions of attribute weights for each domain. Our model provides an approach, and our analyses a foundation of evidence, to begin to better understand how survey respondents express their preferences during political choice, along with the subsequent implications for drawing high-level inferences from aggregate results. For instance, the prevalence of TTB classifications suggests that researchers should not look at a set of average attribute effects (e.g., the full set of AMCEs in a given conjoint experiment) and view those collective results as being a unified representation of a “typical” subject or even a specific subgroup of subjects. Though limited to only a few decision domains in our study, the consistency of our evidence across all of our domains and conditions suggests there may be similar patterns of behavior in other realms as well.

The empirical demonstration of heterogeneity in strategy use highlights that by digging into subjects’ strategy classifications at the individual level, we can elucidate the upstream individual-level behaviors from which our aggregate inferences can arise. For instance, consider the typical situation in which a particular attribute is found to have a modestly sized marginal effect (or partworth utility) on average. As one concrete example, a common finding from political science conjoint experiments is that female political candidates are slightly more preferred to male candidates across voters on average (39). What is not well-known, however, is what individual-level preferences and/or survey-taking behavior lead to this aggregate-level finding. Our results demonstrate heterogeneity in both preferences over gender (some prefer women and some men) as well as multimodality in the weight put on gender in general, and that mere countervailing, while present, does not sufficiently explain observed behavior.

Our results also have important implications for interpreting conjoint results involving multiple attributes. For instance, consider a widespread finding from political science conjoint experiments on attitudes toward immigrants—a finding that is indeed replicated again in our own application in this study—whereby subjects on average prefer refugees over economic migrants, but at the same time they also simultaneously prefer migrants with higher levels of economic skills relative to less-skilled migrants on average (40–43). These two results may seem to be contradictory, insofar as one might also assume that economic migrants are likely to (in general) be more skilled than forced migrants. Such a contradiction could be explained by at least two possibilities, which have quite different implications for respondents’ levels of cognitive sophistication and preference patterns when evaluating immigrant profiles. First, it could be that respondents are trying to simultaneously satisfy both a humanitarian impulse to prefer refugees and an economic impulse to prefer more skilled immigrants. On the other hand, however, it could also be that a preponderance of respondents are employing the much simpler strategy: some making decisions only based upon a humanitarian impulse and others making decisions only based upon skills-based calculations. The results from our study suggest the latter possibility to be the primary driver.

We also highlight in our applications how the model could be used for various conjoint methodological and design considerations. On the design side, considering how different designs can lead to different types of heuristics, we directly demonstrate this via experimental manipulations of the conjoint randomization scheme (including attribute level ties or not) and question type (choice only vs. rating and choice). On the analysis side, there has been great interest in deriving different conjoint estimands and exploring how they represent different aggregations of underlying preferences. Our results highlight the possible value of taking the converse approach: building a model, defining estimands, and analyzing data at the individual level and only then aggregating. For example, consider the multimodal attribute weight distributions: beginning with aggregate inferences on the importance of an attribute and its levels is like seeing the tip of the proverbial (subjective multiattribute) decision-making iceberg. This information is important, especially if researchers are interested in making population-level inferences, but tells far from the complete story and can even be potentially misleading if not properly interpreted. A related benefit in beginning at the individual level is that one can then choose the aggregation mechanism that is most appropriate for their inferential goals. Continuing with the attribute weight distribution case, a more appropriate aggregation mechanism might therefore take into account the fact that subjects can fall into clusters depending on their strategy use and their favored attribute levels. Our modeling approach points to directions to achieve these nuanced inferences.

### Limitations and Future Directions.

Another important area for future work, building upon and addressing some of the limitations of this study, relates to the attributes’ number of levels. Allowing attributes to have more than two levels not only increases ecological validity but also could result in a different distribution of strategy usage. Indeed, this increase in attribute levels could force respondents to rely even more on heuristic strategies than they do in our experiment, which could possibly be capturing a floor effect. Future work can explore both whether our current experiment can be further condensed and how to generalize the modeling approach to handle multiple attribute levels. Extending the experimental design this way may also shed light on why people choose the strategies they do. Namely, future work should further explore the relationships between strategy use, demographic variables, identity, and individuals’ perceptions of the decision problem (e.g., their interest in the topic). Our own investigations of these relationships were exploratory in nature, and our tentative findings could benefit from more tailored designs and focused analysis in the future. Finally, future work that jointly models choice and process data (e.g., response time) would lead to more precise inferences about the process-level distinctions across strategy classifications, a limitation we only partially overcome through post hoc RT analyses.

All taken together, our results reinforce the psychological and inferential value of studying individual differences in strategy use in multiattribute subjective choice. This work has important implications for how to think about the generalizability of results from conjoint experiments, the psychology of multiattribute political choice, and conjoint experimental design more broadly.

## Materials and Methods

### Strategies.

To concretize the specific problem of strategy classification, we start with the two fundamental considerations (or primitives) that dictate how an individual would choose between subjective multiattribute alternatives: 1) how much a given attribute matters to the person (i.e., attribute weights) and 2) what values or levels of the attribute they favor (i.e., favored levels). We formalize and describe these primitives in more detail in *SI Appendix*, sections A.1 and A.2. Following the decision and political science strategy literature (17, 29–31, 44–50), we then model several well-established strategies that make differential use of these primitives. While our modeling framework is intrinsically flexible in terms of the number of strategies it can include, we choose to focus on a circumscribed set of strategies that span the strategy space as completely as possible while not oversaturating the model, such that we can provide a compact, interpretable, and stable summarization of the distribution of strategy usage. For more information on and justification for our selection of strategies, see *SI Appendix*, sections A.1 and B.1. In our model, subjects can be classified as using any one of four possible strategies: WADD (the rational strategy), Tally, TTB, and Contaminant.

### Cognitive Model.

**Fig. 5.**
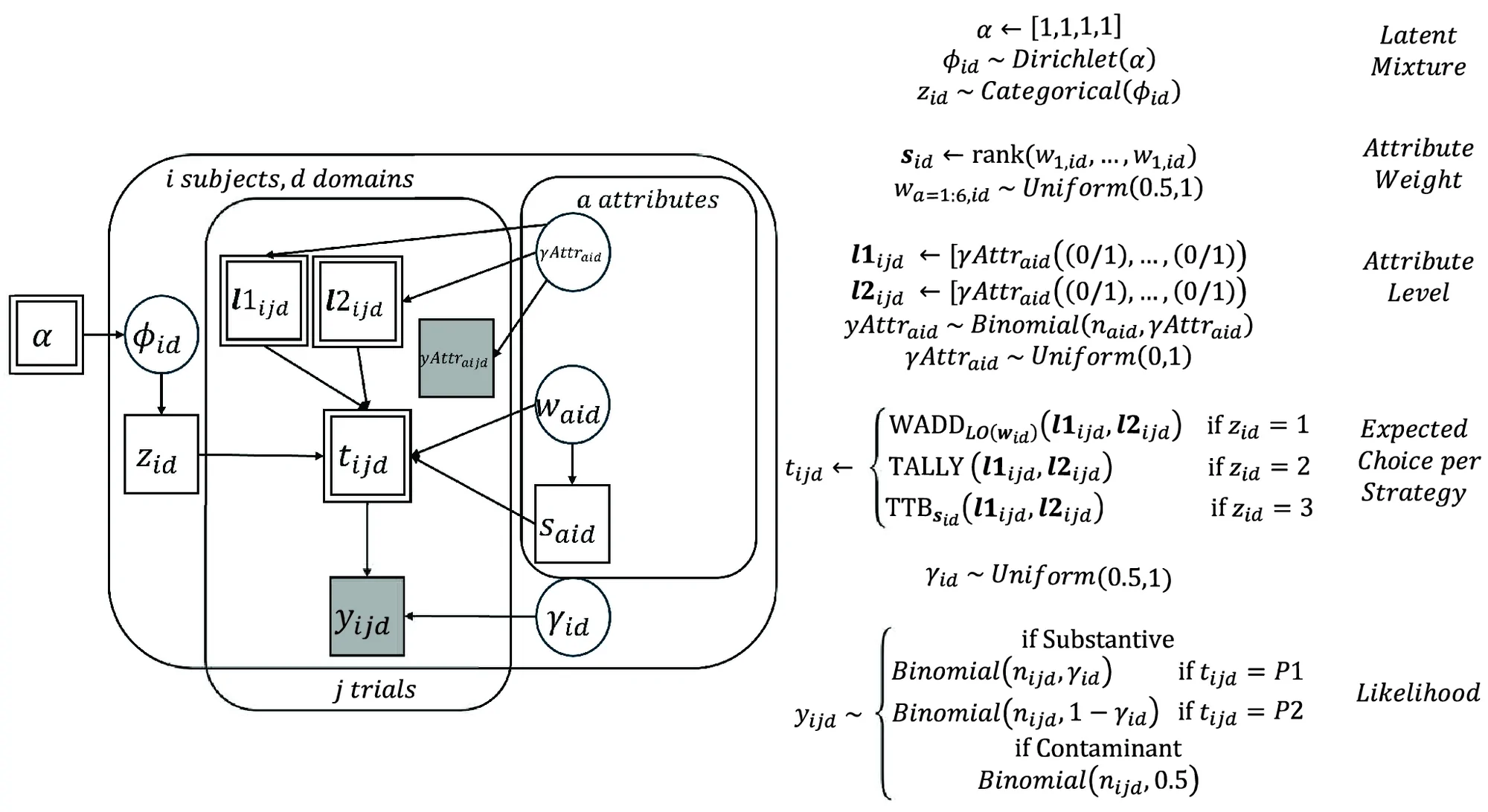
Graphical model. Square nodes are discrete variables and circular are continuous. Shaded nodes are observed while unshaded nodes are inferred. Nodes with double borders are deterministic.

We develop a Bayesian latent-mixture model for classifying individual-level strategy use in subjective multiattribute choice. The model is depicted in Fig. 5 and implemented using JAGS (51) (see *SI Appendix*, section A.3 for more detail). Our model has three key components: 1) the latent-mixture, which is the model-inferred strategy classification (52), 2) attribute weight inference, which captures the model-inferred individual-specific rankings and weightings of all the attributes (e.g., in the Candidate domain: age > prior political experience > gender > etc.), and 3) attribute level value inference, which captures the model-inferred indicator of which attribute level is more favored by any given individual (e.g., for age: the candidate in their 50s is favored over the candidate in their 70s).

In our latent-mixture model, we infer a posterior distribution over all possible strategies for any given subject. That is, a subject can follow any one of the four strategies a priori. We therefore evaluate the posterior probability associated with each possible strategy given the observed choice behavior. Note that in our model, individuals are classified as following the particular strategies whose choice predictions best align with their observed behavior. That is, subjects are not arbitrarily or anomalously classified if the predetermined strategies do not offer an exact fit. This is further captured by how certain the model is in its classification. Taken together, our model extends previous cognitive modeling work by applying latent-mixture classification to the subjective realm, explicitly inferring individual-specific attribute weights and favored levels at the same time as classifying strategy use. *SI Appendix*, *Supplementary Methods*, section A provides more details on the model itself, in addition to the methodological contribution of making this extension to the subjective realm.

### Experimental Design.

Our experiments consider three different political domains that are commonly studied via conjoint experimentation: which immigrants voters would rather admit to the United States (*Immigration*), which Senate primary candidate they would rather vote for (*Candidate*), and which climate policy package they would prefer the United States to adopt (*Climate*). We follow standard and familiar conjoint experiment design in the applied political science literature, though we make some minor design modifications to facilitate inference—increasing the number of trials and restricting all attributes to be binary. Note that previous research in political science has also increased the number of trials per subject to the same extent that we do without evidence of significant respondent fatigue or inattention over time (32, 33, 37), and the inclusion of similarly large numbers of conjoint trials is commonplace in marketing research. Note also that the standard experimental design for investigating strategy use in cognitive science similarly uses binary attributes, though these attribute levels usually indicate the presence or absence of a feature (17). In addition, there has been some previous conjoint research in political science (e.g., ref. 15) that has also employed only binary attributes for methodological purposes.

In Experiment 1 (E1), subjects were randomly assigned to one of two conditions: Condition 1 (Number of subjects (N)=960) where attribute levels were always distinct for each profile (i.e., no tied attribute levels) or Condition 2 (N=1,004) where profiles included 1−3 tied attribute levels. In Experiment 2 (E2), subjects were also randomly assigned to one of two conditions: Condition 1 (N=911) was exactly the same as (and hence a replication of) E1 Condition 1, and Condition 2 (N=887) required subjects to complete rating trials, rating a profile between 1 and 7 indicating support, prior and in addition to choice trials, as is common in applied work (53). In both experiments, all respondents completed three blocks of 30 trials each, where each block corresponded to the three decision domains presented in a randomized order. We then apply our latent-mixture model to these conjoint data in order to classify individuals’ strategy usage and infer individual-level attribute weights and preferences. For more comprehensive details on experimental design, see *SI Appendix*,section A.4.

### Sample.

Subjects for both E1 and E2 were US-based adults recruited via Prolific, with US sample representativeness requirements enforced with respect to age, sex, and political affiliation. Subjects in E1 (N=1,964) participated in our experiment in July 2024, and subjects in E2 (N=1,798) participated in our experiment in January 2025. Further details about the sample, including summary statistics, can be found in *SI Appendix*, *Supplementary Methods*.

### Validation.

We perform a range of validation and quality checks. This includes demonstrating that our model describes the observed data well, that it is reasonably confident in its classifications, and that it is not putting unrealistic structure on the data. We additionally confirm that the AMCEs we estimate with our data in all three domains are broadly consistent with prominent applied conjoint scholarship in the existing political science literature, informally validating the representativeness and behavioral consistency of our sample. In addition, as a validation of our model’s strategy classification, we find close correspondence between our strategy classifications and nonparametric measures, including response times (RT), that capture individual-level behavior theoretically associated with the different strategies.

We also perform a parameter recovery analysis where we simulate data generated from all four strategies using a range of attribute weights, detailed in *SI Appendix*, section C. Our goal was to test the accuracy of strategy classifications and to characterize the sensitivity of WADD classification on qualitatively different sets of attribute weights. Overall, we find that the model recovers TTB and Tally with close to 100% accuracy in both the presence and absence of ties (E1C1, C2) when the synthetic data is noiseless. These values drop as we increase the noise in the simulated choices (89% with 10% noise and 81% with 20% noise). We further find that WADD recovery is contingent on the specific sets of attribute weights used. When attribute weights are compensatory, WADD is correctly classified 93% in the No Ties condition, and 69% in the Ties condition when the synthetic data is noiseless. Again, the accuracy reduces with increasing noise (71.5% with 10% noise and 58.9% with 20% noise). However, when attribute weights are noncompensatory, the WADD generated choices are confidently classified as TTB and similarly when the attribute weights are uniform, the WADD choices are confidently classified as Tally. We take this as evidence that the model is doing what it should be: identifying the strategy with which the choice behavior itself is most consistent. Our recovery is poorer for contaminant data, generated by a coin flip, where we see that a substantial percentage of simulated Contaminant subjects are misclassified as Tally. Despite the inherent limitations in accurately classifying a coin flip, this result shows an important constraint in interpreting Tally and Contaminant classifications in that we are likely underestimating the prevalence of contaminant behavior and overestimating the prevalence of Tally. We elaborate on the implications in *Discussion*.

Below, we present our results on response times, which provide a process-level corroboration of our strategy classification that is independent of choice. *SI Appendix*, section B presents more comprehensive information and results on our other validation and quality checks.

### Response Times.

Response time is the amount of time an individual takes to make their choice. Because this measures the total length of time from trial onset to button click, it captures time taken not only for cognitive processing and decision-making, but also for motor execution of the choice (i.e., moving and clicking) and other “non-decision-related” processes (e.g., regathering attention at a new trial, mind-wandering, etc.). Insofar as the cognitive processing and decision-making vary in their complexity across the different strategies, we should expect differences in the RT distributions across strategies, as well as very specific impacts (and nonimpacts) of moving from the No Ties to the Ties condition. Specifically, a strategy that evaluates less information and/or employs heuristics should result in faster decisions, and the only strategy whose process is complicated by the presence of ties is TTB. Hence, RT data present an opportunity for evaluating strategy classifications at the level of process and separate from choice. To perform our analysis, for each domain we group respondents by their modal strategy classification and then calculate their average RT. We then construct and analyze the respondents’ average RT distribution across the strategies and conditions.

**Fig. 6.**
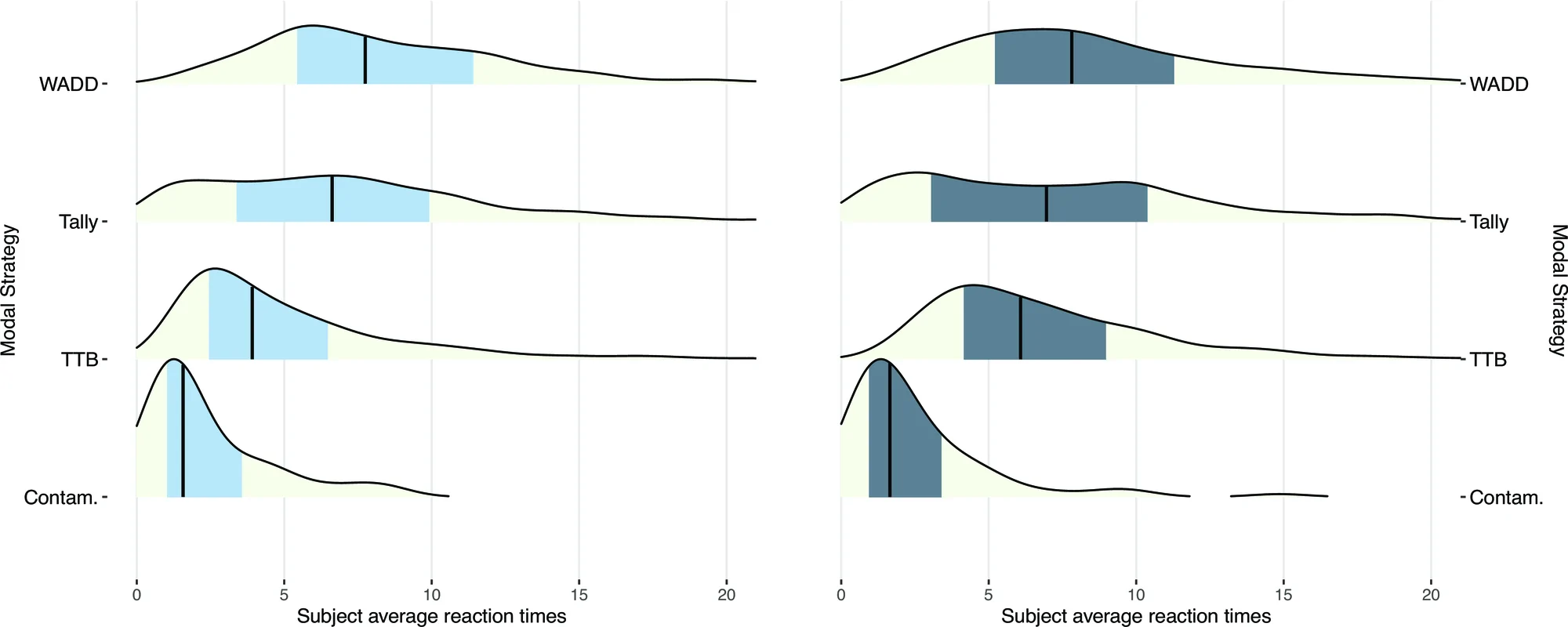
Distribution of mean response times as a function of modal strategy classification and experiment condition. The black bar represents the median RT. Only the TTB response time distributions reject the null of no difference between experiment conditions (KS test P<0.0001). We truncate the x-axis at 20 seconds for visual clarity.

The results, which are shown in Fig. 6 pooling across the domains, provide strong corroborating evidence that our strategy classifications are indeed capturing process-level signal. (*SI Appendix*, *Supplementary Methods* and Fig. S6 shows summary statistics by domain, which highlight consistent patterns.) First, consider the no-ties condition displayed in the *Left* panel. To help visualize, in addition to plotting the densities, we also shade in the middle 50% and insert a vertical line for the median of each density. Looking at any or all these visual cues, we see quite clearly that the RT distributions increasingly move to the left (i.e., respondents take less time) as we go from the highest effort to the lowest effort strategy. RTs are the highest for subjects classified as WADD, consistent with it being the fully rational strategy that requires integrating and weighting information about all attributes. RTs are next highest for Tally, consistent with it also being full-information but involving heuristics. Next is TTB, consistent with it requiring (in the no-ties condition) information on only one attribute. Finally, those classified as contaminants have the lowest and most concentrated RT density, consistent with the RTs for the contaminant strategy being driven simply by the amount of time it takes to execute nonprocessing tasks.

In addition, the RT patterns within-strategy and across conditions are also consistent with the process-level effort that is expected for each strategy under each condition. Specifically, the only strategy where there is meaningful evidence of a change in RT distribution is TTB, consistent with the fact that ties by definition increase the amount of time and effort required for execution of TTB. Further, given that the number of ties present in each trial varies, this should lead to the distribution not only being shifted right but also taking on higher variance. This is precisely what we see and is discernible looking at the full distributions, their shaded regions, and their median lines. We also confirm this formally with a KS test of difference in distributions, which rejects the null hypothesis of no difference between the TTB distributions with P<0.0001. In contrast, for all three other strategies (WADD, Tally, and Contaminant), the RT distributions’ differences across conditions appear minimal, and the KS test fails to reject at conventional levels (all *P*-values > 0.05 even in the absence of multiple comparison correction). This is consistent with the underlying processes involved with these strategies, as WADD and Tally both require full information integration with or without ties, and Contaminants do not meaningfully incorporate any information regardless of ties. To reiterate, the selective effect of ties only on TTB is precisely what the process-level account of our strategies involves. If the presence of ties simply rendered the conjoint tasks more effortful or visually demanding for all respondents, we would expect a slowdown across all strategies, which we do not observe.

### Research Ethics.

Our experiments were approved by the Institutional Review Board at UC Berkeley (Protocol 2024-06-17538). Informed consent was obtained from all participants.

## Supplementary Material

Appendix 01 (PDF)

## Data Availability

Data, replication code, Behavioral (Choice and Response Time) data are available via Harvard Dataverse at https://doi.org/10.7910/DVN/POMLLB (54).

## Footnotes

^*^*SI Appendix*, section B.3 and Table S9 report aggregate information on how much each attribute level was favored (i.e., posterior means and modes at the aggregate level).
