# Supplementary material for: Classifying strategy use in multiattribute subjective choice: Application to conjoint experiments in political science
Source: Proc Natl Acad Sci U S A. 2026 Sep 28;123(40):e2613113123. doi: 10.1073/pnas.2613113123 (PMC13643305; doi:10.1073/pnas.2613113123)
Supplement: Supplementary file 1 [file pnas.2613113123.sapp.pdf]

Supporting Information for

Classifying strategy use in multi-attribute subjective choice: Application to conjoint experiments in political science

Nidhi V. Banavar and Kirk Bansak

Nidhi V. Banavar.
E-mail: nbanavar@berkeley.edu

This PDF file includes:

Supporting text
Figs. S1 to S27
Tables S1 to S9
SI References

Supporting Information Text

A. Supplementary Methods.

A.1. Strategies in the Subjective Domain. What strategies may individuals use and be classified as using they make subjective multi-attribute decisions? Research on strategy use in the decision and social sciences has focused on normative (or rational) frameworks and/or heuristic (or "shortcut") frameworks (1–10).* Standard rational or normative frameworks suppose that individuals evaluate and appropriately weight all information available to them. Heuristics, on the other hand, focus on informational shortcuts, typically on the premise that cognitive capacity constraints make fully rational strategy use implausible. Decades of prolific research has delineated not only a large set of plausible strategies but also various circumstances under which strategies may be differentially deployed (9, 11, 12).

To make our specific problem of strategy classification in subjective choice more concrete, we introduce some necessary conceptual primitives. These are described in more explicit detail in the following paragraphs and formalized in the Methods Section A.2. For the sake of example, let us return to the candidate choice problem: an individual has to choose between two candidate profiles and is provided attribute levels for each candidate (e.g., the age, education, race, gender, etc. of each candidate). What do we need to know about the individual in order to determine what strategy they use?

We need to know (1) how much a given attribute matters to the person and (2) what values or levels of the attribute they favor. In our example, (1) would mean how much the individual cares about age, education, etc. We refer to this as the *attribute weight* $w_{a,id} \in \mathbb{R}$ where a represents the specific attribute, i represents the individual, and d represents the decision domain – political candidate choice in this case. Relatedly, (2) concerns which attribute level is more valued by the individual: older vs. younger in age, more vs. less educated, etc. We refer to this as the *attribute level coding.* Without loss of generality, for binary attributes let this be captured by $l_{a,id} \in \{0, 1\}$, where the favored attribute level is indicated by 1. We can additionally consider the granularity with which we infer attribute weights: (1a) does the individual assign weights to every single attribute or (1b) is a sparser representation, such as ordinal information $s_{a,id}$, enough?

Taking these primitives together and following the decision and political science strategy literature (1–3, 6, 7, 13), we choose to model three well-established strategies that make differential use of the primitives in ways that map onto distinct ways of making decisions: Weighted Additive (uses both $w_{a,id}$ and $l_{a,id}$), Tally (uses only $l_{a,id}$), and Take The Best (uses a subset of $s_{a,id}$ and $l_{a,id}$). In addition, we model a Contaminant strategy that allows the presence of errant subjects who may not complete the task as instructed, as can particularly be the case with online experiments. These strategies are described in more detail below.As the non-contaminant strategies have been developed, applied, and most richly studied in the *objective* decision domain (i.e., where there is a correct answer), we elaborate further below on the concept of attribute weights in the subjective domain and introduce the concept of attribute discriminability.†

The *Attribute Weight*, $w_{a,id}$, represents how informative an attribute a is for the *general* decision problem, i.e. how important any given attribute is for an individual.‡ In the traditional context of objective decisions, these weights are defined as the probability that an attribute indicates the correct response. As our motivation and methods are focused on subjective choice, we retain the core idea that these weights represent informativeness/importance but without claims about accuracy. Consequently, we also allow attribute weights to vary across individuals: what is informative/important to one person may not be so for another, independent of any theoretically informed optimal assignments of weights.

These $w_{a,id}$ therefore provide a weighted, subjective ordering of attributes.§ As previewed earlier, this also requires that we allow subjects to have variability in which attribute *level* is informative (i.e., favored) to them, $l_{a,id}$. For instance, one subject may find profiles with older candidates to be more favorable while another may have the converse evaluation. Attribute weights can also determine the order in which attributes are evaluated, typically from the attribute with the largest weight to the smallest.

Attribute Discriminability, on the other hand, represents how informative an attribute is for the *specific* trial j. That is, despite knowing which level the individual favors and how much they weigh a given attribute, do both profiles have different attribute levels and therefore provide different pieces of information? Attribute discriminability plays an important role when profiles contain ties: if an attribute has a large weight (e.g., age) but both profiles have the same attribute level (e.g., both candidates are in their 50s), age is not discriminably informative on that trial.

Attribute weights and discriminability take on differing degrees of importance depending on the strategy an individual uses. In our model, subjects can be classified as using any one of four possible strategies, ordered in the list below by decreasing computational requirements:

1. **Weighted Additive** (WADD), where an individual allocates a weight to each attribute, $w_{a,id}$, and then sum together the weights for informative attributes (i.e., preferred attribute levels, $l_{a,id}$), ultimately choosing the profile with the highest numerical value.

2. **Tally**, where an individual counts the total number of informative attributes, $l_{a,id}$, each profile contains and choose the profile with the greater number of informative attributes.

* We note that it is tempting to dichotomize and contrast these, but much, especially recent, scholarship in this area strikes more of a middle ground.

† In the original cognitive science literature, attributes are instead usually called *cues*.

‡ This is traditionally referred to as *validity* in the cognitive science literature.

§ Attributes can have the same weights. The tie-breaker becomes presentation order: the attribute that appears visually higher up in the conjoint table will take on the higher rank.

3. **Take The Best** (TTB), where an individual picks the profile containing their most important and discriminable attribute.Recall that $s_{a,id}$ is the attribute rank, where $s_{a,id} = 1$ is the most important. In a scenario where the profiles do not share any common attribute levels (e.g., the No Ties condition in Experiment 1 & 2, described later), this always means choosing the profile with the informative attribute level for the attribute with the highest weight (search depth = 1). When there are ties (e.g., the Ties condition in Experiment 1), this is not necessarily the case as both profiles may have the same attribute levels for some number of the highest weighted attributes (search depth ≥ 1).

4. **Contaminant**, where an individual's behavior is best explained by having a 50-50 chance of picking either profile.

While these strategies are cognitively distinct, from a strictly mathematical perspective, the two heuristic substantive strategies could be seen as variants of the WADD strategy. The Tally strategy, for example, would be equivalent to a WADD strategy with uniform weight and TTB would be equivalent to a WADD where one attribute (or more in the case of ties) has so much weight compared to the other attributes such that it cannot be compensated for. It is theoretically possible, therefore, to just fit a model only allowing for a WADD strategy and recover reasonable performance (see Tables S2,S4). However, such a model would be misleading in its interpretation that individuals can indeed classified as rational evaluators of all information provided to them. We aim to explicitly test whether this is the case by including strategies that deviate from this approach in addition to the rational strategy. The TTB and Tally algorithms are distinct enough from WADD in their expectations over the attribute weights that the model is able to distinguish between them by comparing the distribution of attribute weights: overwhelming weight on one attribute relative to the others is classified as TTB, roughly uniform non-zero weight over all attributes as Tally, and distributions in between are classified as WADD, Figure S18. Psychologically, and from a process perspective, as we have articulated, these three strategies have distinct interpretations and have been empirically validated extensively, and hence should be modeled as distinct. For a detailed exploration of how different attribute weights shape strategy classification, see Supplementary Methods, Section C "Parameter Recovery." See also the RT section in the Materials and Methods section of the main text.

We reiterate that we choose these four strategies to span the strategy space as completely as possible while not oversaturating the model, such that we can provide a compact, interpretable, and stable summarization of the distribution of strategy classification. The selection and number of strategies was determined not only by this theoretical motivation but also after piloting different combinations of strategies: adding more strategies did not add substantive explanatory gain in this context as, due to the binary nature and number of attributes, the choice predictions made by additional strategies overlapped with the choice predictions made by our three valid strategies. That being said, our modeling framework is intrinsically flexible in terms of the number of strategies it can include. If our experimental or modeling approach required it, we can freely include more or fewer strategies.

Finally, we emphasize that individuals are classified as following the particular strategy whose choice predictions best align with their observed behavior. Subjects are not arbitrarily or anomalously classified if one of our predetermined strategies is not an exact fit. This is further captured by how certain the model is in its classification. Subjects undeniably following a specific strategy have all the posterior weight on that one strategy while subjects who may have more noisy data, or may be following a mix of strategies or a strategy not specified by us, will have posterior weight distributed across the possible strategy classifications, resulting in lower model classification certainty. Equipped with these strategies, we build, validate, and apply a cognitive model to study strategy use in multi-attribute subjective choice, as applied to the political decision-making domains captured in our conjoint experiments.

A.2. Cognitive Model.

Latent Mixture The latent mixture approach allows for every subject's behavior to be produced by one of two broad classes of behavior: substantive (i.e., following a strategy/decision rule with an inferrable degree of fidelity) vs. contaminant (i.e., behavior more consistent with guessing). This results in a likelihood function that is a weighted mixture of the likelihood from behavior generated by a substantive strategy and by a contaminant strategy (14). While there has been some scholarship that has treated behavioral inconsistency in surveys as measurement error that needs to be corrected (15), it is well-established that there are stochastic elements inherent in human choice processes (16), with which a Bayesian model is theoretically consistent.

We allow each subject i to be classified as using different strategies per decision domain d. The strategy classification parameter itself, z_{id}, is distributed as a Categorical variable with a conjugate Dirichlet distributed parameter vector ϕ_{id}. This represents an individual's base rates over possible strategies, again varying for each domain d. We set the concentration hyperparameter α to be uninformative.

$$
\begin{aligned}
z_{id} &\sim Categorical(\phi_{id}) \\
\phi_{id} &\sim Dirichlet(\alpha) \\
\alpha &\leftarrow [1, 1, 1, 1]
\end{aligned}
$$

While we did our best to ensure that the strategies considered span the space of plausible strategies, it is possible that a subject followed a strategy that we did not explicitly account for. In such a case, a subject would be classified by the included strategy that best described their behavior. In other words, the model captures what their behavior is most consistent with, and hence is still useful even though it is not feasible to include all possible strategies. We also clarify that the actual number of

strategies modeled is determined by the researcher: it is entirely possible to incorporate a greater or fewer number of strategies, though we urge caution with regards to identifiability if more strategies are included.

$$t_{ijd} \leftarrow \begin{cases} \text{WADD}\left(\text{Log Odds}(\mathbf{w}_{id}), (\ell 1_{ijd}, \ell 2_{ijd})\right) & \text{if } z_{id} = 1 \\ \text{Tally}\left(\ell 1_{ijd}, \ell 2_{ijd}\right) & \text{if } z_{id} = 2 \\ \text{TTB}\left(\mathbf{s}_{id}, (\ell 1_{ijd}, \ell 2_{ijd})\right) & \text{if } z_{id} = 3 \end{cases}$$

For each trial j, then, the choices each subject should make if they were to follow each substantive strategy, (t_{ijd}), are determined by input from a input a 2-dimensional vector containing attribute level coding $(\ell 1_{ijd}, \ell 2_{ijd})$ and any other strategy-specific information. The two elements of $(\ell 1_{ijd}, \ell 2_{ijd})$ are themselves vectors containing attribute value information for each choice option (i.e, does the profile contain the attribute level that the individual cares about, described in more detail in Section A.2). For example, suppose a subject has to evaluate two political candidates. She needs to be able to represent all the attribute information that comprises each candidate *and* whether the attribute value a candidate holds is favored to her. That is, say our voter has a preference for younger political candidates and let Candidate A be in his 70s and Candidate B in 50s. In this case, Candidate B has the favorable age attribute level and Candidate A does not. Our voter has to do the same for all 6 attributes presented for a given profile, thus forming these $\ell 1_{ijd}, \ell 2_{ijd}$ vectors. These vectors are then evaluated as specified by each algorithm. If a person only considers these vectors but evaluates them completely, they are classified as following Tally. If, instead, a person not only considers these vectors but also weights each attribute $(\mathbf{w}_{id})$, they are classified as following the WADD strategy. If a person considers a much sparser representation and only requires attribute ranking information $(\mathbf{s}_{id})$ – and that they only consider the attribute with the highest weight – then they are classified as following TTB. As described in Section A.1, subjects who are classified as contaminant are subjects whose choices are best described by randomly choosing either profile.

Attribute weight inference In the objective decision domain, attribute weights can typically be calculated and do not vary across individuals. This, however, is not the case in the subjective decision domain: we must explicitly adopt a modeling approach that allows for attribute weights to possibly vary across individuals and decision domains. As with strategy classification, we implement an uninformative uniform prior over the attribute weights,$\mathbf{w}_{a,id}$, and their respective rank orderings, $\mathbf{s}_{a,id}$, for each attribute a, individual i, and decision domain d. We also note that attributes weights are drawn independently of each other for consistency with how attribute weights are calculated in the objective domain. For the WADD strategy, the only one that uses the numerical values of the attribute weights $\mathbf{w}_{a,id}$, we do not model the raw attribute weights and instead model the log-odds of the weights, as would be normatively expected (17). We therefore restrict the uniform prior distribution of weights to be between 0.5 and 1: a weight of 0.5 has a log-odds of 0 and therefore is uninformative while a weight of 1 is the most informative. Recall again that informativeness is with respect *to the individual* and not a domain-general concept. Assuming six attributes (as in our experiments described later), though without loss of generality, we infer attribute weights $w_{1,id}, ..., w_{6,id}$ for each subject i per decision domain d, where each attribute weight is independently drawn from this Uniform$(0.5, 1)$ prior distribution. We form the search order $s_{1,id}, ..., s_{6,id}$ by rank ordering draws from the distributions of each of the weights.

That is, for subject i, decision domain d, and each attribute a $(a = 1, .., 6)$:

$$\mathbf{s}_{id} \leftarrow \text{rank}(w_{1,id}, .., w_{6,id})$$
$$\mathbf{w}_{a=1:6,id} \sim Uniform(0.5, 1)$$

where the distribution of $\mathbf{w}$ here represents the prior.

Prior Selection. We tested several different parametrizations of the attribute weights $w_{a,id}$. Namely, we additionally considered:

$$\mathbf{w}_{a=1:6,id} \sim Normal(0, 1)T(0, 1)$$
$$\mathbf{w}_{a=1:6,id} \sim Beta(1, 1)$$
$$\mathbf{w}_{a=1:6,id} \sim Normal(0.5, 1)T(0.5, 1)$$

From an interpretability perspective, given that we parameterize weights as log-odds, we ideally wanted a prior with a lower bound of 0.5 as $\log(\frac{0.5}{0.5}) = 0$. As our strategy classification results did not change substantially as a function of prior specification, we ultimately selected the prior that had the clearest psychological and statistical interpretability: $Uniform(0.5, 1)$.

Attribute level inference In order to complete all the above pieces, however, we need a final piece of information: which level for each attribute does a given subject favor? For example, when considering the political record of a potential US Senate candidate, does a given subject favor candidates who have prior political experience or no prior political experience? Just as with the attribute weights themselves, which attribute levels are favored may be reasonably expected to vary across individuals. We therefore include a component in the model that indexes attribute level coding for each attribute, for each subject, in each decision domain.

We do this by first randomly assigning the attribute levels for a given attribute to be $x = 1$ and $y = 0$, where 1 is informative and 0 is uninformative (to the individual). This step is performed outside the model. Then, inside the model, we allow for each

of these attribute levels to flip, allowing the model to determine which attribute level should be considered the most informative according to the data, inferred by $\gamma Attr_{aid}$. That is, let *Yes, Prior Political Office* $= 1$, for example, outside the model, representing it being preferred for the given subject. We leverage the fact that attribute weights are drawn independently of each other to test whether the initial assignment of *Yes, Prior Political Office* $= 1$ is a better fit to the data or instead *No, Prior Political Office* $= 1$ is the more appropriate assignment. In other words, this sub-stream of the model effectively assumes a TTB strategy where each attribute is the only attribute that matters for that decision domain. Given this, the attribute level that is better fit by the relevant data is selected. As the conjoint profiles themselves are randomly generated for each trial, the model therefore constructs trial-level $\boldsymbol{l}$ vectors of 0s and 1s, representing whether each given profile contained informative or uninformative attribute *levels* as previously inferred. Concretely, if $\gamma Attr_{\text{Prior Office},id} < 0.5$, for when *Yes, Prior Political Office* $= 1$ initially, then the trial level attribute coding in the respective $\boldsymbol{l}$ vectors would flip such that *No, Prior Political Office* $= 1$. If instead $\gamma Attr_{\text{Prior Office}id} > 0.5$, then no change would be made to the relevant $\boldsymbol{l}$ vectors.

$$\ell 1_{ijd} \leftarrow [\gamma Attr_{aid}((0/1), ..., (0/1))]$$
$$\ell 2_{ijd} \leftarrow [\gamma Attr_{aid}(0/1), ..., (0/1))]$$
$$yAttr_{aijd} \sim Binomial(n_{ijd}, \gamma Attr_{aid})$$
$$\gamma Attr_{aid} \sim Uniform(0, 1)$$

where the distribution of $\gamma Attr$ here represents the prior.

Putting it together as a Bayesian cognitive model We form our Bayesian cognitive model by putting all these pieces together with a Binomial likelihood for the data, Figure 5, Main Text. Recall that in Bayesian inference, we begin with prior beliefs (in our case, “uninformative” beliefs), which are then updated by the what is observed (the likelihood) to form our posterior beliefs.

The y_{ijd} observed choice is the number of “successes” (i.e., number of times the profile on the right is chosen) and n_{ijd} is the number of times a subject is presented with a specific combination of conjoint profiles. What is inferred by γ_{id}, then, is how well the observed choice(s) align with the model-selected strategy’s *predicted choice* (i.e., the number of times a person classified as following TTB would select the profile on the right given the specific decision problem/profiles).

$$y_{ijd} \sim \begin{cases} \quad \text{if Substantive} \\ Binomial(n_{ijd}, \gamma_{id}) \text{ if } t_{ijd} = P1 \\ Binomial(n_{ijd}, 1 - \gamma_{id}) \text{ if } t_{ijd} = P2 \\ \quad \text{if Contaminant} \\ Binomial(n_{ijd}, 0.5) \end{cases}$$
$$\gamma_{id} \sim Unif(0.5, 1)$$

where the distribution of γ here represents the prior.

The graphical model in Figure 5, Main Text shows how the pieces work together simultaneously: the inference on attribute level importance together with the attribute weights/ranks determine the choices that a subject would make if they followed a particular substantive strategy, t_{ijd}. The latent inference, z_{id} determines which strategy best describes the subject’s actual behavior by comparing the outputs with the observed data y_{ijd}. The parameters ϕ_{id} and γ_{id} therefore have clear psychological interpretations: ϕ_{id} represents the base rate for each individual over all possible strategy types and γ_{id} represents the fidelity with which a strategy is executed if a person is following a substantive strategy, or 0.5 if a person’s best fit strategy is selecting either profile with equal probability.

Perhaps the fundamental reason Bayesian cognitive models are commonly used by researchers either as some approximation for human cognition or as a useful statistical framework to model the cognitive processes of interest is because of the inherently stochastic nature of human behavior. In a modeling exercise such as the one we conduct in this study, where we model strategies that are deterministic algorithms in a psychologically interpretable fashion, scrutiny is demanded about how we incorporate uncertainty. We have built in probability at two broad levels: (1) the likelihood via both the rate parameter γ_{id} and the latent-mixture z_{id} and (2) attribute inference. Probability at the level of strategy execution (1) is typically how such uncertainty is incorporated. If this is the only point at which uncertainty is incorporated then we expressly assume each individual has perfect access to their internal representations of attributes and weights and that the weights assigned to the attributes themselves can be well explained by a point estimate. In the case of inferring strategy in the objective domain, these latter assumptions, while still stringent, pose less of a concern. We explicitly include a second level of uncertainty at the level of the attributes themselves in our model. Indeed we expect individuals’ internal representations of these attributes to not only be noisy, depending on the attributes, but also that an individual may not have perfect access to this information. Future work can expand the model to test whether the fidelity of strategy use is a function of inherently less precise internal representations or imperfect access to these representations. This will be particularly important when considering attributes with more than two levels.

A.3. Model Implementation. We implement our Bayesian models using JAGS (18) in R. JAGS performs MCMC using the Gibbs sampling algorithm. We run models for 10,000 iterations with a burn-in of 8,000, thinning posterior samples by a factor of 5. We run 5 chains for each model. Reported results are from models that have converged (i.e., showing no autocorrelation the posterior samples and having reasonable values of the Gelman-Rubin statistic, *Rhat*).

A.4. Experiments. We model data from two online experiments (Experiment 1: $N = 1964$, Experiment 2: $N = 1798$) where subjects each made a total of 90 two-alternative forced choice decisions between two profiles over three blocks. Subjects were recruited online via the survey platform Prolific, which provided a sample that was representative of the U.S. adult population with respect to age, gender, and political party affiliation. Every trial included a conjoint table which listed the six attributes for each decision domain and their randomly assigned levels for the two choice options, e.g., Figure S1.

In Experiment 1 (E1), subjects were randomly assigned to one of two conditions: Condition 1 ($N = 960$) where attribute levels were distinct for each profile (i.e., no tied attribute levels) or Condition 2 ($N = 1004$) where they saw profiles with 1, 2, or 3 tied attribute levels determined by a draw from a uniform distribution. We included these two conditions for two reasons. First, both approaches – restricting ties and allowing for ties – are employed in applied research (though allowing for ties is more common in political science). Second, we also wanted to investigate the degree to which allowing for or restricting ties (i.e., manipulating the stimulus complexity of the conjoint) affected strategy usage. In both conditions, subjects were asked to choose their preferred profile in each pair (i.e., choice trials).

We then collected a second wave of data (recruiting new subjects) several months later with two objectives. The first was to replicate our findings from E1, particularly the No Ties condition, to validate the stability of our modeling framework and inferences. The second objective was to investigate the effects on strategy use of a new modification to the experiment, specifically an additional conjoint design element that is frequently implemented in applied research. In Experiment 2 (E2), therefore, subjects were also randomly assigned to one of two conditions: Condition 1 ($N = 911$), exactly the same as in Experiment 1 Condition 1, or Condition 2 ($N = 887$) where subjects completed rating trials in addition to choice trials, as is common in applied work (19). A rating trial involved a subject assigning a number between 1 and 7 indicating how much they supported a given profile. In this condition, subjects therefore made three decisions in the same sequence for every pair of conjoint profiles presented: rating profile A, rating profile B, and a choice indicating their preferred profile. Subjects' ratings for profile A and profile B were additionally displayed on the choice trial. The decision prompt, however, was the same for both conditions and subjects were not explicitly told to make a choice consistent with their ratings. Note that our goal was not to analyze the rating data itself but rather to evaluate whether prompting subjects to provide ratings affected their subsequent strategy usage when engaging in their choice trials. For instance, one might expect that the act of rating profiles individually on a more fine-grained scale might subsequently lead to the usage of a more full-information strategy when making the choice itself.

In both experiments, subjects were asked to make decisions in three political domains for each block of 30 trials each: *Immigration* ("Which of the following two immigrant profiles would you prefer to admit to the US?"), *Candidate* ("Which of the following two candidates for US Senator would you vote for, assuming both from your preferred party?"), and *Climate* policy ("Which of the following two global climate agreements would you prefer the US adopt?"). The order in which subjects encountered each domain was randomized across all participants. We chose the Immigration and Candidate domains as they are the two most widely studied in political science using the conjoint format (20–25). Inferring attitudes about climate policy using conjoint surveys has also been subject to extensive recent research (26–28).

Table S1 displays the attributes and levels we employed in each domain. All attributes were binary (two levels only). We determined the attributes for the Immigration and Candidate domains by surveying the literature to find frequently-used attributes that could reasonably be reduced to two attribute levels. Similar to other previous research (19), we explicitly do not include political party as an attribute for the Candidate domain given the dominant role the partisan identity of potential candidates often plays when voters decide for whom to vote (1, 7, 29). We determined attributes for the Climate domain by selecting a subset from (26). For each domain and each subject, we randomized the order in which attributes were displayed for each subject but held this order constant within block. We also randomized the order in which the domains were presented for all subjects.

For both experiments, we further collected information about how meaningful these decision problems were to each individual by asking subjects to rate each domain on a scale of $0 - 10$ in terms (1) their interest in the domain, (2) how informed they were about the domain, (3) how important they thought it was, and (4) how complicated they thought it was, Figure S2. Subjects completed these trials twice for each decision domain – before and after each conjoint trial block.

Analyses reported using these ratings uses the average of the pre- and post-block ratings. As individuals are thought to adaptively deploy strategies in response to complex decision environments (30), we collected these data to enable subsequent analyses that may shed light on why someone uses the strategy they do for each decision domain.[¶] We selected these four measures of an individual's relation to the decision domain as they have been theorized and demonstrated to influence strategy use (30, 32, 33). For example, people with more knowledge about a decision domain (i.e., more "expert") may be less likely to be classified as using the rational strategy due to their ability to focus on the most relevant attributes. Conversely, people with more interest (i.e., rate the domain as more interesting or important) may be intrinsically more motivated to use the more exhaustive strategy. Subjects provided these ratings twice: before and after each decision block. After each decision block, we

[¶] The complexity of experimental design or structure is also demonstrated to impact strategy (30, 31). However, we treat the three decision domains themselves as comparably complex from this perspective as all have the same number of choice options, attributes, and attribute levels. Further, the attribute levels themselves are a mix of the quantitative and qualitative for all domains. Note that we do manipulate structural complexity across the several experimental *conditions* as described above: the presence/absence of ties and the presence/absence of rating trials in addition to choice trials.

also asked subjects to indicate how much each attribute figured into their decision process by assigning a number between 0 and 10 for each attribute, Figure S3.

We further collected demographic information from our respondents for both experiments. This includes self-reported party identification (E1: 43.4% Democratic, 35% Republican, 21.7% Neither; E2: Democratic 43%, Republican 35.2%, Neither 21.8%). Here, we specifically consider whether certain demographic characteristics may modulate strategy deployment. We focused here on demographics that may feasibly play a role: partisanship rating (0 Left to 10 Right; E1 & E2 median = 5), age E1 & E2 median = 48), education (8 levels; E1 & E2 modal education is Bachelors), and ability to vote (0 or 1; with 99% having vote eligibility, E1 & E2). We also consider race (19 – 24 distinct self reported categories for both experiments, modal White/Caucasian with 77%) and gender (approximately 50% − 48% − 2% female−male−non-binary) for completeness.

While we allow our model to explicitly and independently infer attribute weights (Section A.2), these user-provided ratings serve as an important comparison point. Finally, we collected the following demographic information from each subject: age, education, employment status, ethnicity (Hispanic/Latino), gender, income, left-right political ideology (number 0 − 10), political party membership, race, and vote eligibility.

We excluded a total of 47 subjects from our initial data collection (E1: 22, E2: 25) for failing to pass either of our quality checks. A subject was excluded if they (1) did not pass both attention checks and/or (2) had a mean response time lower than 0.3 seconds, impossible for humans to do if behaving consistently with the task.

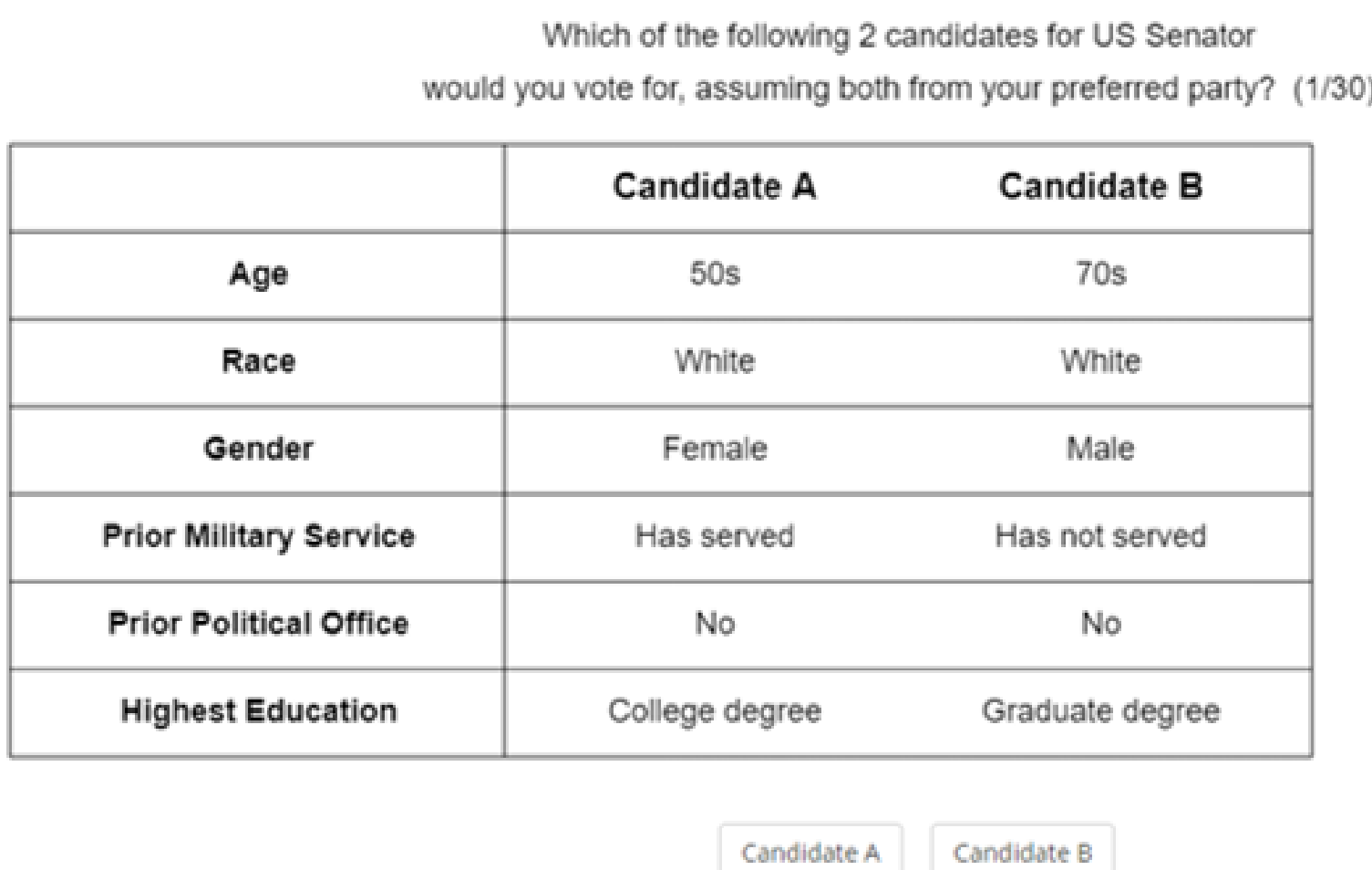
Which of the following 2 candidates for US Senator
would you vote for, assuming both from your preferred party? (1/30)

	Candidate A	Candidate B
Age	50s	70s
Race	White	White
Gender	Female	Male
Prior Military Service	Has served	Has not served
Prior Political Office	No	No
Highest Education	College degree	Graduate degree

Candidate A Candidate B

Fig. S1. Example trial for political candidate in US senate primary Subjects have an unlimited amount of time to indicate their preference for either Candidate A or Candidate B via mouseclick. All attributes are binary. The order in which attributes are presented are randomized for each subject but held constant within each block. This example subject has been assigned to Condition 2 in Experiment 1, where attribute level ties are allowed (both candidates are White and both Candidates A and B did not previously hold political office).

Immigration Attributes	Levels
Age	Above retirement, Below retirement
English Proficiency Level	Basic or none, Intermediate
Gender	Female, Male
Highest Education	Below college, College or above
Immigrant Type	Asylum Seeker, Economic migrant
Region of Origin	Central America, Sub-Saharan Africa
**Candidate Attributes**	**Levels**
Age	50s, 70s
Gender	Female, Male
Highest Education	College degree, Graduate degree
Prior Military Service	No, Yes
Prior Political Office	No, Yes
Race	Black, White
**Climate Attributes**	**Levels**
Avg Monthly Household Cost	$53, $160
Emissions Represented	40%, 80%
International Cost Distribution	Only rich countries pay, Prop. to current emissions
Monitoring by	Government, Independent commission
Participating countries	80, 160
Monthly household sanction	None, Yes($32)

Table S1. Attributes and levels for each decision domain/block.

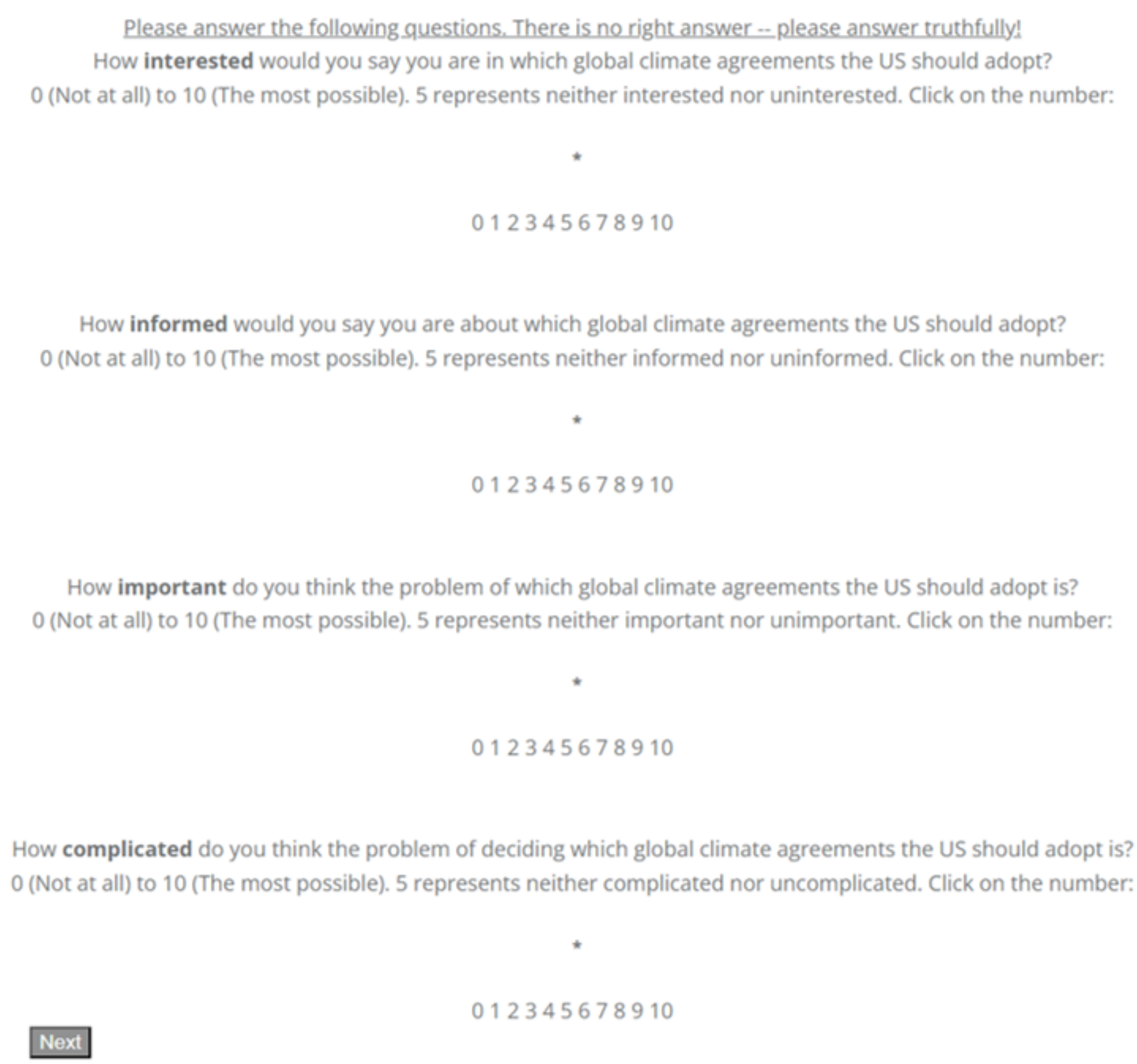
Please answer the following questions. There is no right answer -- please answer truthfully!

How **interested** would you say you are in which global climate agreements the US should adopt?

0 (Not at all) to 10 (The most possible). 5 represents neither interested nor uninterested. Click on the number:

*

0 1 2 3 4 5 6 7 8 9 10

How **informed** would you say you are about which global climate agreements the US should adopt?

0 (Not at all) to 10 (The most possible). 5 represents neither informed nor uninformed. Click on the number:

*

0 1 2 3 4 5 6 7 8 9 10

How **important** do you think the problem of which global climate agreements the US should adopt is?

0 (Not at all) to 10 (The most possible). 5 represents neither important nor unimportant. Click on the number:

*

0 1 2 3 4 5 6 7 8 9 10

How **complicated** do you think the problem of deciding which global climate agreements the US should adopt is?

0 (Not at all) to 10 (The most possible). 5 represents neither complicated nor uncomplicated. Click on the number:

*

0 1 2 3 4 5 6 7 8 9 10

Next

Fig. S2. Pre-block meaningfulness ratings example. Subjects assign numerical values to how interested, informed, important, and complex the decision domain is to them before making their decisions. Subjects also provide the same information *after* they complete the decision block, responding instead to the prompt "Now that you have made your decisions, please answer the following questions again. It is OK if your answers are different from before." Everything else is the same as shown in the figure.

A.5. Statistical Testing. All statistical tests reported use two-tailed tests.

Figure 4 (Main Text), S18, S19, S22, S23, S24: We extract the posterior mean attribute weight for each subject for each attribute and condition. We then visualize the distributions of these posterior means by constructing density plots. In Figures S18 and S23, we subset subjects by using their modally classified strategy. In Figures S19 and S24, we subset subjects by using

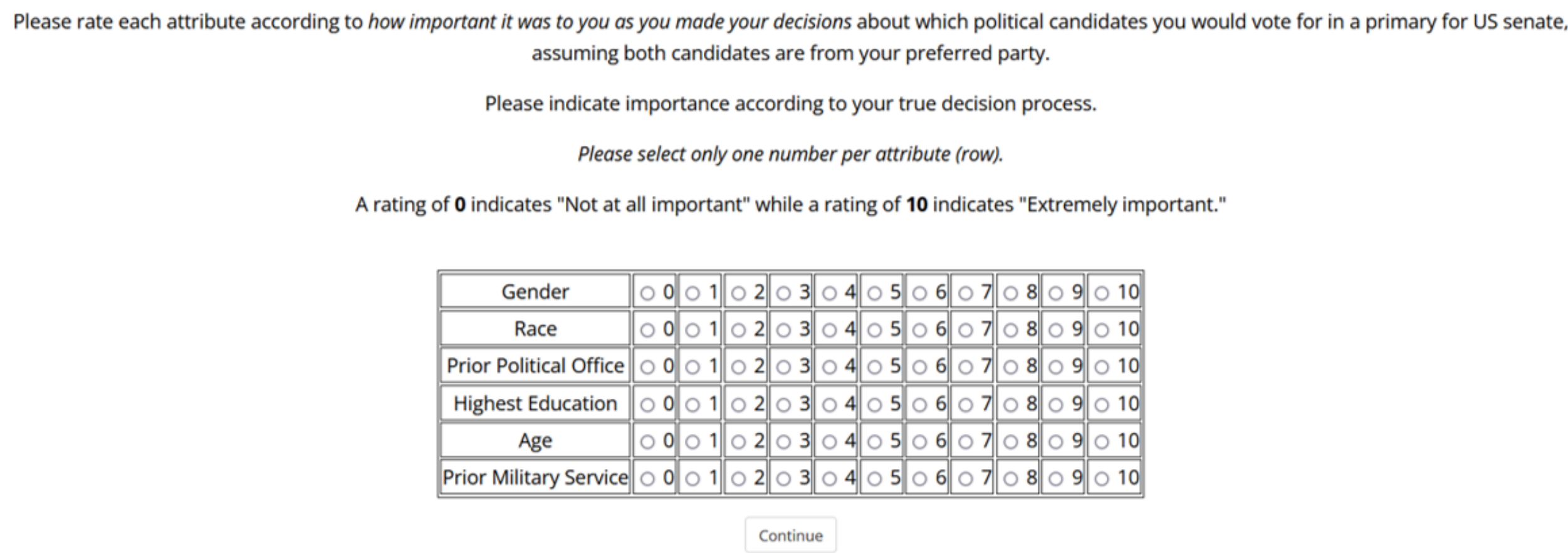
Please rate each attribute according to *how important it was to you as you made your decisions* about which political candidates you would vote for in a primary for US senate, assuming both candidates are from your preferred party.

Please indicate importance according to your true decision process.

Please select only one number per attribute (row).

A rating of **0** indicates "Not at all important" while a rating of **10** indicates "Extremely important."

Gender	○ 0	○ 1	○ 2	○ 3	○ 4	○ 5	○ 6	○ 7	○ 8	○ 9	○ 10
Race	○ 0	○ 1	○ 2	○ 3	○ 4	○ 5	○ 6	○ 7	○ 8	○ 9	○ 10
Prior Political Office	○ 0	○ 1	○ 2	○ 3	○ 4	○ 5	○ 6	○ 7	○ 8	○ 9	○ 10
Highest Education	○ 0	○ 1	○ 2	○ 3	○ 4	○ 5	○ 6	○ 7	○ 8	○ 9	○ 10
Age	○ 0	○ 1	○ 2	○ 3	○ 4	○ 5	○ 6	○ 7	○ 8	○ 9	○ 10
Prior Military Service	○ 0	○ 1	○ 2	○ 3	○ 4	○ 5	○ 6	○ 7	○ 8	○ 9	○ 10

Continue

Fig. S3. Post-block attribute weighting example. Subjects assign numerical values to how important a role each attribute played in their decision-making process. This is our self-reported measure of attribute weight.

their modally inferred favored attribute level.

Figures 1 and 3 (Main Text): To perform this estimation, we randomly sample classifications from the posterior 1000 times for each subject and compute the proportions of strategy classification across subjects for each of the 1000 iterations (i.e., proportion TTB, WADD, for iterations 1, 2, etc.). The point estimates denoted by the height of the bars in the figure are computed as the mean of the proportions classified across the 1000 iterations. The intervals shown in the figure are 95% credible intervals that are constructed using the percentiles of the iterations.

Figures 2 (Main Text) and S25: We take strategy classification as a fixed characteristic by computing the modally classified strategy for each subject in each domain. We then perform a Bayesian t-test, implemented in JASP (34), using the default uninformative prior distribution *Cauchy*(0,0.707) to evaluate the statistical significance of experimental design on the number of strategies used by an individual.

All Bayesian t-tests and Bayes factors reported are implemented in JASP (34).

We use the non-parametric two-tailed Wilcoxon Rank Sign test to perform analyses detailed in the SI Section D.4, comparing subject-provided Meaningfulness ratings across experimental conditions and domains, as these ratings were not Normally distributed.

AMCE inference We follow standard procedures for inferring both average marginal component effects (AMCEs) and individual-level marginal component effects (IMCEs). For implementation, we fit ordinary least squares regressions using the `cjoint` package (35).

B. Model Validation.

B.1. Model Adequacy and Goodness of Fit. To assess how well the model describes the data, also known as descriptive adequacy, we consider posterior predictive agreement: how well the model-predicted choices agree with observed data. We find correspondence in both conditions: 92.2% agreement with no ties (E1 Condition 1) and 93.4% agreement with ties (E1 Condition 2), Figure S4. These percentages represent the Posterior Predictive Adequacy (PPA), a measure of how well the model describes the observed data. It is calculated by simulating choices using the posterior values inferred when fitting the data. The PPA is therefore a within-sample metric of goodness of fit. We further find that the model does substantially better at predicting choices in the candidate domain compared to the immigration and climate domains in both conditions. These results provide a high-level signal on the quality of the model's performance in all three substantive components: the model is able to determine which attribute level matters to which subject, infer which attributes matter more to each subject and by how much, and appropriately combine this information to classify the best-fit strategy. If this was not the case, the model would not be able to describe the observed data at such a high standard.

To further evaluate the goodness of fit of the model, we fit different subsets of the model presented. We fit the following additional models: 1) 2 Strategy TTB and Contaminant, 2) 2 Strategy WADD and Contaminant, 3) 3 Strategy Tally, TTB, and Contaminant, and 4) 3 Strategy WADD, TTB, and Contaminant. We present in Tables S2 and S3 (E1C1) and Tables S4 and S5 (E1C2) the results of two different model comparison metrics: the Deviance Information Criterion (DIC, a Bayesian measure of predictive accuracy) and the Watanabe-Akaike Information Criterion (WAIC, asymptotically equivalent to cross-validation loss). For both of these metrics, the lower the value the better. The results in Tables S2 and S4 show that there is no clear best

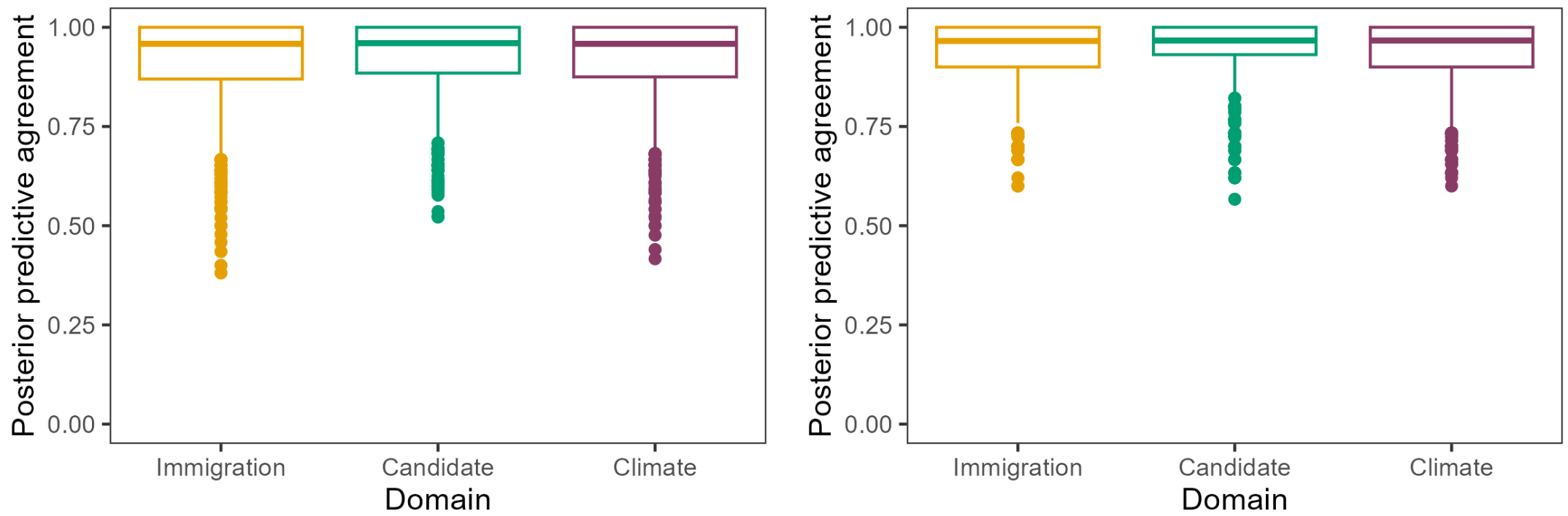

(a) No Ties **(b)** Ties

Fig. S4. High levels of descriptive adequacy for both conditions. Boxplots of posterior predictive accuracy (PPA): how well the model's predicted choices align with actual observed choices, where the edges of the box represent the interquartile range, the line in the box representing the median. The points represent individual subject outliers, the majority of which are subjects classified as Contaminant. The model can at best align perfectly with observed choices ($PPA = 1$). Recall that as the model likelihood is a Binomial and observed counts vary widely across subjects, it is difficult to determine an absolute lower threshold of the model performance.

model where one model "wins" across both metrics. In addition, all three substantive strategies (TTB, Tally, and WADD) are featured in models that exhibit the best average goodness of fit according to one of the metrics (TTB + Tally according to DIC and WADD according to WAIC). Further, Tables S3 and S5 show that there is substantial heterogeneity in the distribution of best fit models across individuals. Indeed more than 50% of subjects are *not* best described by the ostensibly best average fit models according to DIC (TTB + Tally) or WAIC (WADD) in Experiment 1 Condition 1. That such a high percentage of subjects' individual best fit was not captured by these two subsetted models, along with the fact that all three substantive strategies (TTB, Tally, and WADD) factor into different models with the best average fit, provides strong empirical justification that existing variation in individuals' behavior is best captured by a more expansive model. We therefore present the results of the 4 strategy model in the paper, as it is best equipped (and indeed the only one equipped) and best motivated from a theoretical and empirical perspective to fully capture the heterogeneity present among our subjects.

Model	DIC	WAIC
2: TTB	691930	692428
2: WADD	690032	682781
3: TTB + Tally	687445	688343
3: TTB + WADD	689864	683685
4: All	690499	684265

Table S2. Average Goodness of Fit for E1C1. Each cell under the DIC and WAIC columns represent the DIC and WAIC for all subjects.

Model	DIC	WAIC
2: TTB	228	166
2: WADD	140	468
3: TTB + Tally	390	141
3: TTB + WADD	124	117
4: All	78	68

Table S3. Distribution of best-fit model at the individual level for E1C1 ($N = 960$). Each cell represents the number of subjects for whom the corresponding model (row) was the best fit according to a given metric (column).

Model	DIC	WAIC
2: TTB	775150	767714
2: WADD	781019	759297
3: TTB + Tally	774894	767501
3: TTB + WADD	778888	760388
4: All	781898	761553

Table S4. Average Goodness of Fit for E1C2. Each cell under DIC and WAIC represents the DIC and WAIC for all subjects.

Model	DIC	WAIC
2: TTB	432	173
2: WADD	112	582
3: TTB + Tally	344	66
3: TTB + WADD	76	132
4: All	40	51

Table S5. Distribution of best-fit model at the individual level for E1C2 ($N = 1004$). Each cell represents the number of subjects for whom the corresponding model (row) was the best fit according to a given metric (column).

B.2. Model Certainty. We additionally consider model certainty: how confident is the model in its strategy classification for each subject? Is the model extremely confident (e.g. all the posterior weight is on 1 strategy) or is it extremely uncertain (e.g. the posterior weights are uniformly distributed across all possible strategies). To assess this, we calculate a Model Certainty Score:

$$\text{Model Certainty (MC)} = \frac{\text{Posterior frequency of modal strategy classification}}{\#\text{ posterior iterations}}$$

The MC thefore has a minimum of 0.25 (equal probability of any strategy) and a maximum of 1 (100% probability of one strategy). In Condition 1, the median model certainty across all domains is 0.83 (Immigration: 0.82, Candidate: 0.83, Climate:

0.83), Figure S5a. In Condition 2, with ties, the median model certainty is unsurprisingly lower across all domains at 0.66 (Immigration: 0.65, Candidate: 0.68, Climate: 0.66), Figure S5b. Taken together, these metrics demonstrate not only that our model describes the observed data well, but that it is also reasonably confident in its classification.

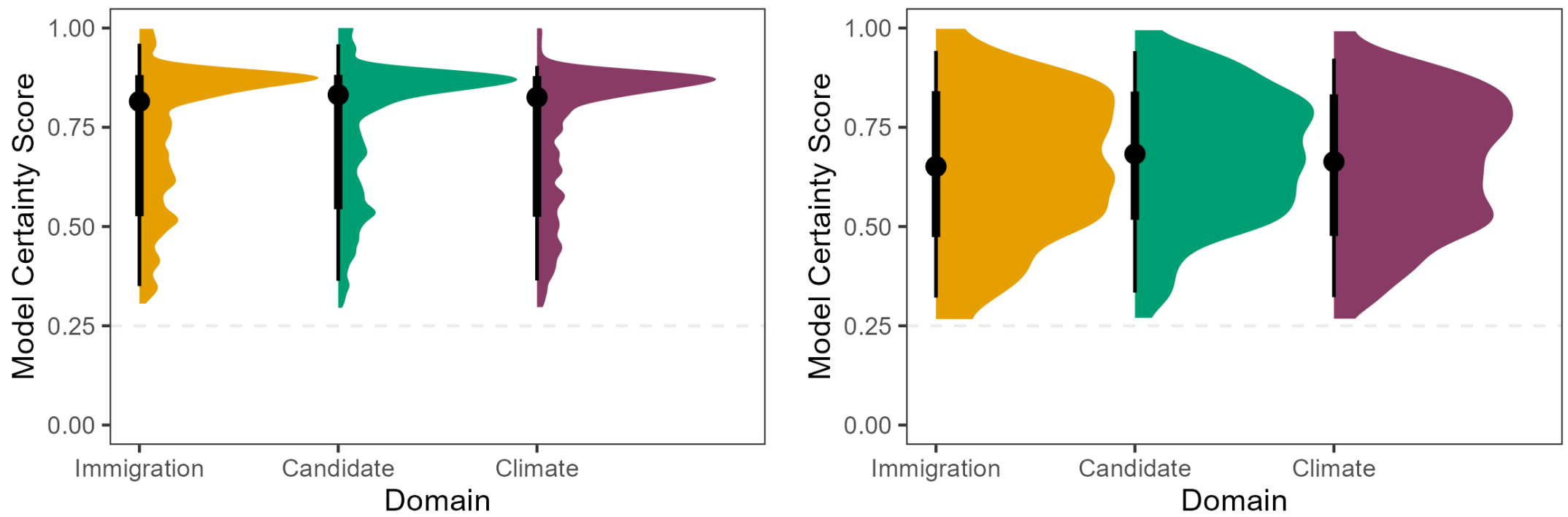

(a) No Ties

(b) Ties

Fig. S5. High levels of model certainty. The point estimate represents the median, with the thicker black line representing 66% of the density and the thinner black line representing 95%. The dashed grey line represents the lower limit of model certainty of 0.25 (chance).

B.3. Aggregate results on attribute weights. Here, we consider the attributes our subjects assign the most weight to on average in each domain. The attributes with the highest average posterior weights for the Candidate and Climate domain are Age (C1: $w = 0.81$, C2: $w = 0.83$) and Average Monthly Household cost respectively (C1: $w = 0.81$, C2: $w = 0.81$). Recall that attribute weights can take on any value between 0.5 and 1. For the Immigration domain, Immigrant Type has the highest average posterior weight (C1: $w = 0.78$, C2: $w = 0.79$), followed closely by Age (C1: $w = 0.77$, C2: $w = 0.78$). As shown in Table S6, these results qualitatively align with two other measures of attribute weight/importance that can be inferred outside of the model: (1) subject-provided ratings assigning each attribute a value between 0 and 10 indicating its importance in their decision process and (2) the model-free Average Marginal Component Effect (AMCE), widely used in political science and estimated at the domain – not individual – level. The one minor exception is an inconsistency between the Model Inferred/User Provided Ratings and the AMCE in the Immigration domain (C1), which could plausibly be explained by differences in how the various metrics are aggregated.[‖]

Measure	Immigration		Candidate		Climate	
	C1	C2	C1	C2	C1	C2
Model Inferred	Type	Type	Age	Age	Cost	Cost
User Rating	Type	Type	Age	Age	Cost	Cost
AMCE	Age	Type	Age	Age	Cost	Cost

Table S6. General convergence in highest weighted attribute as measured three different ways. Each cell provides the most important attribute for Condition 1 (No Ties) and Condition 2 (Ties), on average across all subjects. Row 1 contains the model-inferred most favored attribute. Row 2 contains the subjects' self reported most favored attribute. Row 3 contains the attribute with the highest AMCE.

In addition, we briefly note here that we also estimated and evaluated the AMCEs for all of the attributes in each domain and condition. While estimation of AMCEs is not a core focus or interest in this study, we perform this assessment as a preliminary validation of the quality of our data (in the aggregate): AMCEs are the dominant estimand of interest in political science conjoint research and hence we can compare our AMCE estimates to past research to ensure that the subjects in our sample are not engaging in aberrant choice behavior (in the aggregate) relative to the established literature. The results, shown in Figure S16, reveal AMCE estimates that are broadly consistent with past applied research in all three of our domains in terms of the directionality and relative magnitudes of the AMCEs (e.g. the strong influence of skills-based considerations and weak influence of geographic origin in the immigrant choice domain, and the strong influence of household cost in the climate policy domain) (19, 26, 37–40). This gives us confidence in our data and alleviates concerns about data quality degradation as a result of too many trials—consistent with past research showing no substantial evidence of respondent fatigue or inattention over comparable numbers of trials (41–43).

We next more generally evaluate to what extent the model-inferred individual attribute weights match the user-provided attribute ratings beyond the average highest weighted attribute. As the AMCE is also calculated from choice behavior just like the model-inferred weights, we do not include it in this comparison. We therefore first compare the degree of correspondence between the model-inferred most important attribute and the user provided. We find 78.3%, 80.6%, and 81.3% agreement in Condition 1 and 77.2%, 79.1%, and 78.9% agreement in Condition 2 for the Immigration, Candidate, and Climate domains respectively. The more attributes we consider, the greater discrepancy between which the model inferred ranking of attributes and the user provided – C1: (53.1%,54.3%,46.1%) agreement between subject provided rating and model inferred rating for the top 2 attributes, and (22.9%, 22.8%, 20.5%) agreement for the top 3. In C2, we find (59.8%,60.9%,53%) agreement between subject provided rating and model inferred rating for top 2 attributes, and (32.5%, 32%, 25.4%) agreement for top 3. For additional results on the average ordering of attributes and the most favored attribute levels on average, see Table S9.

Given the high descriptive adequacy of the model with respect to choice behavior, presented earlier, the discrepancy between these rankings should not be viewed as a sign of model insufficiency—the model is, after all, a model dealing with choice behavior. Instead, we view this as evidence that while participants may be good at articulating what is most important to them, they may be limited in expressing how those factors play into their decision process. Interestingly, and perhaps relatedly, there is better agreement in the Ties condition compared to the No Ties condition once we look beyond the single most important attribute. This could be because individuals are forced to evaluate more attributes independently of any strategy they use to make their decision: this additional engagement might result in subjects having increased access to their internal ordering of attributes.

B.4. Individual-level model classification results. To validate our model's strategy classification, we consider our model-based results' correspondence with non-parametric measures.

To do so, we first estimate individual-level versions of the AMCEs – that is, Individual Marginal Component Effects (IMCEs) (44) – which can be considered intuitive approximations of individuals' attribute weights and have the benefit for validation purposes of being model-free.[**] We then compute the Herfindahl–Hirschman Index (HHI) of the IMCEs for each subject in

[‖] As discussed in (36), one cannot expect exact correspondence at all times between different estimands. In the present case, we can consider how the weights are distributed across attributes. In the Candidate and Climate domains, there appears to be consensus across subjects that a single attribute (Candidate Age and Average Monthly Household Cost, respectively) matter the most, and by far. This is not the case for the Immigration domain. Although Immigrant Type is most often the highest weighted attribute, Age, Highest Education, and to a slightly lesser degree, English Proficiency all have inferred weights that are non-zero on average. *This* result is indeed consistent across all three measures. As such, it makes sense that the correspondence between the three measures would be more brittle in the Immigration domain.

[**] As shown elsewhere, the non-parametric estimator of AMCEs can be implemented conveniently using linear regression (45), which also extends to the individual-level IMCEs (44), and hence we employ individual-level linear regressions for our estimation.

each domain. The HHI is a commonly used measure of concentration/diversification (and has the same formula as the Simpson index). To compute the HHI of the IMCEs (while maintaining the HHI's interpretability), we take the absolute value and then normalize the IMCEs to sum to 1 for each subject in each domain. As such, the lower the HHI, the more diffusely weights are spread across attributes (i.e. they are less concentrated on one or few attributes). In addition, as a complementary heuristic, we also count the number of IMCE estimates for each subject in each domain that are statistically significant (at the conventional level $\alpha = 0.05$).[††] Finally, we break these results down by strategy classification.

The results, shown in Table S7, reveal close correspondence in both conditions between these non-parametric heuristic measures and the model strategy classifications, based on the individual-level behavior we should theoretically expect for subjects being classified as using different strategies. First, subjects classified as TTB have the highest HHI across the board, a value of 1 for the median HHI given no ties, and the lowest median number of significant coefficients among the non-contaminant strategies (with only 1 significant coefficient given no ties). As expected, the HHI decreases and significant coefficients increase in the presence of ties. Second, not only do the subjects classified as using the "full information" strategies have lower HHI, but the HHI for the Tally subjects is indeed lower than that of the WADD subjects across the board; this should be expected, since WADD allows for differential weights (and hence more concentration) whereas Tally assumes equal weights across attributes. Finally, for Contaminant subjects, the median HHI is comparably low like the full-information strategies but the median number of significant coefficients is 0, which makes sense as the weights should be distributed relatively evenly for Contaminants due to randomness but the IMCEs should tend to all be small in magnitude and statistically insignificant.

Strategy	Immigration				Candidate				Climate			
	C1		C2		C1		C2		C1		C2	
	HHI	β	HHI	β	HHI	β	HHI	β	HHI	β	HHI	β
TTB	1.00	1	0.36	2	1.00	1	0.36	2	1.00	1	0.36	2
WADD	0.27	3	0.26	2	0.27	3	0.27	2.5	0.29	3	0.28	2
Tally	0.23	3	0.24	2	0.23	3	0.23	2	0.24	3	0.22	2
Contaminant	0.24	0	0.23	0	0.24	0	0.24	0	0.24	0	0.24	0

Table S7. Strategy classifications correspond with non-parametric measures (I): IMCE. For each condition and domain, we provide the between-subject median HHI – calculated from individual level IMCEs for each subject – and the corresponding median number of significant IMCE coefficients, shown in the columns labeled β. The lower the HHI, the more distributed the weights are across attributes.

We further find that, for all three domains, the median HHI for C2 is significantly lower than the HHI for C1 (Immigration: $HHI_{C1} = 0.46, HHI_{C2} = 0.30$, Candidate: $HHI_{C1} = 0.50, HHI_{C2} = 0.30$, Climate: $HHI_{C1} = 0.54, HHI_{C2} = 0.31$, Bayesian t-tests of difference in means, all Bayes Factors $> 9 \times 10^{101}$). That is, the IMCEs are distributed across more attributes in C2 than they are in C1. This is consistent with the more diffuse spread of attribute weights observed in the Ties condition (Figure 4b, Main Text) relative to the No Ties condition (Figure 4a, Main Text). Further, recall that subjects do not know which attribute levels will be tied on each trial, necessitating the more diffuse distribution of weights across attributes in both our model-free and model-based measures.

Finally, we also consider response times (RT) as another non-parametric corroboration of strategy classification. The Materials and Methods section in the main text presents the analyses. In Figure S6 below, we provide additional results broken down by domain and including subjects regardless of their average RT.

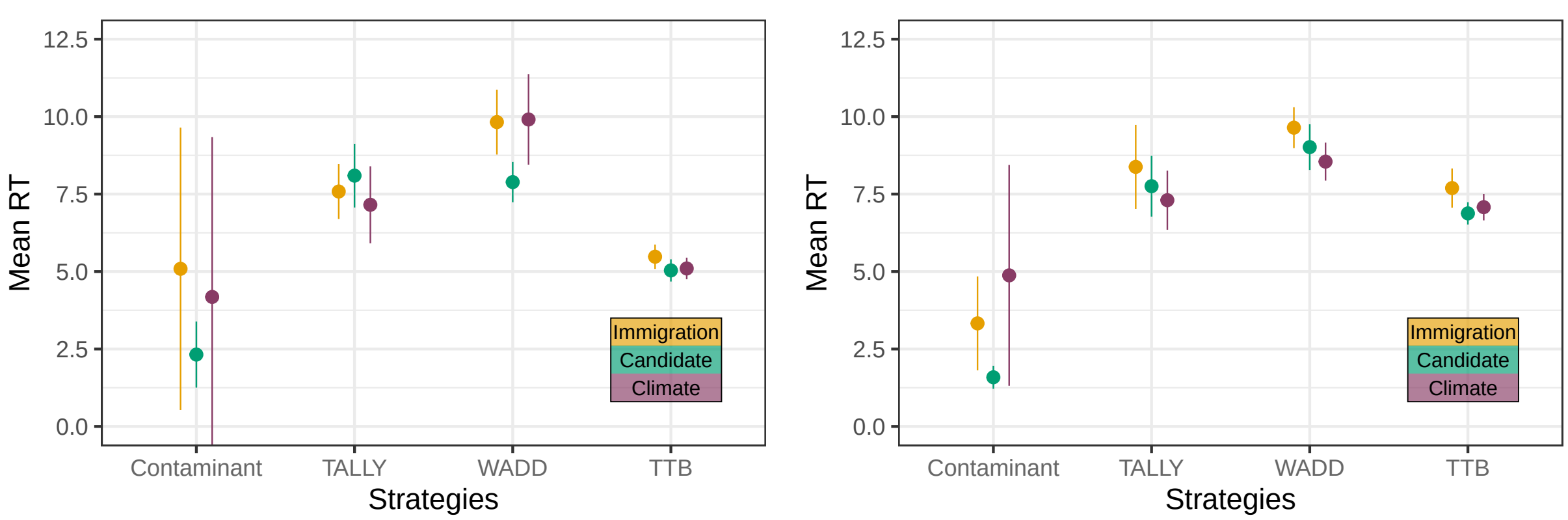

(a) No Ties

(b) Ties

Fig. S6. Strategy classifications correspond with non-parametric measures (II): Response Time.

C. Parameter Recovery. Our parameter recovery analyses involve generating data in which simulated subjects take our conjoint experiments and make their choices according to known strategies. We subsequently apply our model to the resulting simulated

[††] Note, however, that this metric is quite limited by the fact that each individual-level IMCE is estimated with a relatively small amount of data.

data and assess its classification performance (i.e. the model's ability to infer simulated subjects' strategies) under a variety of different conditions, which we describe in detail below. In the subsections that follow, we consider the model's classification performance when applied to simulated subjects executing each of our strategies, starting with subjects using WADD.

WADD recovery. Strictly speaking, TTB and Tally are mathematical subsets of WADD. The Tally algorithm can be implementationally equivalent to WADD with uniform weights, and TTB can be implementationally equivalent to WADD with non-compensatory weights or WADD with compensatory weights but implemented in a non-compensatory manner[‡‡]. To be sure, we cannot make process-level distinctions given choice data alone. However, we are able to identify when behavior is most consistent with what a TTB or Tally individual would do, insofar as it makes sense for an individual to be any one strategy. We can therefore expect that behavior generated from the WADD algorithm in conjunction with uniform attribute weights will be classified as Tally. Similarly, we can expect that behavior generated from WADD but using non-compensatory weights will be classified as TTB. This is because both Tally and TTB have rigid definitions in contrast to the more flexible WADD. Consequently, in our model, WADD classifications capture non-contaminant behavior that is consistent with compensatory weights being applied in a compensatory way. The philosophical implications of this, including what it means to "accurately" classify strategy, are discussed further in the Discussion.

There are three parameters we can manipulate in our recovery analysis that have core implications for our latent-mixture classifications:

- **Attribute weights**: How do different sets of attribute weights shape latent-mixture classifications?
- **Conjoint experiment structure**: As conjoint designs involve randomization, how do different experimental structures (i.e. pairs of conjoint profiles in a block) shape latent-mixture classifications?
- **Strategy fidelity**: How does the accuracy with which someone executes the strategy they are following shape latent-mixture classifications?

Attribute Weights. For our simulated subjects executing WADD, we choose 6 qualitatively different categories of attribute weights to test recovery (see Table S8, and recall that attribute weights are implemented using log odds in the WADD strategy). We choose two categories of non-compensatory weights, three categories of compensatory weights, and one set of uniform weights. The non-compensatory weights should render WADD and TTB identical in choice and the uniform weights should render the WADD and Tally identical in choice. Therefore, holding all other variables constant, we would expect "misclassification" of WADD as TTB and Tally (strategies explicitly defined to be non-compensatory and following uniform weights) in these circumstances. However, we would expect WADD to be correctly classified for the three compensatory weight categories.

Weight Category	Description	Values
Non-Compensatory High	Weights are non-compensatory and there is a large difference between the favorite weight and the rest	a. 0.65, 0.995, 0.510, 0.520, 0.550, 0.6<br>b. 0.55, 0.62, 0.51, 0.99, 0.51, 0.58<br>c. 0.65, 0.52, 0.81, 0.55, 0.51, 0.993
Non-Compensatory Med	Weights are non-compensatory and there is a medium difference between the favorite weight and the rest	a. 0.62, 0.90, 0.51, 0.60, 0.55, 0.53<br>b. 0.71, 0.51, 0.90, 0.61, 0.60, 0.54<br>c. 0.53, 0.72, 0.90, 0.60, 0.58, 0.51
Compensatory 2-High	Weights are compensatory and there are two dominant or highly valued attributes	a. 0.62, 0.95, 0.51, 0.85, 0.55, 0.70<br>b. 0.71, 0.51, 0.83, 0.61, 0.93, 0.54<br>c. 0.53, 0.72, 0.95, 0.60, 0.88, 0.51
Compensatory 3-High	Weights are compensatory and there are three dominant or highly valued attributes	a. 0.62, 0.90, 0.51, 0.85, 0.55, 0.80<br>b. 0.88, 0.51, 0.85, 0.61, 0.90, 0.54<br>c. 0.53, 0.88, 0.90, 0.60, 0.85, 0.51
Compensatory Cluster	Weights are compensatory and have relatively low spread	a. 0.76, 0.72, 0.68, 0.60, 0.56, 0.52<br>b. 0.78, 0.74, 0.70, 0.64, 0.58, 0.53<br>c. 0.70, 0.67, 0.64, 0.61, 0.58, 0.55
Uniform	Weights are equal	a. 0.75, 0.75, 0.75, 0.75, 0.75, 0.75<br>b. NA<br>c. NA

Table S8. Attribute weight distributions for WADD recovery. Each row represents a set of weights given to the six attributes for a particular simulation run. There are 6 different scenarios mapping onto qualitatively different weight allocations (i.e. non-compensatory high, non-compensatory med, etc.), noted by the Weight Category column and described in the Description column. For each category, there are 3 different possible weight sets, shown in the Values column. Recall that in the WADD strategy, all attribute weights are implemented using log odds.

To ensure that the values of the synthetic weights were realistic, we consulted the weights inferred by the model for our real subjects who were classified as different strategies. Then, for each weight category, we chose spreads of the weights to ensure

[‡‡] Recall that a compensatory set of weights means that the most highly weighted attribute does not dominate over the others in the set such that if the respondent weighted and summed together the remaining attributes, the sum total could be greater than the weight of the highest ranked attribute. That is, the other attributes can *compensate* for the highest ranked attribute. If a set of weights is non-compensatory, then the opposite is true: the highest ranked attribute cannot be compensated for by the remaining attributes. That is, the highest ranked attribute is the only one that matters.

we achieved the appropriate compensatory vs. non-compensatory vs. uniform dynamics described for each category. Recall that the values shown in the table were mapped through the log-odds transformation – which is why, for example, the vector [0.65, 0.995, 0.510, 0.520, 0.550, 0.6] is highly non-compensatory, with the log-odds of 0.995 far outweighing the sum of the log-odds of all the other values.

Conjoint Experiment Structure. As conjoint experiments must be randomized for each trial, respondents experience different choice pairs over the course of each block. While the randomization protects against bias and allows for causal inference, it also means that some individuals are presented more informative sets of conjoint profiles for strategy inference than others. Specifically, some individuals may by chance have several trials where choice predictions from strategies are distinct (e.g., WADD says choose Profile 1 and TTB says choose Profile 2). This naturally provides the model greater signal for strategy inference compared to choice sets where the choice predictions largely converge. To address this, we do not specifically manipulate the informativeness of the experiment structure. Instead, we use the real experiment structure experienced by 500 randomly sampled respondents for Experiment 1 Condition 1 (no ties) and Experiment 1 Condition 2 (ties). Those randomly sampled real experiment structures are then used and the simulated choices from them are generated according to our simulated respondents' execution of their strategies in conjunction with their synthetic attribute weight schemes.

Strategy Fidelity and Noisy Choice. We do not expect individuals to perfectly execute their strategy for every single trial – indeed such an expectation is highly ecologically invalid. Indeed, we explicitly infer strategy fidelity in the model (γ_{id}) and find that our respondents adhered to their classified strategy with a high degree of accuracy for both conditions: E1C1 mean fidelity 0.90, median 0.94, E1C2 mean fidelity 0.89, median 0.92. In our simulations, we allow for perfect execution of strategy as a baseline and also include two additional scenarios in which we add noise to the strategy execution. In the first noisy scenario, our simulated respondents execute their strategy perfectly in 90% of their choices, with the remaining 10% being noisy choice determined by chance (coin flip). In the second noisy scenario, our simulated respondents execute their strategy perfectly in 80% of their choices, with the remaining 20% being noisy choice determined by chance (coin flip).

We take all these together and run 500 simulated subjects 6 (different weight categories) x 3 (different levels of noise) = 18 times, and we apply them to the sampled conjoint experiment structures for each of the three domains. Specifically, for each randomly sampled conjoint experiment structure, we generate a simulated subject whose attribute weights following each of the weight schemes presented in Table S8. We also randomly set the favored levels for each subject. Then, for each trial in the randomly sampled conjoint experiment structure, we take the given pair of conjoint profiles, attribute weights, and favored levels, and make the choice whatever WADD predicts, adding (or not) the appropriate level of noise to choices depending upon the scenario. We begin by reporting results from the noiseless recovery analysis. Note that for the simulated subjects there is no difference in behavior across domains (as the simulated subjects simply follow their programmed mathematical rules), and we therefore report average results across domains.

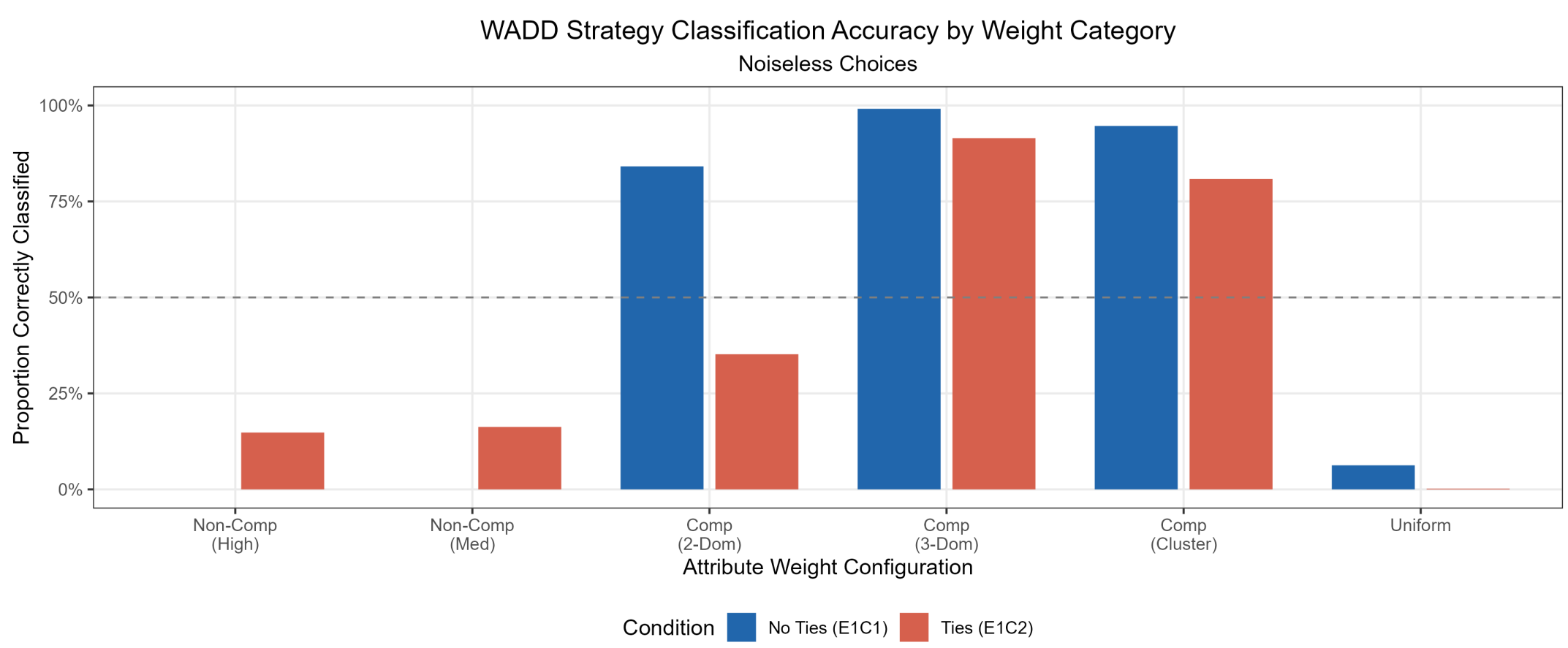

Fig. S7. Accuracy for WADD classifications across different weights. Modal classification per simulated subject. 500 simulated subjects for each of the 3 domains = 1500 data points per bar. With noiseless data, WADD is most often correctly classified when attribute weights are compensatory.

Results: WADD classification accuracy given noiseless choices. Our recovery analysis shows exactly what we expect: WADD classification accuracy is highly dependent on the attribute weight structure, as shown in Figure S7. When weights are compensatory, WADD is correctly classified 93% in the no ties condition, and 69% in the ties condition. However, these numbers drop drastically when considering the non-compensatory and uniform weights. For non-compensatory weights, subjects are *never* classified as WADD in the no ties condition, and approximately 15% of the time in the ties condition (across both non-compensatory high and medium). We attribute this difference to the structural distinctions between ties and no-ties – in the ties condition, there is more overlap in the choice predictions generated by WADD and the other strategies. We see a similar pattern for the uniform weights, in that subjects are *almost never* classified as WADD in both the ties and no ties condition. It is worth noting that adding noise to choices softens this distinction somewhat, as described later.

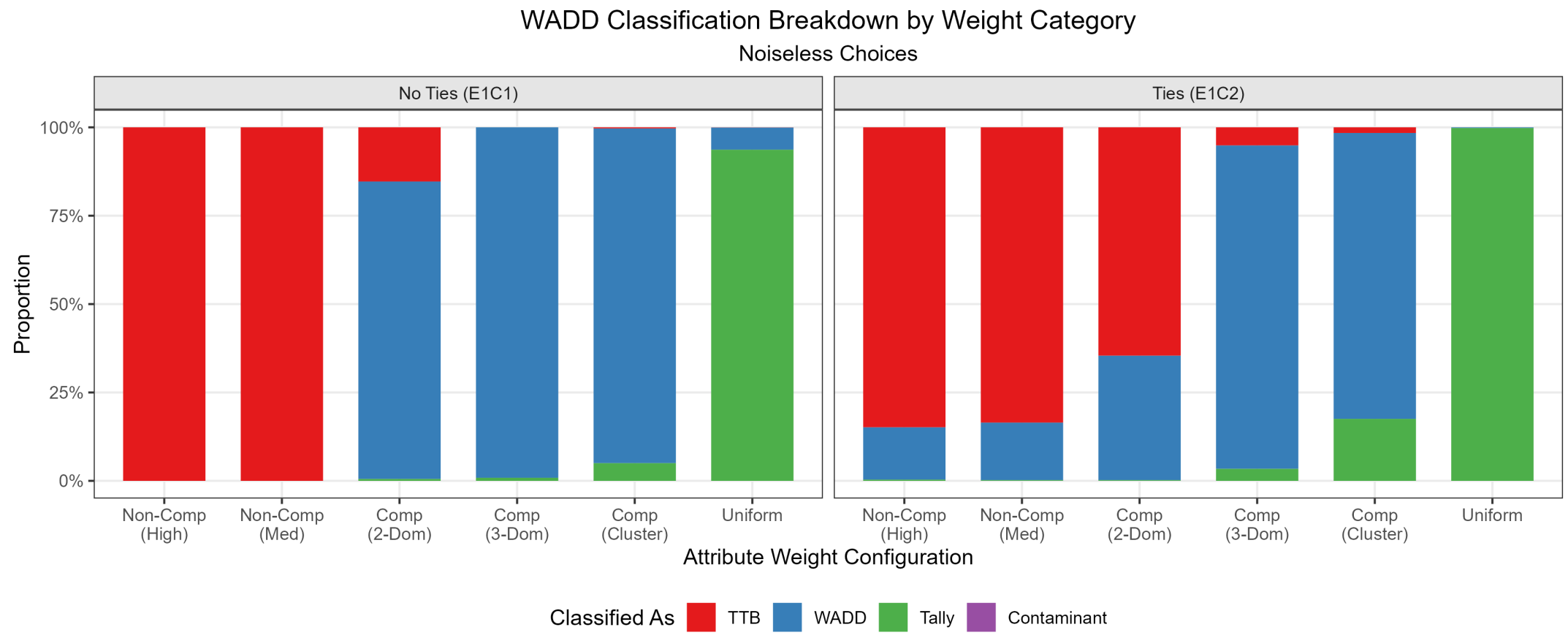

Fig. S8. Actual classifications for data simulated as WADD across different weights. Modal classification per simulated subject. 500 simulated subjects for each of the 3 domains = 1500 data points per bar. With noiseless choices, WADD generated behavior is overwhelmingly classified as TTB when attribute weights are non-compensatory and Tally when attribute weights are uniform. This holds across both main experimental conditions. Compensatory weights where only two attributes are dominant also lead to majority TTB classification when ties are present.

We show the actual classifications of the simulated WADD data in Figure S8. We see that WADD is *always* classified as TTB when weights are non-compensatory and there are no attribute ties. This is consistent with our prediction outlined earlier. The inclusion of ties, as expected, reduces the dominance of TTB classification, with WADD being classified 15% of the time, on average, with ties and noiseless choices. We similarly see that Tally is almost always classified when weights are uniform. We also see that the inclusion of ties reduces the accuracy with which WADD is classified when weights are compensatory but have two dominant attributes. This makes sense because ties force the evaluation of multiple attributes even with the TTB algorithm, leading to a greater overlap in choice predictions from both WADD and TTB. Overall, we take these results as evidence that the model is working correctly because we expect these "misclassifications." Indeed, if a person was truly WADD but was behaving in a way that was most consistent with TTB or Tally, what *should* we classify them? What does it mean to have behavior generated from one (flexible) strategy algorithm but have it align closely or even perfectly with another, more precisely defined strategy? We explore these questions in more detail in the Discussion.

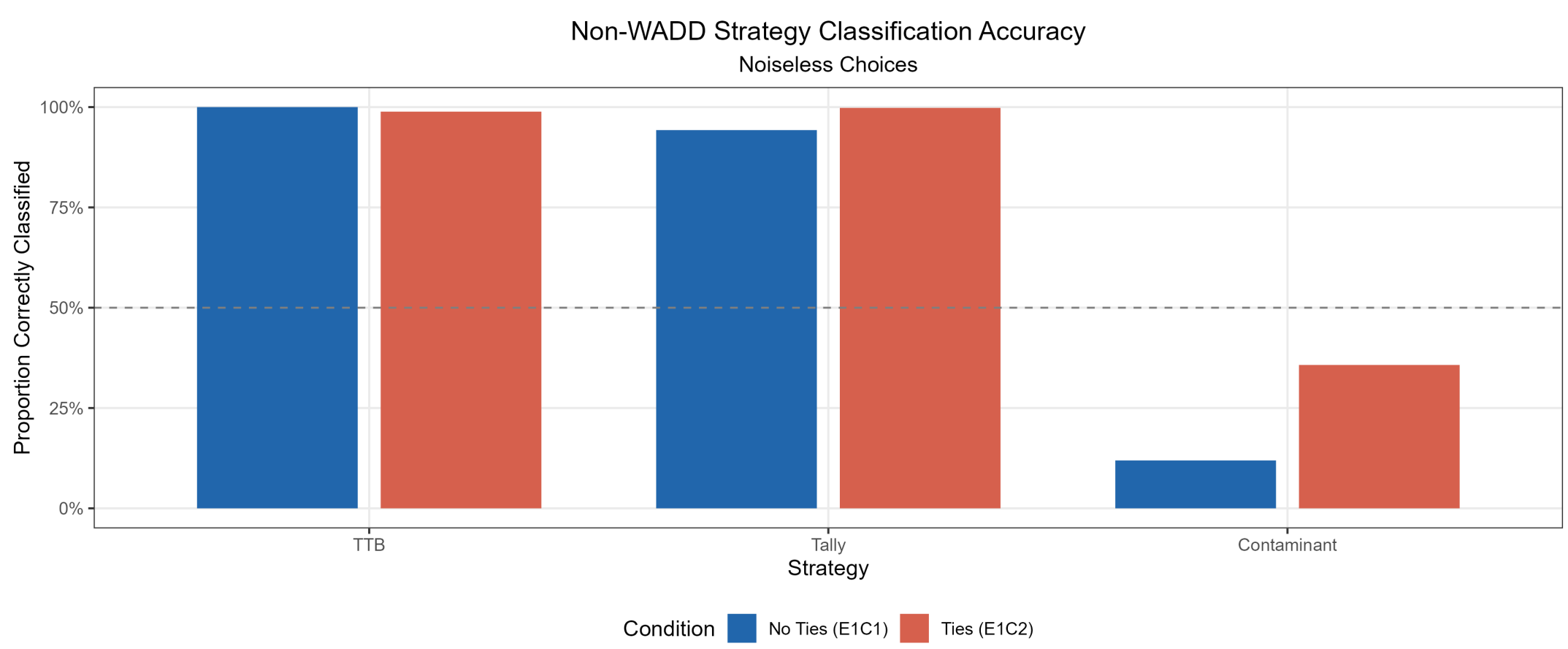

Fig. S9. Accuracy for TTB, Tally, and Contaminant classifications. Modal classification per simulated subject. 500 simulated subjects for each of the 3 domains = 1500 data points per bar. Classification accuracy is high for TTB and Tally for noiseless choices.

TTB, Tally, Contaminant recovery. We follow a similar process in performing these recovery analyses for the three remaining strategies. While we do not manipulate attribute weights, as none of these strategies use attribute weights, we use the same 500 randomly sampled real experiment structures and simulate the choices for those experiment structures according to each strategy under all three levels of noise.

Results: TTB, Tally, Contaminant classification accuracy given noiseless choices. TTB and Tally generated data recover with almost 100% accuracy given noiseless choices, Figure S9. Interestingly, this is not the case for contaminant data, which is accurately classified approximately 25% of the time on average: 12% of the time in No Ties and 36% of the time with Ties. Recall that the contaminant data is generated from a coin flip, providing a ceiling of 50% for optimal classification. This suggests that our model underestimates contaminant data.

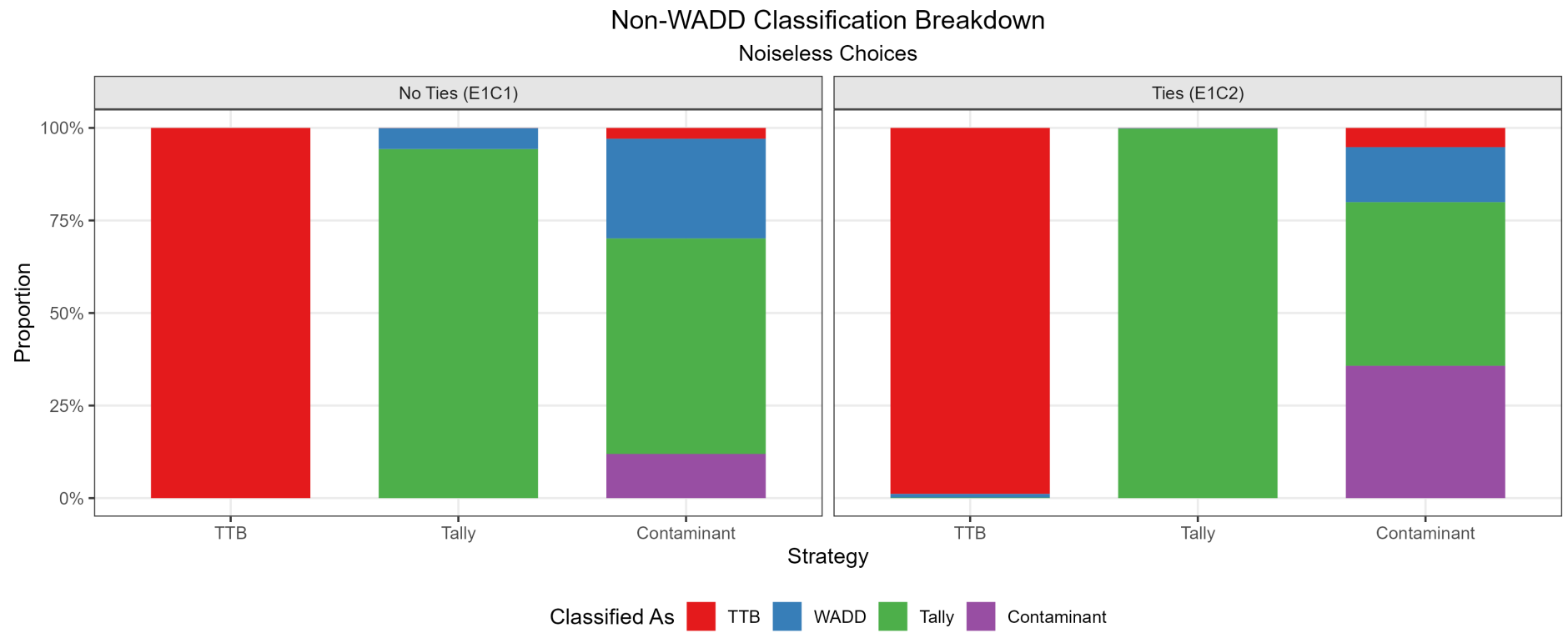

Fig. S10. Actual classifications for data simulated from TTB, Tally, Contaminant strategies given noiseless choices. Modal classification per simulated subject. 500 simulated subjects for each of the 3 domains = 1500 data points per bar. Contaminant-generated behavior (produced by coin flip) shows poor recovery, with the majority of simulated subjects being classified as Tally. Note, however, that a coin flip can at best be accurately classified 50% of the time so the threshold for accurate classification is very different compared to TTB, Tally, and WADD.

When we break down the classifications themselves in Figure S10, we find that TTB and Tally are almost always classified correctly. Importantly, we see that the majority of simulated Contaminant subjects are actually classified as Tally. This suggests that our results overreport the frequency of Tally subjects, as they may include true contaminants.

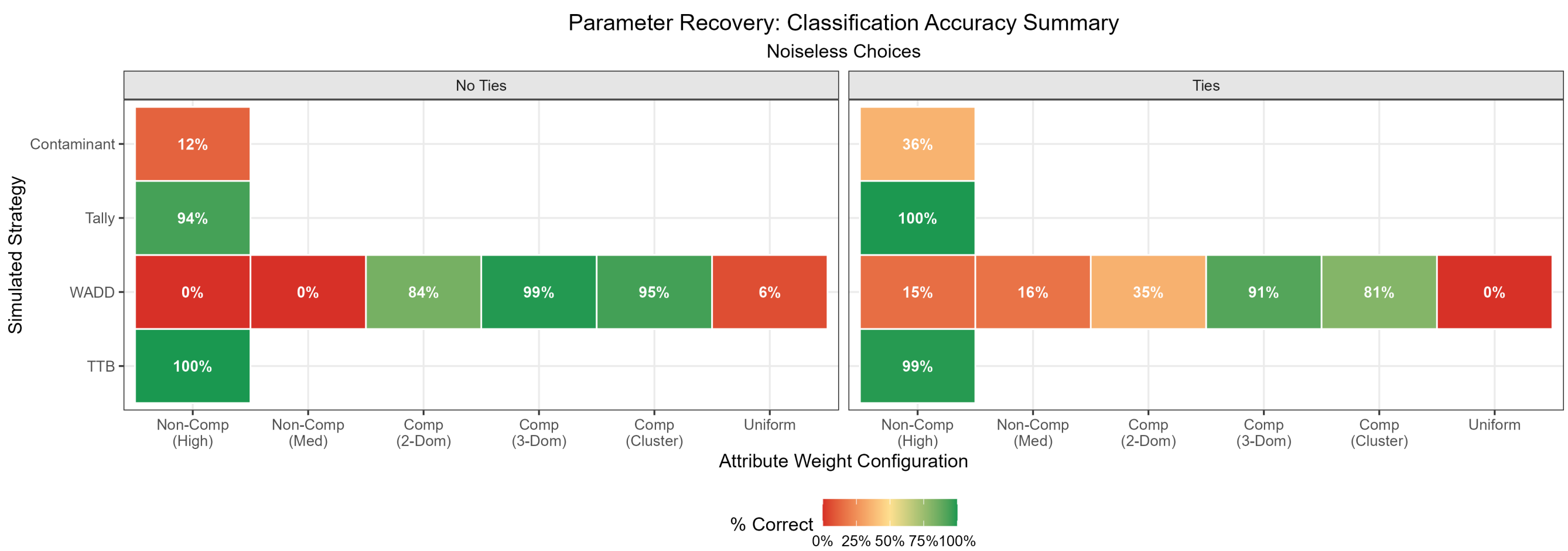

Fig. S11. Heatmap of classification accuracies. We place TTB, Tally, and Contaminant recovery in the first column purely for visual convenience as, recall, no attribute weights are used for these strategies.

Results: Adding Noise to Choices As described above, we also ran our simulations with 10% and 20%“noisy” choices coming from a coin flip . The results are shown in Figure S12, which highlights that classification accuracy degrades but remains good. The same broader pattern of classification accuracy holds as with noiseless data, where WADD recovery for non-compensatory and uniform weights is particularly low (and indeed is usually TTB and Tally, Figure S13). Similarly, TTB and Tally continue to have good recovery for both noisy simulations, with slightly worse performance for TTB in the Ties condition, Figure S14.

Taken together, our analysis shows three things clearly. The first is that our model is sensitive to the features it should be when recovering strategy classifications (attribute weights and noise). The second is that substantive strategies are reasonably well recovered when they are expected to be. Third, we find that contaminant behavior is not well recovered. As mentioned earlier, this tells us that we are underestimating the degree of contaminant behavior present in our data and most likely overestimating the prevalence of Tally-like behavior. This also tells us that our primary substantive result most likely to be impacted by this, that strategy classification distributions shift as a function of experiment design, is still valid as it is focused on the reduction of TTB classifications in more complex environments.

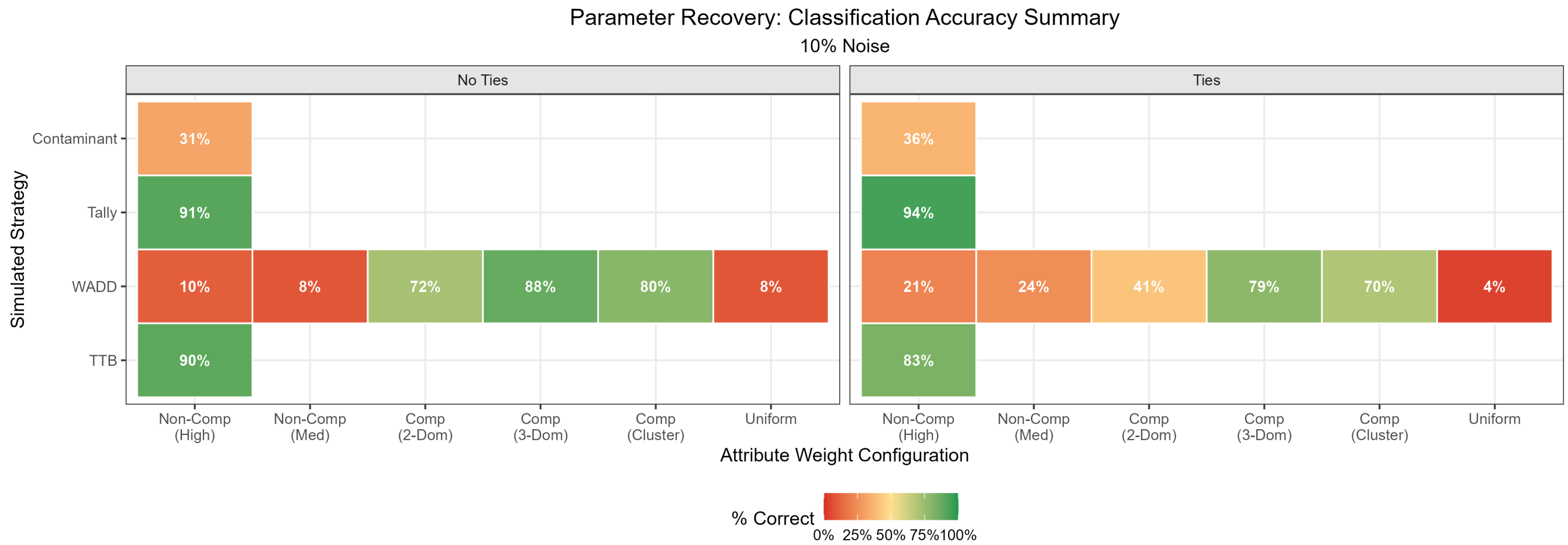

(a) 10% Noise

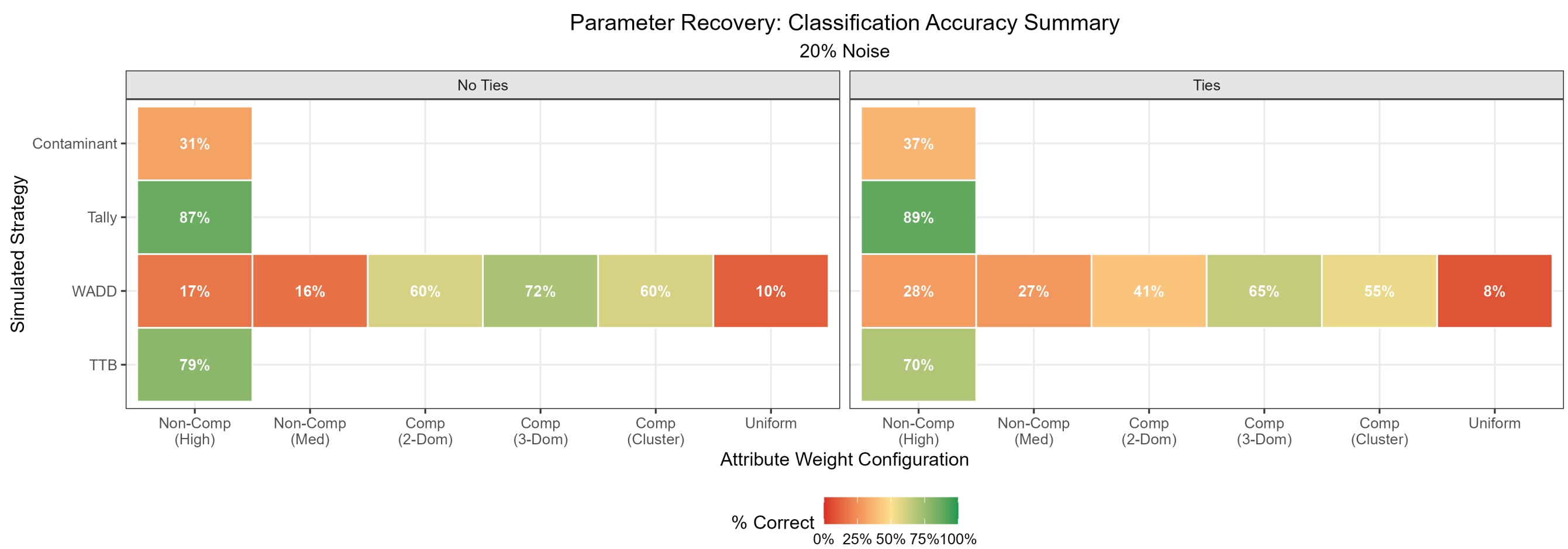

(b) 20% Noise

Fig. S12. Classification accuracy heatmap with noisy data. Plotted as described in Figure S11.

D. Additional Results: Experiment 1.

D.1. Favored attribute levels. We present the modal favored attribute level for each condition in addition to the percentage of subjects who preferred this level in Table S9.

D.2. Subject provided attribute ratings. At the end of each decision block, we asked subjects to assign every attribute a numerical value between 0 and 10, representing how much weight they assigned to that attribute during their decision making process. Importantly, we did not ask them to specify which attribute levels they cared about, just how much they care about any given attribute. Subjects, on average, distributed weights similarly for both conditions, Figure S15, and as reported in the main text, the highest rated attribute was consistent with both model-inferred and model-free (AMCE) measures. Despite this, we notably do not see the bimodality so evident in Figure 4, Main Text.

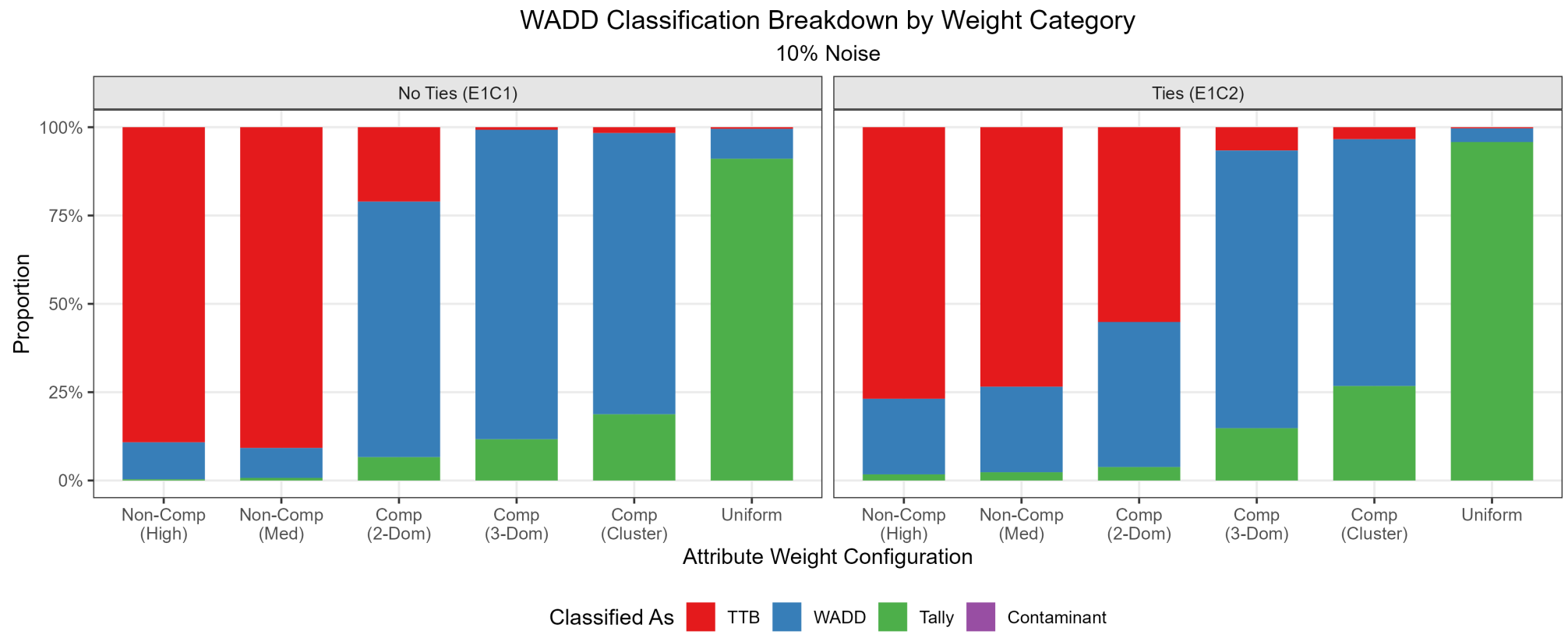

(a) 10% Noise

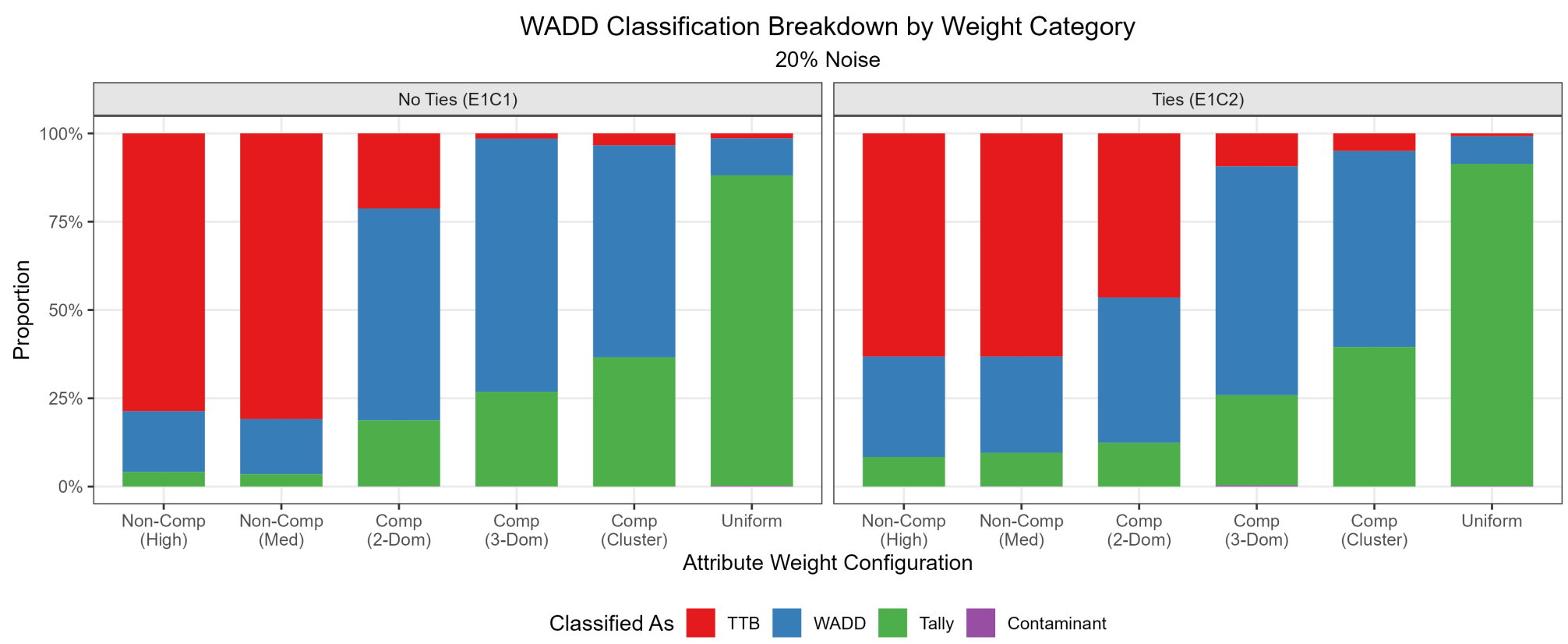

(b) 20% Noise

Fig. S13. Actual WADD classifications with noisy data. Modal classification per simulated subject. 500 simulated subjects for each of the 3 domains = 1500 data points per bar.

D.3. Model free attribute weight inference: AMCE. We estimate the gold-standard measure of data conjoint experiments in political science, the model-agnostic AMCE. The AMCE represents the relative causal importance of one attribute level over another on choosing a given profile, at the aggregate level. We find strong correspondence across the two conditions, Figure S16.

In the Immigration domain, subjects most prefer people who are younger, or are asylum seekers compared to people who are older or are economic migrants. They also, somewhat conversely, prefer potential immigrants who are more educated or fluent in English relative to the complementay attribute levels, two factors that allow potential immigrants to better contribute to the economy. Finally subjects prefer female relative to male immigrants, or, in C2 only, prefer potential immigrants from Central America compared to Sub-Saharan Africa. In the Candidate domain, subjects overwhelmingly prefer younger candidates relative to older. They also prefer candidates who have prior political experience, prior military experience, and are more educated relative to the complementary baseline. Subjects also prefer women or, in C2 only, Black candidates relative to male or White candidates. In the Climate domain, subjects strongly prefer policy packages with a lower monthly household cost relative to a higher. We also find that subjects prefer a higher percentage of emissions represented in the package, that any monitoring should be done by an independent commission, that countries should pay proportional to their own emissions, that there should be no householder-level sanctions, or that more countries should be involved in the policy relative to the complementary baselines. While qualitative patterns in the AMCE are in correspondence across both conditions, we find, for the attributes levels with the largest AMCEs, that these AMCEs are larger in magnitude in Condition 1 vs. Condition 2. For example, in the Candidate domain, respondents in both conditions most strongly disapproved of older relative to younger political candidates, but more so in C1, $AMCE(SE) = -0.438(0.01)$, compared to C2, $AMCE(SE) = -0.36(0.01)$. Likewise, in the Climate domain, respondents in both conditions most strongly approved of lower average monthly household costs relative to higher, but more so in C1, $AMCE(SE) = 0.44(0.01)$, compared to C2, $AMCE(SE) = 0.30(0.01)$. For the Immigration domain, the largest AMCEs are not equivalent across conditions, where subjects in C1 most prefer people below retirement (C1 $AMCE(SE) = 0.22(0.01)$, C2 $AMCE(SE) = 0.18(0.01)$) while subjects in C2 most prefer people who are not economic migrants (C1 $AMCE(SE) = -0.19(0.01)$, C2 $AMCE(SE) = -0.20(0.01)$). These results hint at the possibility that subjects may distribute weight more broadly over attributes when presented profiles that have ties, compared to without ties.

The most important attributes as identified by the mean AMCE and the average subject-provided rating for the Candidate and Climate domains are Age and Average Monthly Household cost respectively. For the Immigration domain, the highest

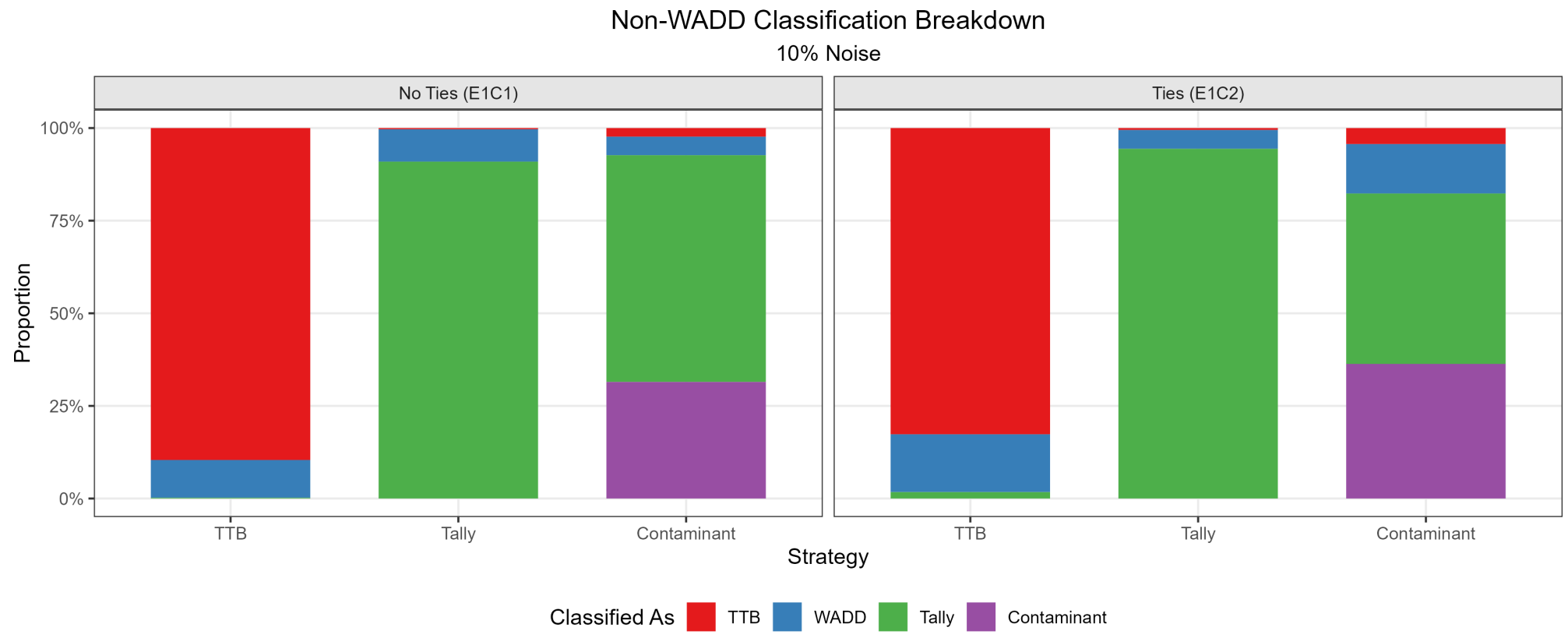

(a) 10% Noise

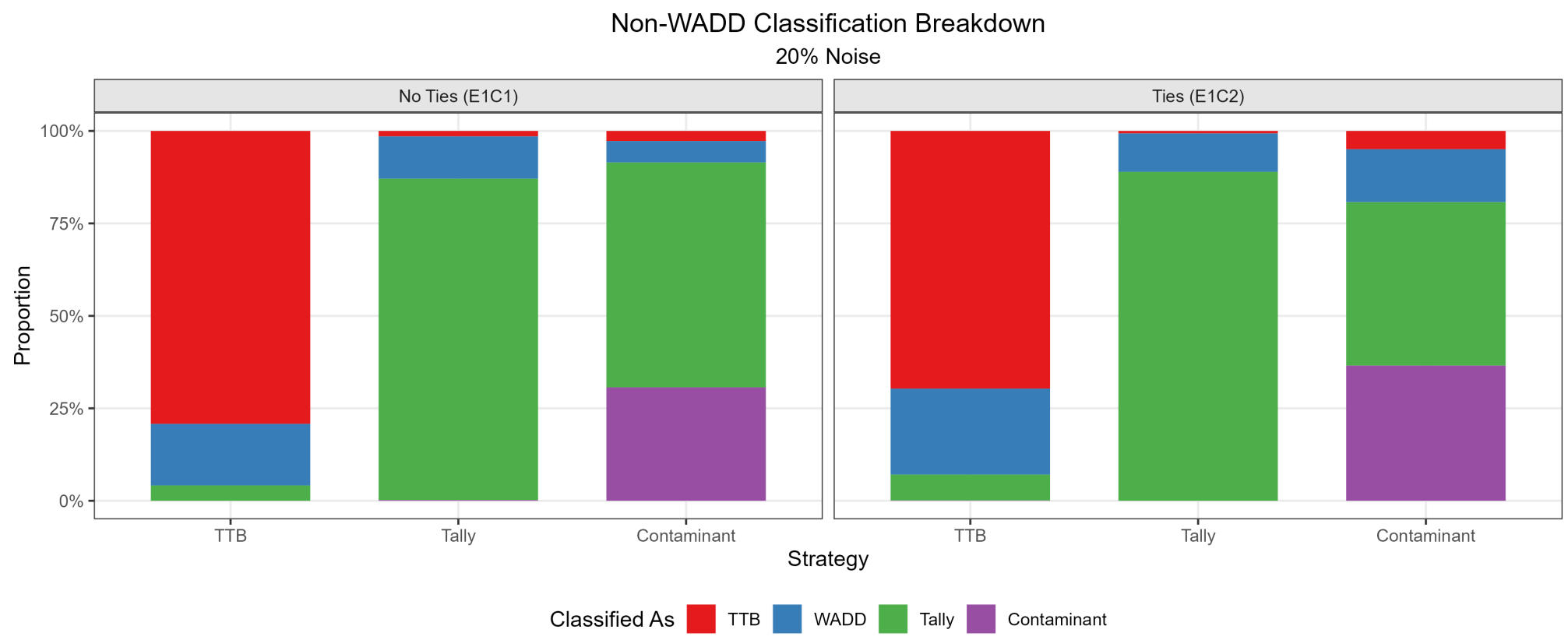

(b) 20% Noise

Fig. S14. Actual non-WADD classifications with noisy data. Modal classification per simulated subject. 500 simulated subjects for each of the 3 domains = 1500 data points per bar.

**Immigration Attributes**	**C1: Favored Level**	**C2: Favored Level**
Age	Below retirement (68%)	Below retirement (74%)
English Proficiency Level	Intermediate (69%)	Intermediate (79%)
Gender	Female (57%)	Female (63%)
Highest Education	College or above (69%)	College or above (80%)
Type	Asylum seeker (63%)	Asylum seeker (69%)
Region of Origin	Central America (51%)	Central America (51%)
**Candidate Attributes**	**C1: Favored Level**	**C2: Favored Level**
Age	50s (82%)	50s (90%)
Gender	Female (54%)	Female (58%)
Highest Education	Graduate degree (62%)	Graduate degree (70%)
Prior Military Service	Has served (66%)	Yes (73%)
Prior Political Office	Yes (68%)	Yes (71%)
Race	Black (52%)	Black (56%)
**Climate Attributes**	**C1: Favored Level**	**C2: Favored Level**
Avg Monthly Household Cost	$53 (79%)	$53 (83%)
Emissions Represented	80% (61%)	80% (71%)
Intl. Cost Distribution	Prop. emissions (54%)	Prop. emissions (55%)
Monitoring by	Indep. commission (54%)	Indep. commission (58%)
Monthly household sanction	None (58%)	None (67%)
Participating countries	160 (57%)	160 (69%)

Table S9. Model-inferred aggregate favored attribute levels. Each cell contains the model-inferred aggregate favored attribute level with the percentage of subjects for whom this was the modal posterior favored attribute level in parentheses. We abbreviate the attribute level "Proportion to current emissions" to "Prop. emissions" for brevity.

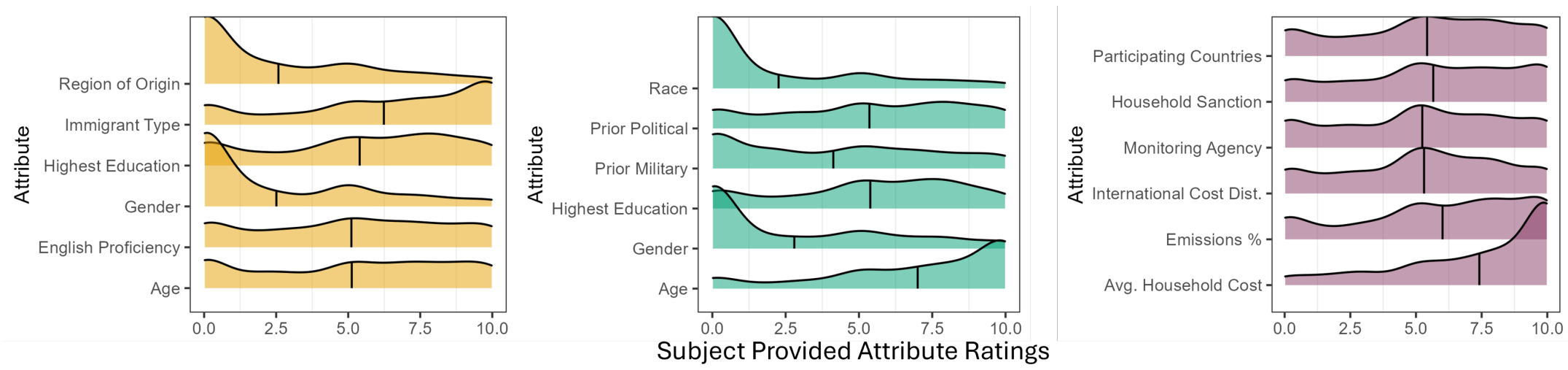

(a) No Ties

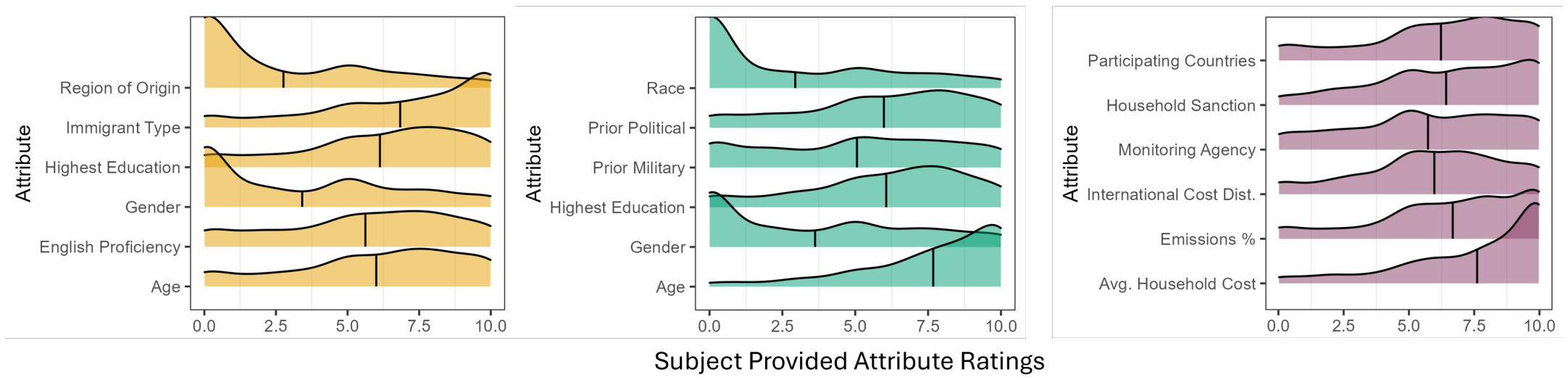

(b) Ties

Fig. S15. Distribution of subject provided attribute ratings. We plot subject provided attribute ratings (0-10) on the x-axis, where a value of 0 means "Not at all important" to their decision process and 10 means "Extremely Important." The black line represents the mean rating across subjects.

mean rating is for Immigrant Type (Asylum seeker or Economic Migrant). While the AMCEs show that Type does indeed matter, it is statistically as important as Age, Highest Education, and English Proficiency Level.

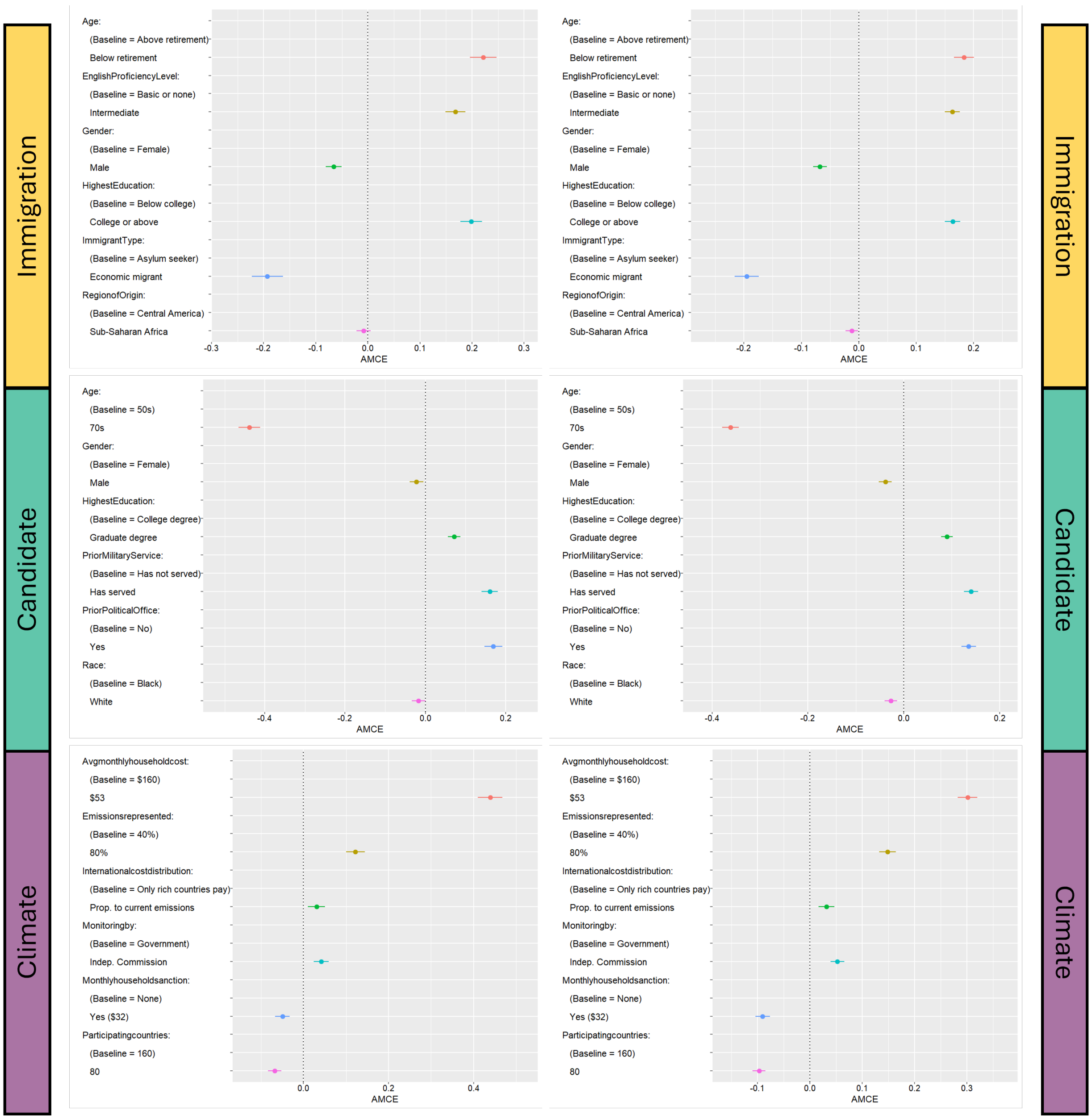

(a) No Ties **(b)** Ties

Fig. S16. The relationship between attribute levels and the probability that a given profile is chosen (AMCEs). We find that the condition-level AMCEs are comparable across both conditions. Each point can be interpreted as the relative cause impact of the given attribute level compared to the baseline: a positive value means a greater probability of choosing the profile with that attribute level and a negative value means the opposite.

D.4. Perceptions of the decision domains. Before and after each decision block, each subject provided ratings between 0 (not at all) to 10 (the most possible) on four different measures for that domain, Figure S2. We asked subjects to indicate how meaningful each domain was to them: (1) how *interested* they were in the domain, (2) how *informed* they were about the domain, and (3) how *important* the domain was. We additionally asked subjects to indicate (4) how *complicated* the decision domain was to them. We consider the average of the pre-decision and post-decision ratings in the subsequent analysis, which considers both experiments.

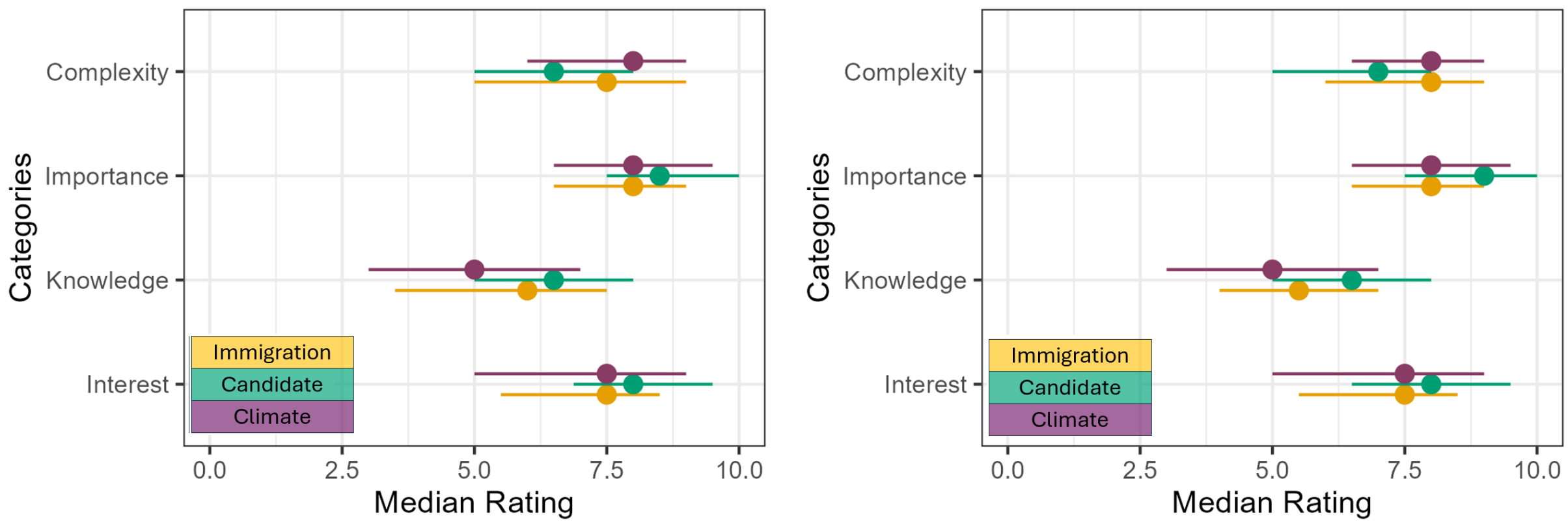

(a) No Ties

(b) Ties

Fig. S17. Subjects report that the candidate domain is more meaningful and less complex compared to the Immigration and Climate domain. Subjects in both conditions report statistically greater interest, knowledge, and importance of the candidate domain when compared to the Immigration and Climate domains (all $p < 0.001$, Wilcoxon Signed Rank Test). Conversely, subjects report the Candidate domain to be the least complex, and the Climate domain to be the most complex (all $p < 0.001$, Wilcoxon Signed Rank Test). Ratings plotted are the average of scores provided both immediately before and after decision blocks.

We found correspondence across both conditions in terms of the overall perceptions of each domain: subjects reported that they were more interested in, informed about, and considered more important the candidate domain relative to the immigration and climate domains (all $p < 0.001$, Wilcoxon Signed Rank Test), Figure S17. Interestingly the converse was true for decision complexity, where the candidate domain was rated as the least complex, and climate the most (all $p < 0.001$, Wilcoxon Signed Rank Test). Further, we found no differences between the two conditions in the meaningfulness ratings, after correcting for multiple comparisons. Interestingly, and perhaps unsurprisingly, we found that subjects reported all domains to be more complex in the ties condition, compared to the no ties condition (all $p < 0.001$, Wilcoxon Signed Rank Test).

D.5. Attribute weights as a function of strategy and levels. To better understand these multi- and bimodal distributions, we consider (1) the strategy an individual is classified as using and (2) the attribute levels that are preferred. How strategy may shape the distribution of attribute weights can be intuitively understood. For instance, someone who evaluates only one attribute (i.e., classified as TTB in the No Ties condition) will place all their weight on one attribute and little weight on any other attribute. Assuming people will differentially value attributes, this could result bimodal distributions across attributes: each attribute will be given either almost no weight or high weight among a group of subjects classified as using the TTB strategy. Alternatively, if someone evaluates all attributes and places varying weights on each attribute (i.e., WADD) that would result in more dispersed and potentially multimodal distributions. If instead they put approximately the same weights across all attributes (i.e., Tally), we would expect a unimodal distribution with each attribute having a low weight. Finally, we would also expect a unimodal distribution centered around a posterior/prior ratio of 1 for those subjects classified as Contaminant, as there should be no posterior updating. These patterns are indeed what we see in Figure S18 for both conditions, where we break down our results using the modal classified strategy for each subject. This provides a sanity check that the model is performing as it should and also serves to display how the model distinguishes between the different substantive strategies.

We next consider the presence of countervailing attitudes over attribute levels. That is, how differently are the attribute weights distributed when broken down as a function of favored attribute level (e.g., one group prefers older political candidates while the complement prefers younger candidates)? As a demonstration of how our model can infer countervailing attitudes of varying degrees for different attributes (see Table S9), below we present results from the Immigration domain No Ties condition, Figure S19. These results show that attribute weight distributions can indeed also be meaningfully decomposed by favored attribute levels: the large weight on the Type attribute is due to the fact that a sizeable group of subjects strongly favor asylum seekers *and* another group of subjects, smaller in number, strongly favor economic migrants, Figure S19a. When we consider the Age attribute, we see that those who place a large weight on this attribute tend to favor younger immigrants, with a much smaller group strongly favoring older immigrants, Figure S19b. Finally, we see effectively no differences in the region attribute: subjects neither strongly favored Central America over Sub-Saharan Africa (or vice versa) nor did they place large weights on the region attribute itself, Figure S19c.

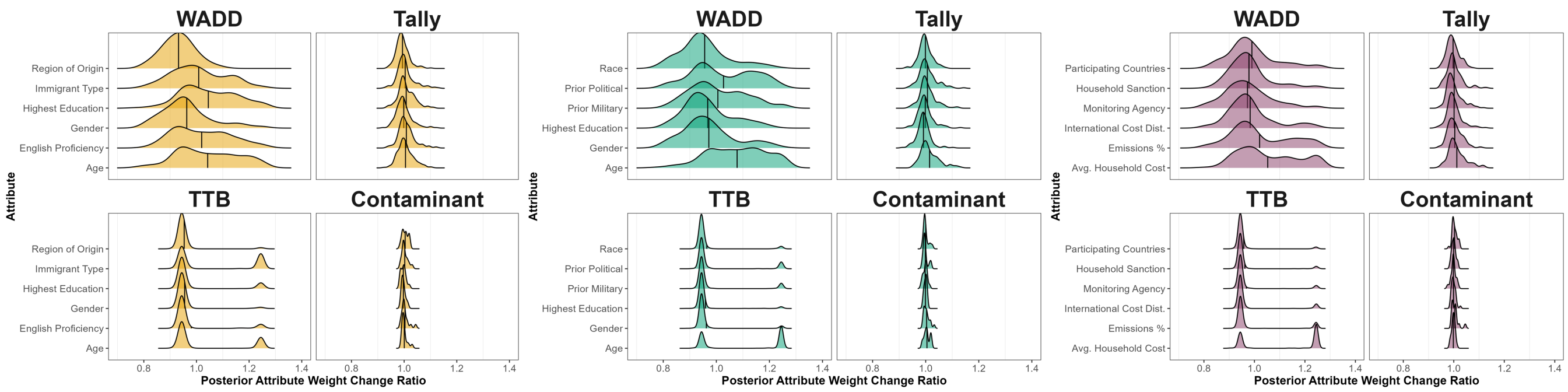

(a) No Ties

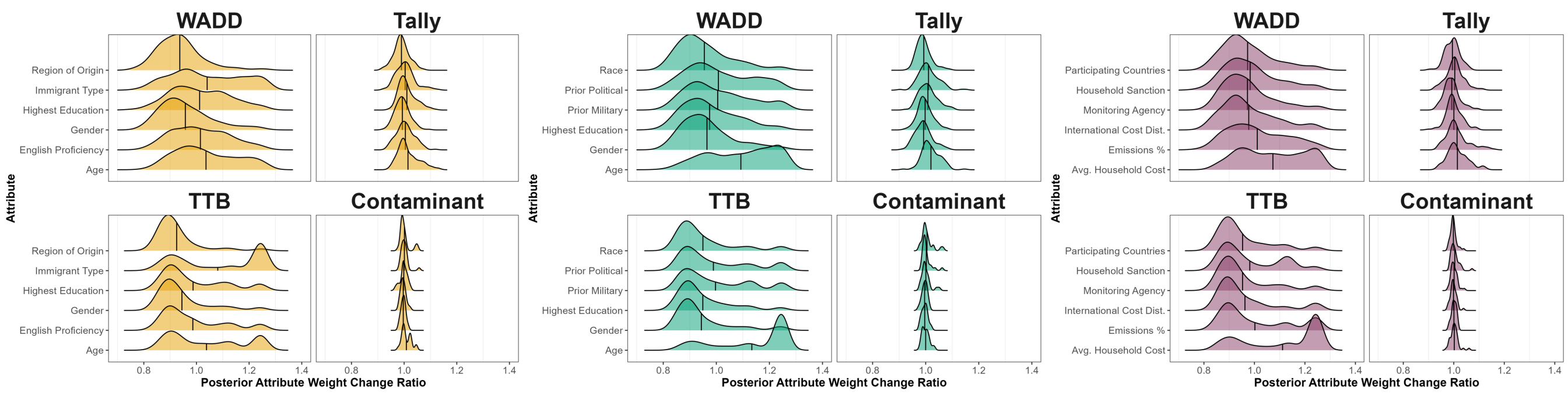

(b) Ties

Fig. S18. Attribute weights as a function of strategy classification. We plot posterior attribute change ratios on the x-axis, as in Figure 4, Main Text. Each panel contains the subjects that had a modal classification of following a given strategy: WADD (all attributes evaluated and assigned different weights), Tally (all attributes evaluated and uniform weights), TTB (highest weighted attribute that is also discriminable), or Contaminant (guessing).

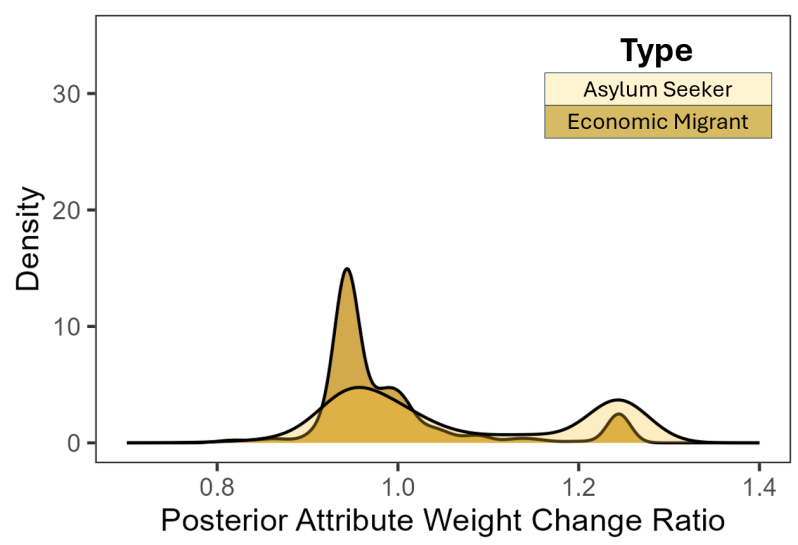

(a) Sizable countervailing

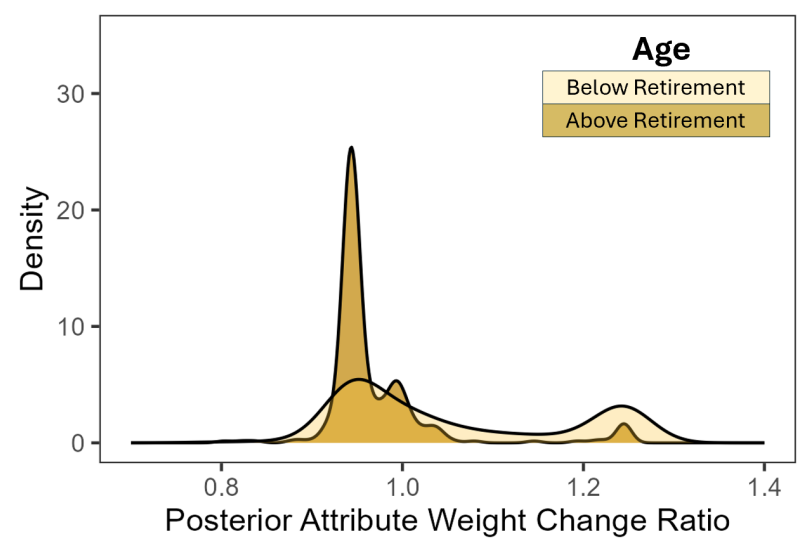

(b) Some countervailing

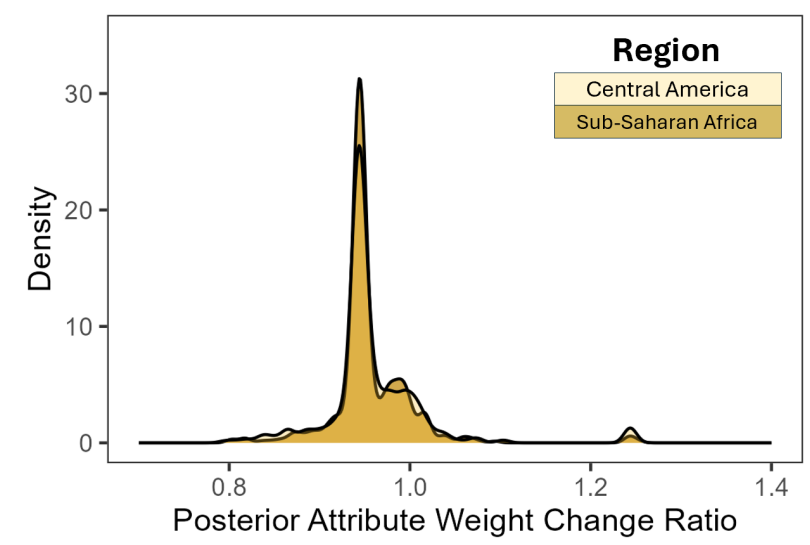

(c) No countervailing

Fig. S19. Attribute weights as a function of countervailing attitudes. We plot posterior attribute change ratios on the x-axis, as in Figure 4, Main Text. Each successive panel demonstrates reducing degrees of countervailing in the Immigration domain (No Ties). The shade of the density plot corresponds to the preferred attribute level. For example, in panel (a), the lighter distribution plots the attribute weights for all subjects who favored asylum seekers while the darker distribution plots the same but for the subjects who favored economic migrants.

E. Additional Results: Experiment 2. We find that Experiment 2 Condition 1 largely replicates Experiment 1 Condition 1, as expected. Experiment 2 Condition 2, where participants complete conjoint profile ratings trials before the forced choice, also yielded similar results to E2C1/E1C1 in the following analyses (presented graphically in the following figures): descriptive adequacy, model certainty, attribute weight distributions, countervailing, the modal number of strategies used by each subject, and meaningfulness ratings. Key points of difference include the strategy classification distributions as discussed in the Main Text (though both experimental manipulations result in less classification of TTB) and mean response time for subjects using different strategies. In contrast to the three other experimental conditions, E2C2 shows no difference in RT across all possible strategy classifications. This is, however, unsurprising due to the inclusion of rating trials which would leave minimal deliberation during the choice trial itself.

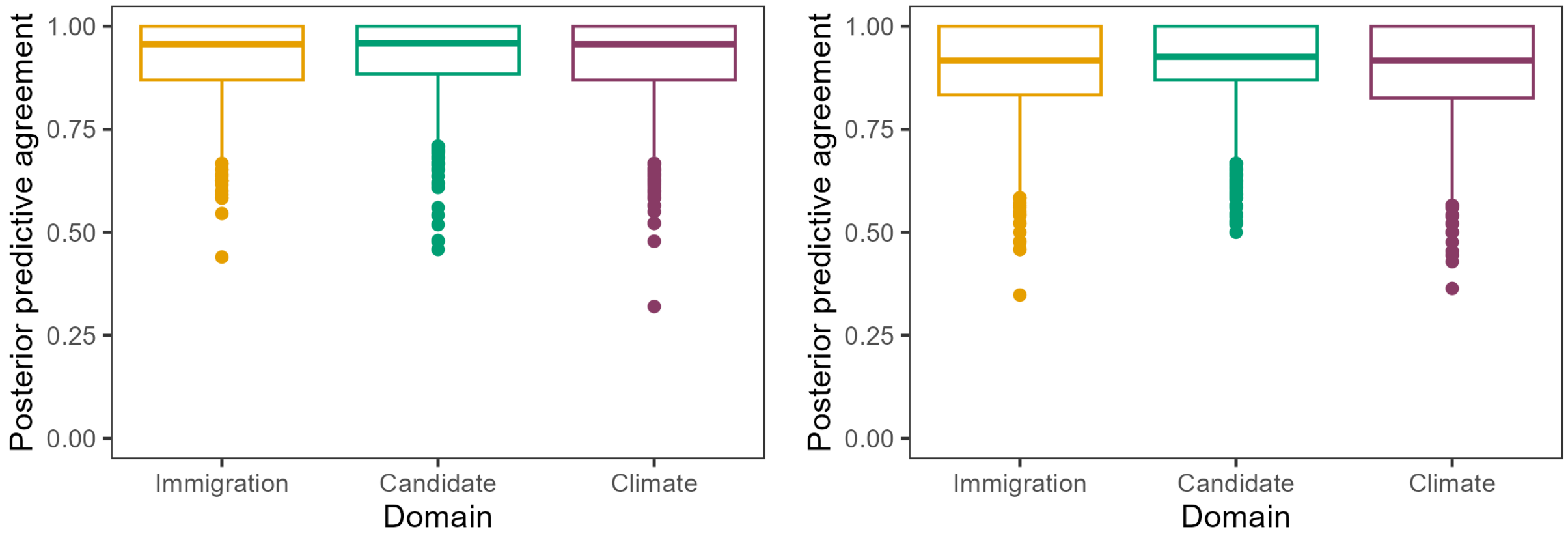

(a) Choice Only

(b) Choice and Rating

Fig. S20. E2 Descriptive adequacy. Boxplots of posterior predictive accuracy (PPA): how well the model's predicted choices align with actual observed choices. The model can at best align perfectly with observed choices ($PPA = 1$). We find comparably high PPA relative to Experiment 1.

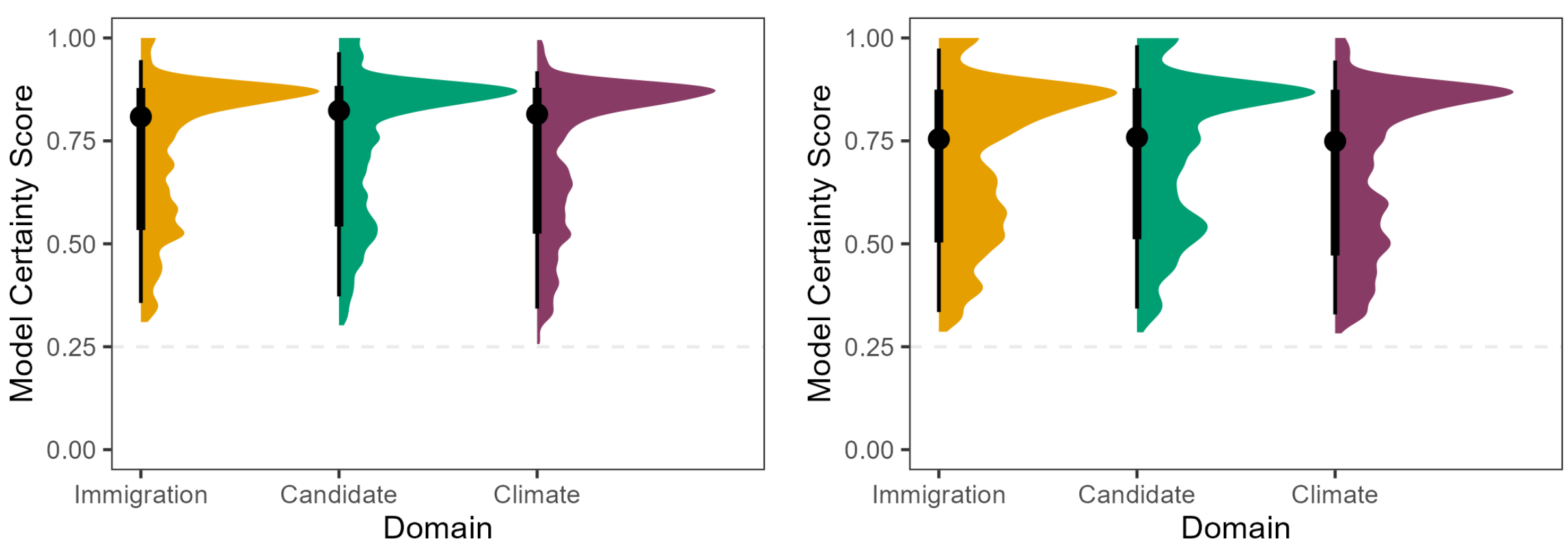

(a) Choice Only

(b) Choice and Rating

Fig. S21. E2 Model Certainty. The point estimate represents the median, with the thicker black line representing 66% of the density and the thinner black line representing 95%. The dashed grey line represents the lower limit of model certainty of 0.25 (chance).

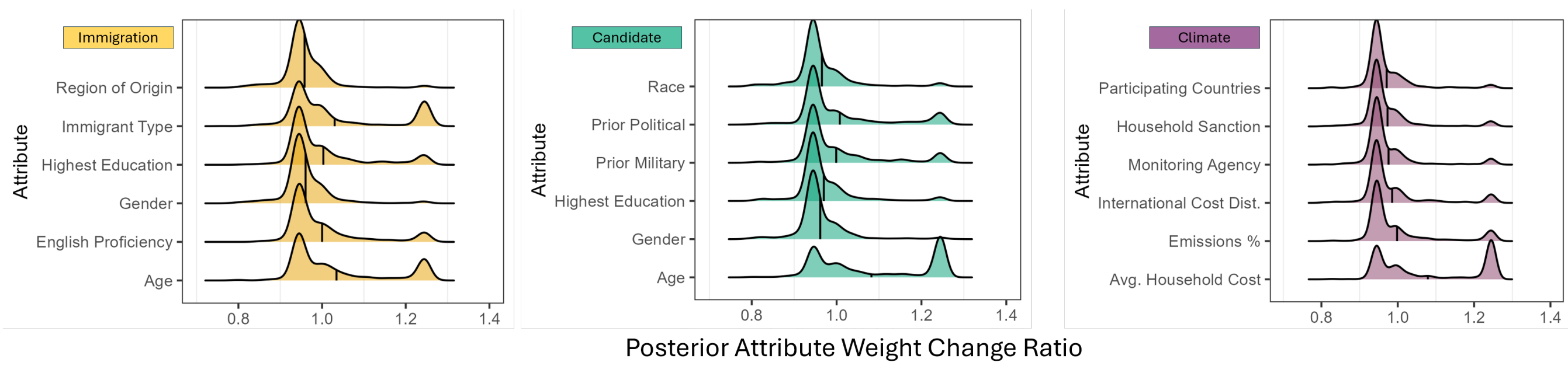

(a) Choice Only

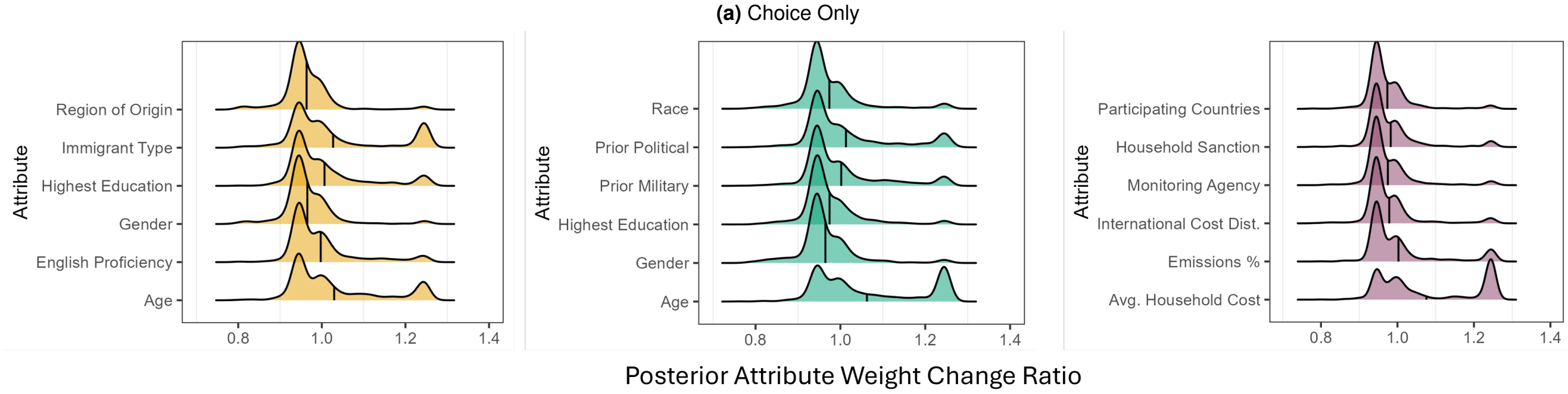

(b) Choice and Rating

Fig. S22. E2: Distribution of posterior attribute weights demonstrates heterogeneity across individuals. We plot posterior attribute change ratios on the x-axis, where a value of 1 means that the prior and posterior mean weight are the same (i.e., no updating), a value greater than 1 means that the posterior mean is greater than the prior mean, and vice versa. The black line represents the mean posterior weight across subjects.

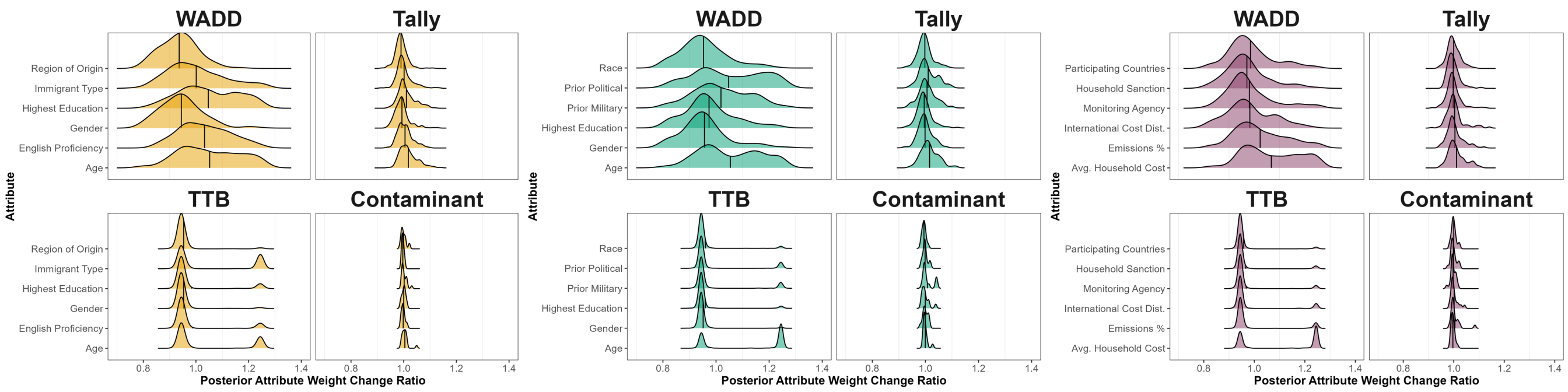

(a) Choice Only

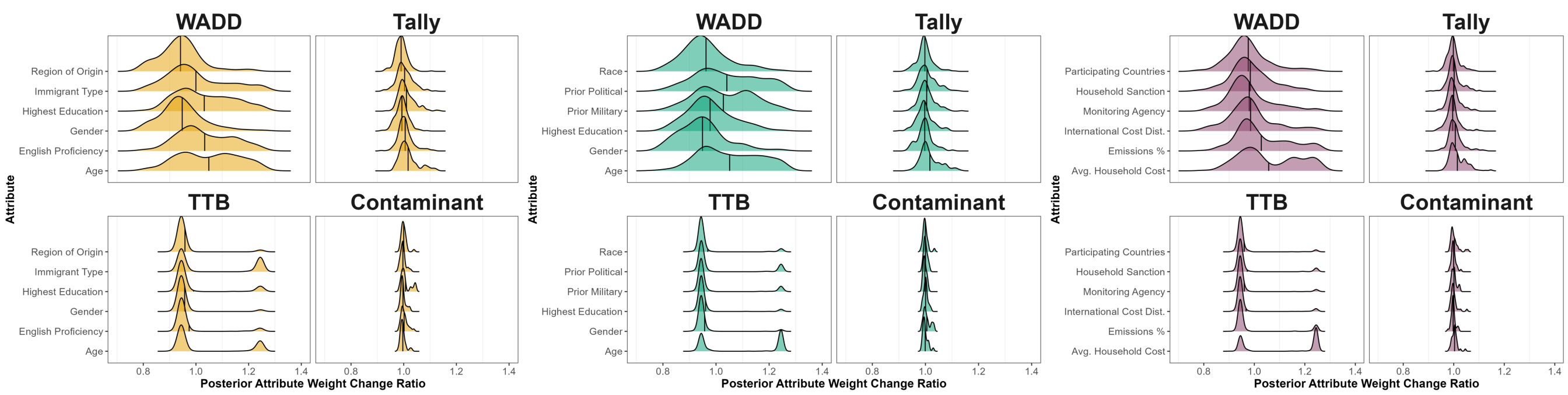

(b) Choice and Rating

Fig. S23. E2: Attribute weights as a function of strategy classification. We plot posterior attribute change ratios on the x-axis, as in Figure 4 (Main Text),S22. Each panel contains the subjects that were classified as following a given strategy: WADD (all attributes evaluated and assigned different weights), Tally (all attributes evaluated and uniform weights), TTB (highest weighted attribute that is also discriminable), or Contaminant (guessing).

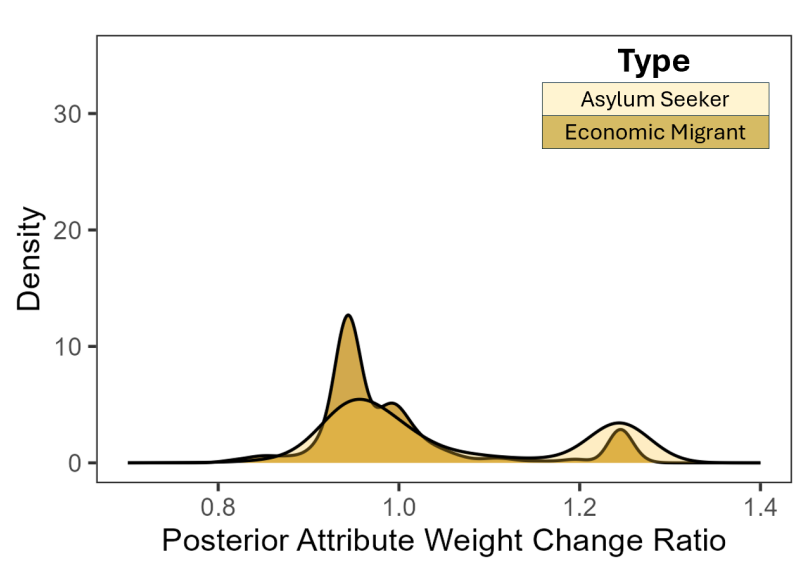

(a) Sizable countervailing

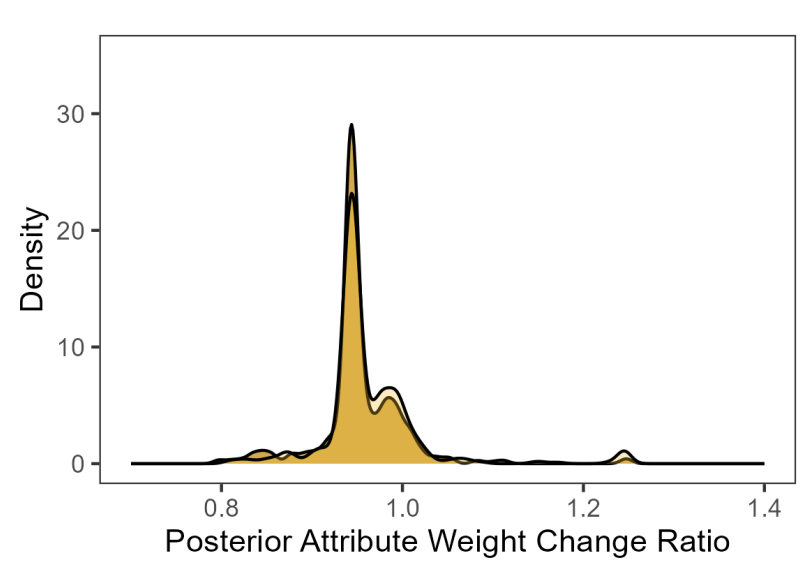

(b) Some countervailing

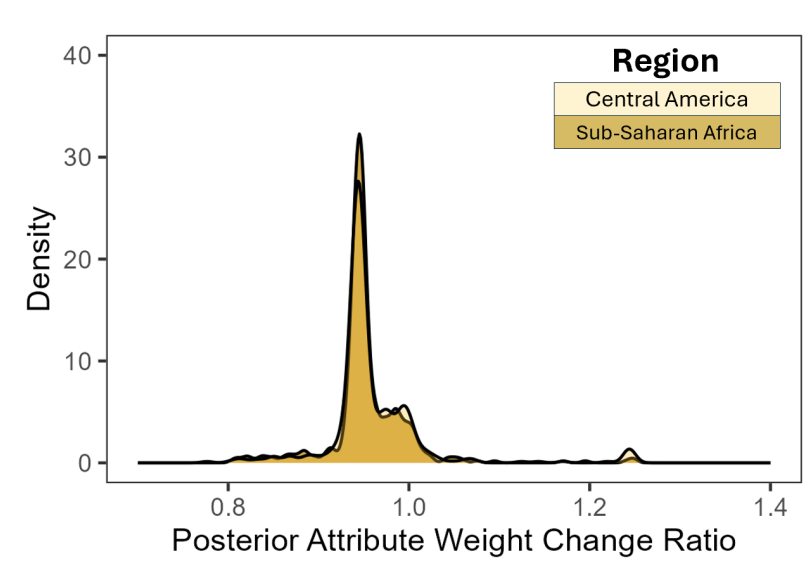

(c) No countervailing

Fig. S24. E2: Attribute weights as a function of countervailing attitudes. We plot posterior attribute change ratios on the x-axis, as in Figure 4 (Main Text),S22. Each successive panel demonstrates reducing degrees of countervailing in the Immigration domain (Choice Only). The shade of the density plot corresponds to the preferred attribute level. For example, in panel (a), the lighter distribution plots the attribute weights for all subjects who favored asylum seekers while the darker distribution plots the same but for the subjects who favored economic migrants.

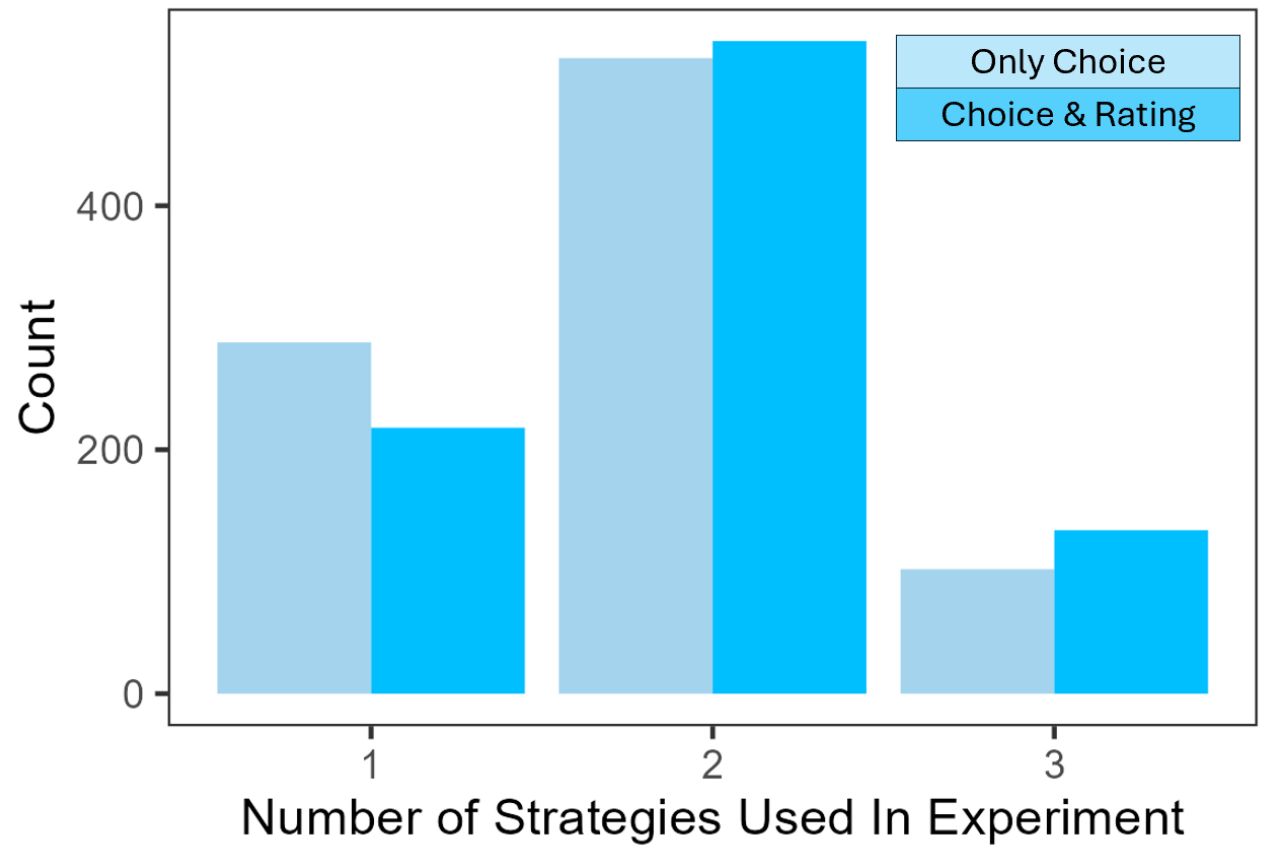

Fig. S25. E2:Most subjects do not use the same strategy for each decision domain. Subjects were directed to make choices and, in Condition 2, ratings in accordance with their preferences, with no explicit instructions given about how they should choose. As with Figure 2, Main Text, we consider the modally classified strategy for each subject in each domain.

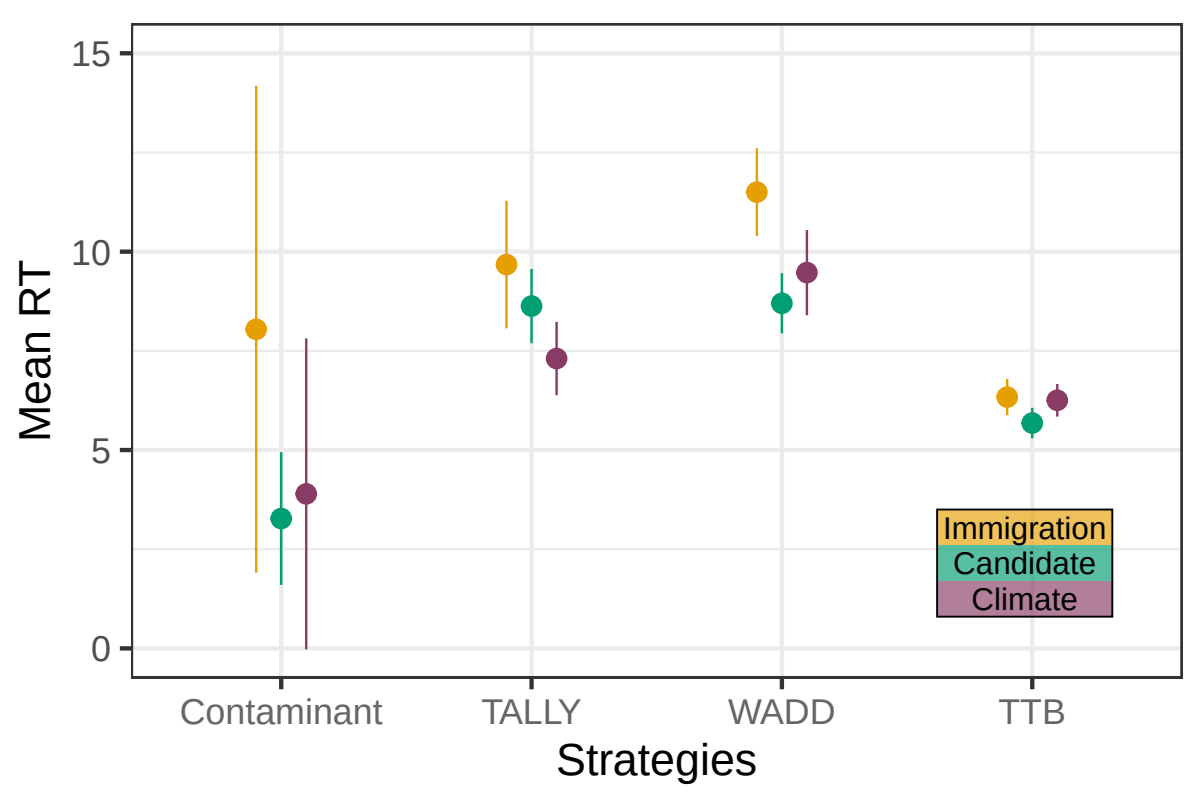

(a) Choice Only

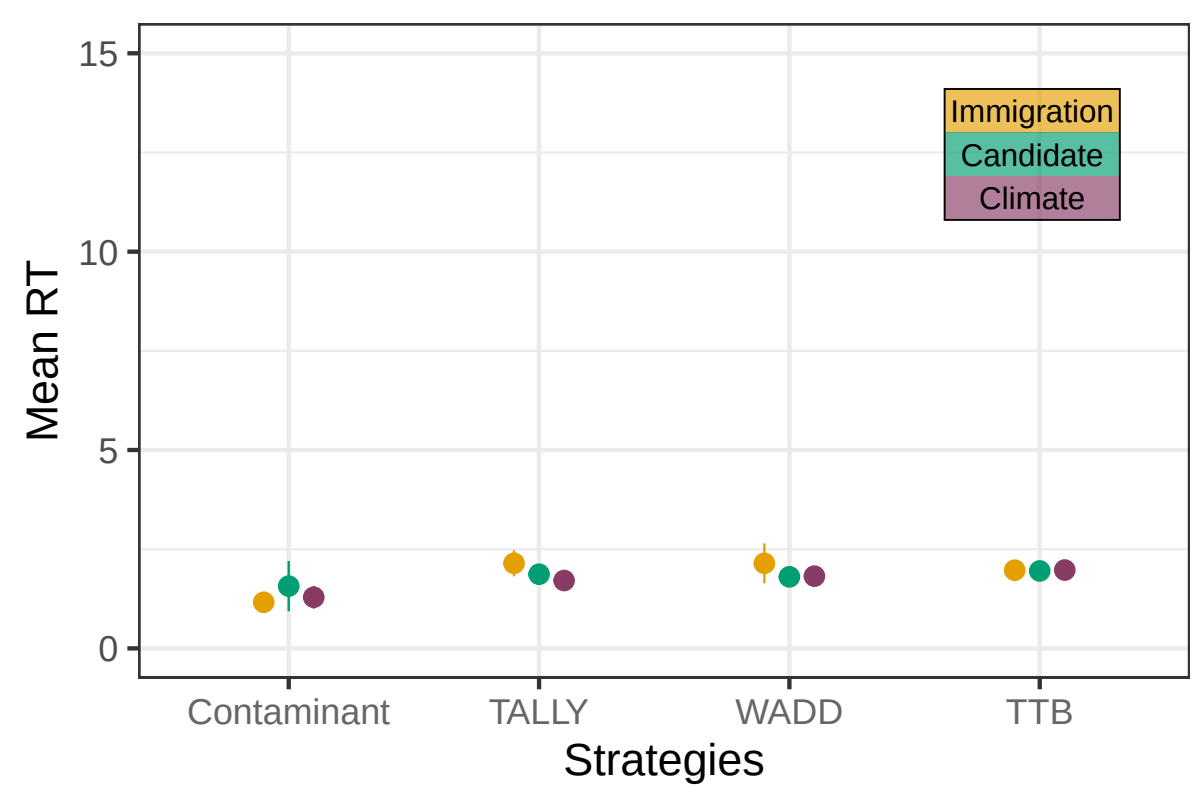

(b) Choice and Rating

Fig. S26. E2: Strategy classifications correspond with non-parametric measures (II): Response Time. "Full-information" strategies take more time than TTB in Condition 1. In Condition 2, this difference is much less apparent as even the "simpler" strategies can require the processing of several attributes to find the highest weighted *and* discriminable attribute(s). Note that the substantially lower RTs in the Choice and Rating condition are expected – if subjects were behaving in a task-congruent fashion, they would already have evaluated both profiles in the rating trials and simply had to implement their choice during the choice trial. Recall also that subjects were visually presented with their ratings for each profile as they made or implemented their 2AFC.

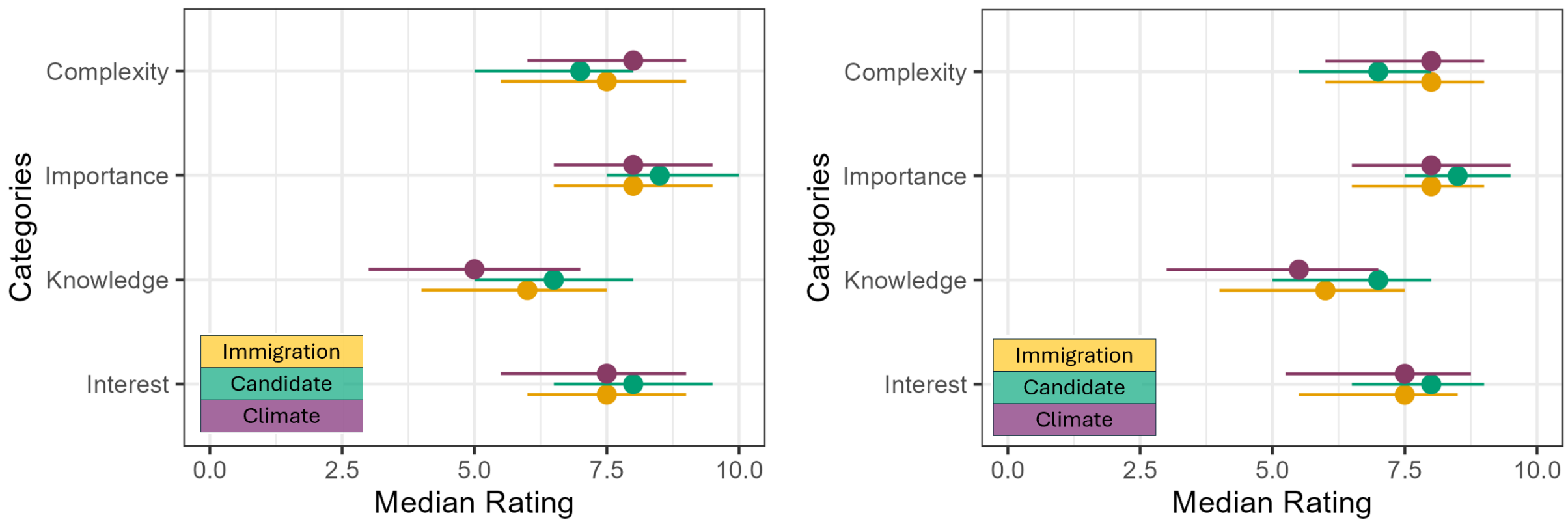

(a) Choice Only

(b) Choice and Rating

Fig. S27. E2: Subjects report that the candidate domain is more meaningful and less complex compared to the Immigration and Climate domain. Ratings plotted are the average of scores provided both immediately before and after decision blocks.

References

1. RR Lau, DP Redlawsk, Advantages and disadvantages of cognitive heuristics in political decision making. *Am. journal political science* pp. 951–971 (2001).
2. RR Lau, DP Redlawsk, *How voters decide: Information processing in election campaigns.* (Cambridge University Press), (2006).
3. M Lodge, CS Taber, *The Rationalizing Voter.* (Cambridge University Press), (2013).
4. A Tversky, D Kahneman, Judgment under uncertainty: Heuristics and biases: Biases in judgments reveal some heuristics of thinking under uncertainty. *science* **185**, 1124–1131 (1974).
5. JW Payne, JR Bettman, EJ Johnson, Adaptive strategy selection in decision making. *J. experimental psychology: Learn. Mem. Cogn.* **14**, 534 (1988).
6. M Lodge, KM McGraw, *Political judgment: Structure and process.* (University of Michigan Press), (1995).
7. WM Rahn, The role of partisan stereotypes in information processing about political candidates. *Am. J. Polit. Sci.* pp. 472–496 (1993).
8. JA Oppenheimer, Rational choice theory. *Encycl. political theory* **3**, 1150–1159 (2008).
9. G Gigerenzer, W Gaissmaier, Heuristic decision making. *Annu. review psychology* **62**, 451–482 (2011).
10. S Mousavi, G Gigerenzer, Risk, uncertainty, and heuristics. *J. Bus. Res.* **67**, 1671–1678 (2014).
11. JW Payne, JR Bettman, MF Luce, When time is money: Decision behavior under opportunity-cost time pressure. *Organ. behavior human decision processes* **66**, 131–152 (1996).
12. J Rieskamp, PE Otto, Ssl: a theory of how people learn to select strategies. *J. experimental psychology: Gen.* **135**, 207 (2006).
13. G Gigerenzer, PM Todd, Fast and frugal heuristics: The adaptive toolbox in *Simple heuristics that make us smart.* (Oxford University Press), pp. 3–34 (1999).
14. MD Zeigenfuse, MD Lee, A general latent assignment approach for modeling psychological contaminants. *J. Math. Psychol.* **54**, 352–362 (2010).
15. K Clayton, Y Horiuchi, AR Kaufman, G King, M Komisarchik, Correcting measurement error bias in conjoint survey experiments. *Am. J. Polit. Sci.* **12**, 1–11 (2023).
16. C Camerer, Individual decision making. *The handbook experimental economics* **1**, 587–704 (1995).
17. MD Lee, TD Cummins, Evidence accumulation in decision making: Unifying the “take the best” and the “rational” models. *Psychon. bulletin & review* **11**, 343–352 (2004).
18. M Plummer, , et al., Jags: A program for analysis of bayesian graphical models using gibbs sampling in *Proceedings of the 3rd international workshop on distributed statistical computing.* (Vienna, Austria), Vol. 124, pp. 1–10 (2003).
19. K Bansak, J Hainmueller, DJ Hopkins, T Yamamoto, Conjoint survey experiments in *Advances in Experimental Political Science*, eds. JN Druckman, DP Green. (Cambridge University Press), (2021).
20. Y Horiuchi, DM Smith, T Yamamoto, Identifying voter preferences for politicians’ personal attributes: A conjoint experiment in japan. *Polit. Sci. Res. Methods* **8**, 75–91 (2020).
21. SF Abramson, K Koçak, A Magazinnik, What do we learn about voter preferences from conjoint experiments? *Am. J. Polit. Sci.* **66**, 1008–1020 (2022).
22. J Hainmueller, DJ Hopkins, The hidden american immigration consensus: A conjoint analysis of attitudes toward immigrants. *Am. journal political science* **59**, 529–548 (2015).
23. K Bansak, J Hainmueller, D Hangartner, How economic, humanitarian, and religious concerns shape european attitudes toward asylum seekers. *Science* **354**, 217–222 (2016).

24. K Bansak, J Hainmueller, D Hangartner, Europeans' support for refugees of varying background is stable over time. *Nature* **620**, 849–854 (2023).
25. PA Kirkland, A Coppock, Candidate choice without party labels: New insights from conjoint survey experiments. *Polit. Behav.* **40**, 571–591 (2018).
26. MM Bechtel, KF Scheve, Mass support for global climate agreements depends on institutional design. *Proc. Natl. Acad. Sci.* **110**, 13763–13768 (2013).
27. T Bernauer, R Gampfer, How robust is public support for unilateral climate policy? *Environ. Sci. & Policy* **54**, 316–330 (2015).
28. EA Coleman, N Harring, SC Jagers, Policy attributes shape climate policy support. *Policy Stud. J.* **51**, 419–437 (2023).
29. M Lodge, R Hamill, A partisan schema for political information processing. *Am. political science review* **80**, 505–519 (1986).
30. JW Payne, JR Bettman, EJ Johnson, *The adaptive decision maker.* (Cambridge university press), (1993).
31. BE Hilbig, M Moshagen, Generalized outcome-based strategy classification: Comparing deterministic and probabilistic choice models. *Psychon. bulletin & review* **21**, 1431–1443 (2014).
32. JM Denstadli, R Lines, Conjoint respondents as adaptive decision makers. *Int. J. Mark. Res.* **49**, 117–132 (2007).
33. A Dieckmann, K Dippold, H Dietrich, Compensatory versus noncompensatory models for predicting consumer preferences. *Judgm. Decis. Mak.* **4**, 200–213 (2009).
34. JASP Team, JASP (Version 0.19.3)[Computer software] (2025).
35. S Barari, et al., Package 'cjoint'. (2023).
36. K Bansak, J Hainmueller, DJ Hopkins, T Yamamoto, Using conjoint experiments to analyze election outcomes: The essential role of the average marginal component effect. *Polit. Analysis* **31**, 500–518 (2023).
37. S Weber, N Stoop, P Van der Windt, H Zhai, A meta-analysis of attitudes towards migrants and displaced persons. *Br. J. Polit. Sci.* **55**, e168 (2025).
38. MM Aviña, et al., Which immigrants do citizens prefer? a systematic review and meta-reanalysis of 96 conjoint experiments. (2024).
39. S Schwarz, A Coppock, What have we learned about gender from candidate choice experiments? a meta-analysis of sixty-seven factorial survey experiments. *The J. Polit.* **84**, 655–668 (2022).
40. S Eshima, DM Smith, Just a number? voter evaluations of age in candidate-choice experiments. *The J. Polit.* **84**, 1856–1861 (2022).
41. K Bansak, J Hainmueller, DJ Hopkins, T Yamamoto, The number of choice tasks and survey satisficing in conjoint experiments. *Polit. Analysis* **26**, 112–119 (2018).
42. L Jenke, K Bansak, J Hainmueller, D Hangartner, Using eye-tracking to understand decision-making in conjoint experiments. *Polit. Analysis* **29**, 75–101 (2021).
43. K Bansak, L Jenke, Odd profiles in conjoint experimental designs: Effects on survey-taking attention and behavior. *Polit. Analysis* **33**, 315–338 (2025).
44. K Zhirkov, Estimating and using individual marginal component effects from conjoint experiments. *Polit. Analysis* **30**, 236–249 (2022).
45. J Hainmueller, DJ Hopkins, T Yamamoto, Causal inference in conjoint analysis: Understanding multidimensional choices via stated preference experiments. *Polit. analysis* **22**, 1–30 (2014).
